# Development of a real-time fluorescence loop-mediated isothermal amplification assay for rapid and quantitative detection of *Ustilago maydis*

**DOI:** 10.1038/s41598-017-13881-4

**Published:** 2017-10-17

**Authors:** Yanyong Cao, Lifeng Wang, Liping Duan, Jingjing Li, Juan Ma, Shuna Xie, Lei Shi, Huiyong Li

**Affiliations:** 1 Institute of Cereal Crops, Henan Academy of Agricultural Sciences, Zhengzhou, 450002 China; 2 Institute of Food Safety and Nutrition, Jinan University, Guangzhou, 510632 China; 3 Institute of Plant Protection, Henan Academy of Agricultural Sciences, Zhengzhou, 450002 China

## Abstract

The common smut of corn, caused by *Ustilago maydis* is a troublesome disease of maize. Early and accurate detection of *U*. *maydis* is essential for the disease management. In this study, primer set Pep-2 was selected for LAMP (loop-mediated isothermal amplification) from 12 sets of primers targeting three *U*. *maydis* effector genes *See1*, *Pit2* and *Pep1* according to primer screening. The optimal concentrations of *Bst* DNA polymerase and Mg^2+^ as well as inner/outer primer ratio of the LAMP reaction system were screened by combining a single factor experiment and an orthogonal design arrangement. The specificity of this real-time LAMP (RealAmp) assay was confirmed by negative testing for other pathogens. The detection sensitivity of the RealAmp assay was 200 times higher than that of detection through conventional PCR. Results of the RealAmp assay for quantifying the genomic DNA of *U. maydis* were confirmed by testing with both artificially and naturally infected samples. In addition, the RealAmp reaction could be conducted via an improved tube scanner to implement a “electricity free” assay from template preparation to quantitative detection. The resulting assay could be more convenient for use in the field as a simple, rapid, and effective technique for monitoring *U. maydis*.

## Introduction

*Ustilago maydis* is the causative agent of common smut of corn, which is considered to be one of the major prevalent diseases of maize plants in China. The average annual incidence of corn smut is between 5% and 10%. In regions where the disease is prevalent, it may occur in up to 20% of plants^1^. The hemibasidiomycete *U*. *maydis* displays a complex life cycle. To achieve successful infection, two haploid sporidia that form dikaryotic hyphae must have different alleles at both the *a* and *b* loci^2^. The most obvious symptom of infection by *U*. *maydis* is the formation of tumor-like galls that are found most frequently on ears, tassels, or sometimes on other meristematic tissues^3^. The tumors provide the environment where the hyphae differentiate, culminating in the formation of masses of black teliospores. Teliospores are probably the major source of the dispersal of inoculum in the field. Under the appropriate conditions, teliospores can germinate, undergo meiosis, and produce more haploid progeny^4,5^. Host resistance is the only practical method of controlling common smut in areas where *U*. *maydis* is prevalent^3^. To the best of our knowledge, there are currently no cultivated varieties or lines of maize available that have been shown to be immune to *U*. *maydis* once plants have become infected^3^. Maize common smut is still a major threat to maize production and hence a method of early, efficient, and specific detection of *U*. *maydis* could help improve the efficiency of management and resistance breeding with respect to this disease.

Many of the molecular assays currently employed for detecting *U. maydis* are based on the conventional PCR assay. Primers for PCR have been designed based on the insert terminal regions of a *U*. *maydis* plasmid library or the specific region downstream of the homeodomain of the *bE* gene at the *b* locus, respectively^6,7^. For the detection of another *Ustilago* species, *U*. *scitaminea* (also referred to as *S. scitamineum*), PCR-based methods were reported in which primers were designed based either on the *bE* mating-type gene sequence of *U. maydis* ^8^ or on the internal transcribed spacer (ITS) sequence of *S*. *scitamineum* ^9^. PCR assays using ITS sequence have also been reported for detecting the pathogen of barley smut, *U*. *hordei* ^10^. A Taq-man real-time PCR-based assay was developed for the early detection and identification of *S*. *scitamineum* using primers specific to the *bE* gene and a Taqman probe^11^. These methods may be specific for detecting *U*. *maydis* and other pathogenic fungi which share a similar life cycle or induce symptoms similar to smut disease. However, they fail to provide a rapid and quantitative tool capable of diagnosing the pathogen in crop and soil samples directly. Thus, a rapid, sensitive, simple, and economical detection method for practical applications would be preferred. Recently, two research groups successfully developed LAMP (loop-mediated isothermal amplification)-based assays for the detection of *S*. *scitamineum* in which internal transcribed spacer sequences and the genomic sequence of the *pep1* gene were selected as targets for LAMP primer design, respectively^12,13^. These two LAMP protocols provided a specific, sensitive, and rapid test for the detection of *S*. *scitamineum* infection in sugarcane.

In addition to being an important agricultural pathogen of global significance, *U*. *maydis* has served as an excellent model for the study of the molecular interactions between biotrophic basidiomycete fungal plant pathogens and their host plants^14,15^. *U*. *maydis* deploys many effector proteins during establishment of infection and development of symptoms in maize plants^16^. Pep1 (protein essential for penetration1) is involved in the successful penetration of fungal hyphae into plant tissue and the establishment of initial compatibility by targeting and inhibiting the activity of the plant peroxidase POX12^17,18^. Another effector, Pit2 (protein involved in tumor2), is essential for the maintenance of biotrophy and the induction of tumors It has also been shown to be involved in the suppression of host defense response by its inhibition of maize apoplastic cysteine proteases^19,20^. The recently identified effector See1 (seedling efficient effector1) directly and specifically affects the formation of leaf tumors in maize by interfering with the MAPK-triggered phosphorylation of a maize homolog of SGT1 (suppressor of allele of skp1)^16^.

The LAMP assay is a novel nucleic acid amplification method that is performed under isothermal conditions utilizing *Bst* DNA polymerase, which has strand-displacement activity. A set of four or six custom-designed primers, which recognize at least six distinct sequences on the target DNA, are used to generate amplification products that contain single-stranded loops thereby allowing primers to bind to these sequences without the need for repeated cycles of thermal denaturation^21–23^. The detection of LAMP products by way of a fluorescent dye can be quantified using a real-time PCR thermal cycler or a portable fluorescent reader called ESE-Quant Tube Scanner, which is equipped with a battery pack. The latter is advantageous in that it offers a convenient method for rapid onsite detection^24–27^. Real-time fluorescence LAMP (RealAmp) is currently employed for quantitative assays of soil-borne pathogens such as *Fusarium oxysporum* f. sp. *cubense* and *Pythium inflatum* ^26,27^.

The objectives of this study were to develop a *U*. *maydis*-specific RealAmp assay and to evaluate this assay for the rapid and direct quantitative detection of *U*. *maydis* in naturally infested soil samples or maize plants prior to planting or before symptoms of smut disease appear. Genomic sequences of *U*. *maydis* effector genes *Pep1*, *Pit2* and *See1* were used to design candidate primers for screening to determine the optimal primer set. A single-factor experiment combined with an orthogonal experimental design was conducted in order to optimize three important reaction components (quantity of *Bst* DNA polymerase, primer ratio, and Mg^2+^ concentration) of the LAMP reaction system. The specificity and sensitivity of the method were evaluated and the feasibility of the LAMP-based quantitative detection assay was corroborated by testing with artificially and naturally infested samples. The optimized RealAmp method that was established from this study might lay a foundation for research on *U*. *maydis*-maize plant molecular interactions and on common smut disease management and resistance breeding.

## Results

### LAMP primer screening

In order to screen for an optimal target gene for use in the LAMP assay, a total of 12 sets of LAMP primers were designed that targeted three *U*. *maydis* effector genes, which included *UmPep1*, *UmPit2* and *UmSee1*. The DNA of another maize pathogen, *S. reiliana* was used as negative control template.

**Figure 1:**
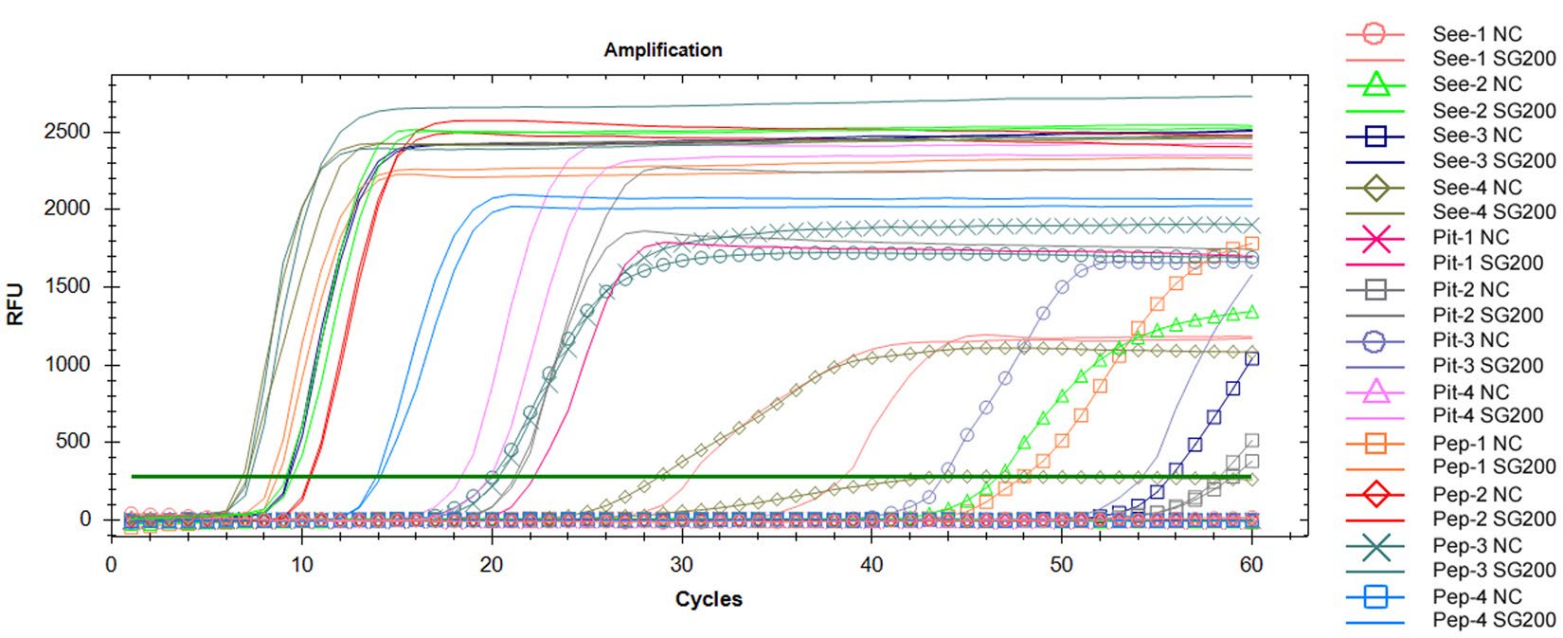
Screening of *Ustilago maydis* LAMP primers. The LAMP fluorescence signal (RFU) *vs*. Cq [threshold time (min)] amplification curve graph was plotted automatically by the Bio-Rad CFX96 Real-Time PCR system. 12 sets of primers that targeted one of three *U*. *maydis* effector genes (*See1*, *Pit2* and *Pep1*) were designed for the primers optimization experiment. 1–4 indicates one of the four sets of primers designed for each gene; NC indicates the reaction using negative control templates (the DNA of another maize pathogens, *Sporisorium reiliana*, which causes symptoms similar to corn common smut).

For the LAMP primer sets Pep-1, Pep-3, Pit-2, Pit-3, See-2, See-3 and See-4, pseudo-positive amplification was observed (i.e. isothermal amplification occurred in both reaction mixtures containing either SG200 or *S. reiliana* genomic DNA), indicating that they are not specific to *U. maydis* DNA (Fig. 1). For the primer sets Pep-4 and See-1, the LAMP amplification curve indicated that the corresponding target segments were amplified only from the SG200 DNA (Fig. 1). However, melt peak data from the same reaction mixtures indicated that some amplification was occurring in the negative control DNA samples (Supplementary Fig. S1). Similar melt peaks were also observed in the negative control LAMP reactions for primer sets Pep-1, Pep-3, Pit-2, Pit-3, See-2, See-3 and See-4 (Supplementary Fig. S1).

Based on the above data, we speculated that Pit-1, Pit-4 and Pep-2 might be suitable for the LAMP assay specific to *U*. *maydis*. Further analysis indicated that the Pep-2 primer set is the most optimal of these three candidates for use in the LAMP assay as it yields a lower Cq value (reaction threshold time, 10.37 min), and has better repeatability (Fig. 1). Therefore, Pep-2 was selected for the further optimization of the LAMP assay of *U*. *maydis* in this study. The sequences of the final set of primers used in the LAMP protocol and their respective target sites are indicated in Supplementary Fig. S2.

### Optimization of LAMP reaction

**Figure 2:**
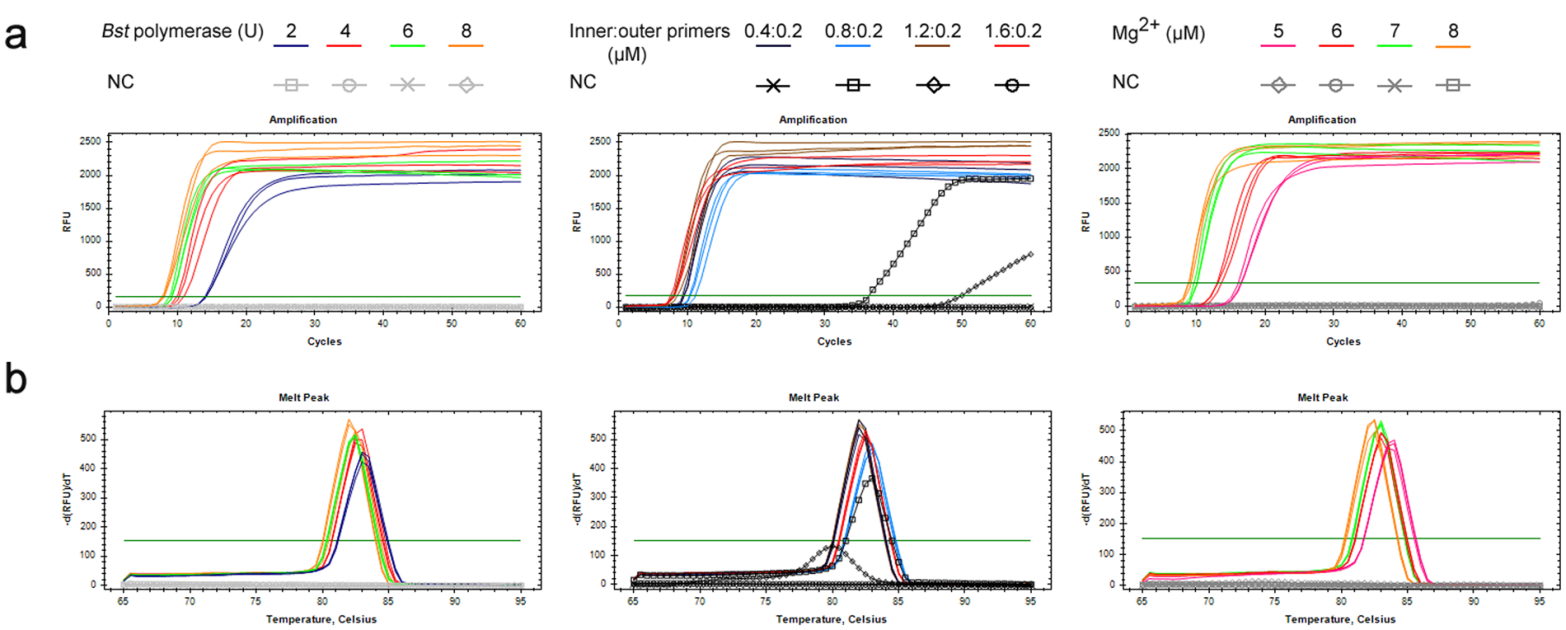
Single factor experiment of RealAmp. The influence of each variable on the LAMP reaction was analyzed by the amplification curve (**a**) and the melt peak (**b**). For the *Bst* DNA polymerase optimization, *Bst* polymerase quantities were adjusted to 2 U, 4 U, 6 U and 8 U, respectively. Ratios of inner to outer primers were set at 2:1, 4:1, 6:1 and 8:1 with the outer primer concentration fixed to 0.2 μM to optimize the primer ratios. Mg^2+^ concentrations in the LAMP reactions were varied from 5 mM, to 8 mM for the optimization of Mg^2+^.

The screening of candidate LAMP primers outlined above was based on our previous work^27^. In order to test whether the LAMP reaction system for *U*. *maydis* could be improved, modifications to three parameters of LAMP reaction mixture (quantity of *Bst* DNA polymerase, the ratio of the concentration of inner primer pair FIB/BIP to outer primer pair F3/B3, and concentration of Mg^2+^) were screened (Fig. 2). In evaluating the effect of quantity of *Bst* enzyme, it was shown that the Cq value of the amplification decreased as the amount of *Bst* enzyme was increased in the reaction mixture while keeping the other parameters constant (concentrations of Mg^2+^, FIP/BIP, and F3/B3 were fixed to 8 mM, 1.6 μM and 0.2 μM, respectively). The reaction time differed by 7 min between samples containing either 2 U or 8 U *Bst* DNA polymerase (Fig. 2a). In testing the concentration ratio of inner primers to outer primers, non-specific amplification was observed when the ratio was set at 4:1 and 6:1. The reaction reached its highest amplification efficiency when the ratio was adjusted to 8:1 (Fig. 2b). For the optimization of Mg^2+^, the average threshold time at a concentration of 5 mM was 16.3 min. The amplification efficiency exhibited a sharp increase when the Mg^2+^ was adjusted to 6 mM or 7 mM and remained high up to 8 mM MgSO_4_ (Fig. 2c).

**Figure 3:**
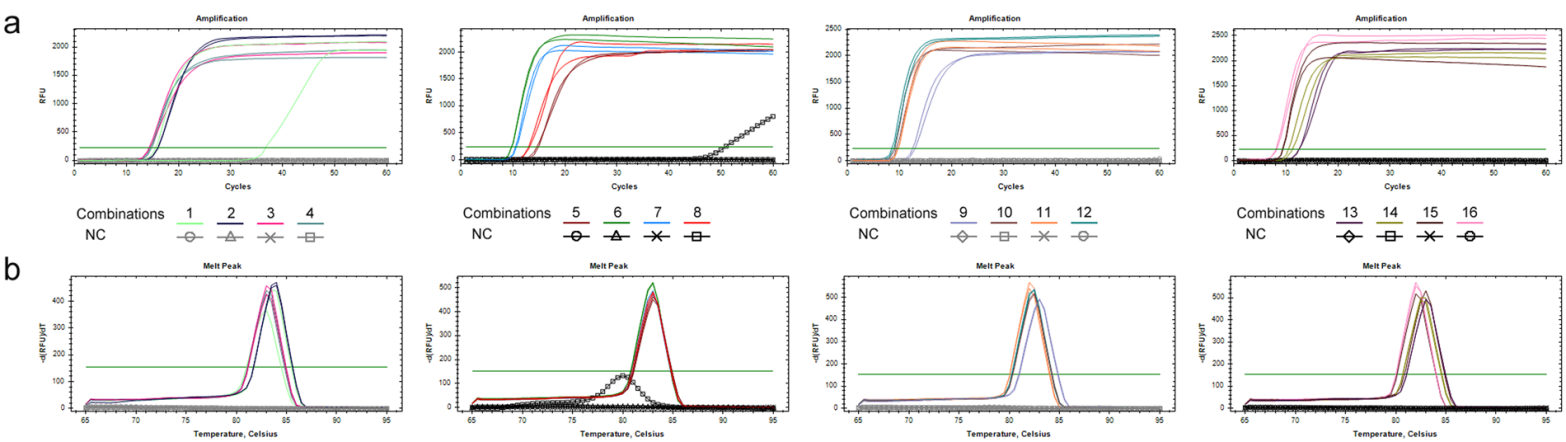
Reaction results based on different combinations of reaction components according to an orthogonal test. (**a**) Reactions of 16 combinations monitored by the amplification curves. (**b**) Corresponding melt peaks of the above reactions. 1–16 represent each combination performed in the orthogonal test shown in Table 1. NC indicates reactions that share the same conditions with each combination except for the substitution of a negative control template (genomic DNA of *Sporisorium reiliana*).

Based on the results of the single-parameter experiments, further LAMP analysis was performed with 16 combinations using an orthogonal experimental design (Table 1). Amongst the 16 combinations, the negative control reactions for combinations 8 and 12 showed false positive amplification, combinations 1, 9, and 10 resulted in poor reproducibility according to the amplification curves or melt peaks (Fig. 3), and combinations 2, 3, 4, 5, 7, 13, and 14 yielded sub-optimal results in terms of reaction threshold time ( > 10 min) (Fig. 3). The threshold times of combinations 6, 11, 15 were between 9 and10 min, whereas that of combination 16 was approximately 8 min; plus, this reaction combination exhibited good repeatability (Fig. 3). Taking into consideration the previously performed single parameter experiments along with the orthogonal experimental setup, combination 16 was considered to represent the optimal conditions for a LAMP system used to detect *U*. *maydis*. This optimized reaction mixture (total, 25 μl) contained 12.5 μl 2 × *Bst* DNA polymerase (NEB, Ipswich, MA, USA) reaction buffer [1 × containing 1.6 mM dNTPs, 1 M betaine, 4 mM MgSO_4_, 20 mM Tris-HCl (pH 8.8), 10 mM KCl, 10 mM (NH4)_2_SO_4_, and 0.1% Triton X-20 (Sigma-Aldrich Inc., Saint Louis, USA), Double Helix Tech. Inc., Guangzhou, China], 0.2 μM SYTO-9 fluorescent dye (Invitrogen, Carlsbad, CA, USA), 2 μl of the template DNA, 4 mM MgSO_4_, 1.6 μM each of FIP and BIP, 0.2 μM each of F3 and B3, 0.8 μM each of loop primers LF3 and LB3, and 8 U of *Bst* DNA polymerase (NEB).

**Table 1:** [L_16_(4^5^)] orthogonal design for *Ustilago maydis* LAMP system

Combination	Factor		
	*Bst* DNA polymerase concentration (U)	inner vs outer primer (μM)	Mg^2+^ concentration (mM)
1	2	0.8: 0.2	5
2	4	1.2: 0.2	5
3	6	1.6: 0.2	5
4	8	0.4: 0.2	5
5	2	0.4: 0.2	6
6	4	1.6: 0.2	6
7	6	1.2: 0.2	6
8	8	0.8: 0.2	6
9	2	1.6: 0.2	7
10	4	0.8: 0.2	7
11	6	0.4: 0.2	7
12	8	1.2: 0.2	7
13	2	1.2: 0.2	8
14	4	0.4: 0.2	8
15	6	0.8: 0.2	8
16	8	1.6: 0.2	8

### Specificity test of LAMP reaction system

To investigate the specificity of LAMP primer set Pep-2 and the optimized LAMP reaction for *U*. *maydis*, six other pathogens that infect maize plants and one peanut pathogen *Colletotrichum truncatum* (which has an overwintering pattern similar to *U*. *maydis*) were tested using this same system.

**Figure 4:**
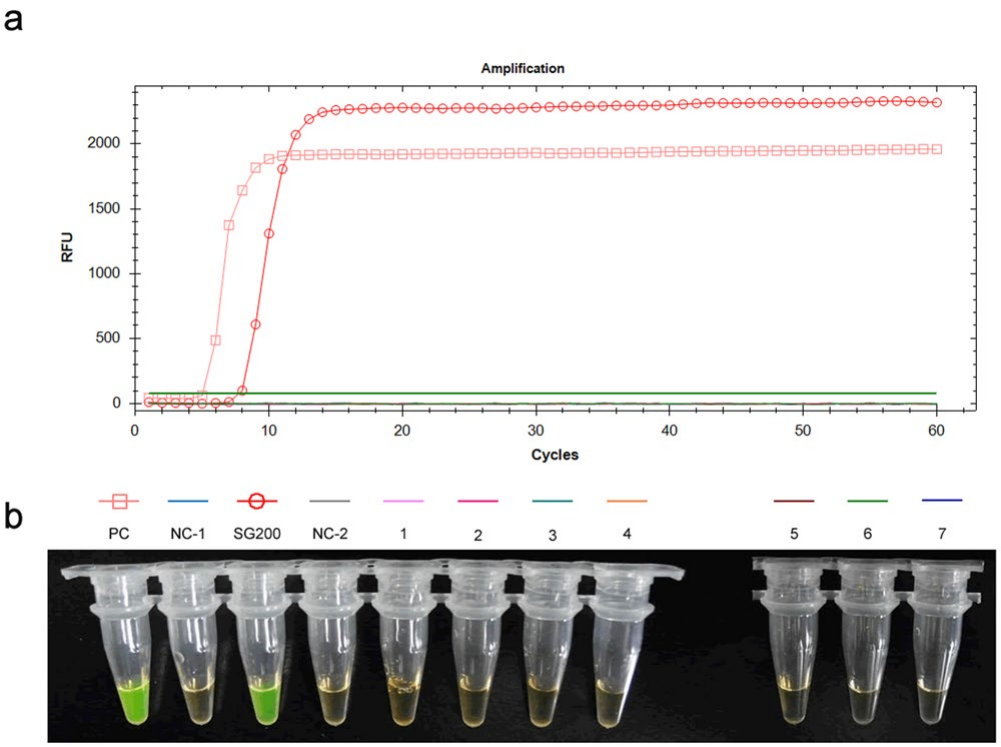
Specificity test of RealAmp assay for the detection of *Ustilago maydis*. (**a**) The RealAmp fluorescence (RFU) *vs*. Cq [threshold time (min)] amplification curve graph was plotted automatically by the Bio-Rad CFX96 Real-Time PCR system. (**b**) Visual inspection of the RealAmp amplification products using SYBR Green I. The original orange color of SYBR green turned to green in a reaction mixture showing a positive result. The templates consisted of genomic DNA isolated from the following controls and other pathogens: PC, positive control (*Staphylococcus aureus*); NC-1, negative control 1 (*Salmonella choleraesusi*); NC-2, negative control 2 (*S*. *bongori*); SG200, *Ustilago maydis* strain SG-200; 1, *Curvularia lunata*; 2, *Bipolaris maydis*; 3, *Colletotrichum truncatum*; 4, *Sporisorium reiliana*; 5, *Fusarium moniliforme*; 6, *F*. *graminearum*; 7, *Pythium inflatum*.

The reaction curves indicated that the Pep-2 primer set was able to specifically amplify the target DNA sequence of *U*. *maydis* strains SG200 and A1, but not the DNA of the other plant pathogens that were evaluated (Fig. 4a; Supplementary Fig. S3). By visual assessment, the color of the LAMP reaction products changed from orange to green when *U*. *maydis* and the positive control were detected with SYBR Green I, whereas the color remained orange for the negative control, for the other maize pathogens, and for the peanut pathogen (Fig. 4b).

To evaluate the stability of our optimized *U*. *maydis* LAMP reaction system, a 100 × diluted template DNA was also evaluated in parallel with the specificity test experiment. The data showed that amplification curves of LAMP products were similar to those of using the undiluted templates (Supplementary Fig. S3).

### Sensitivity of LAMP

**Figure 5:**
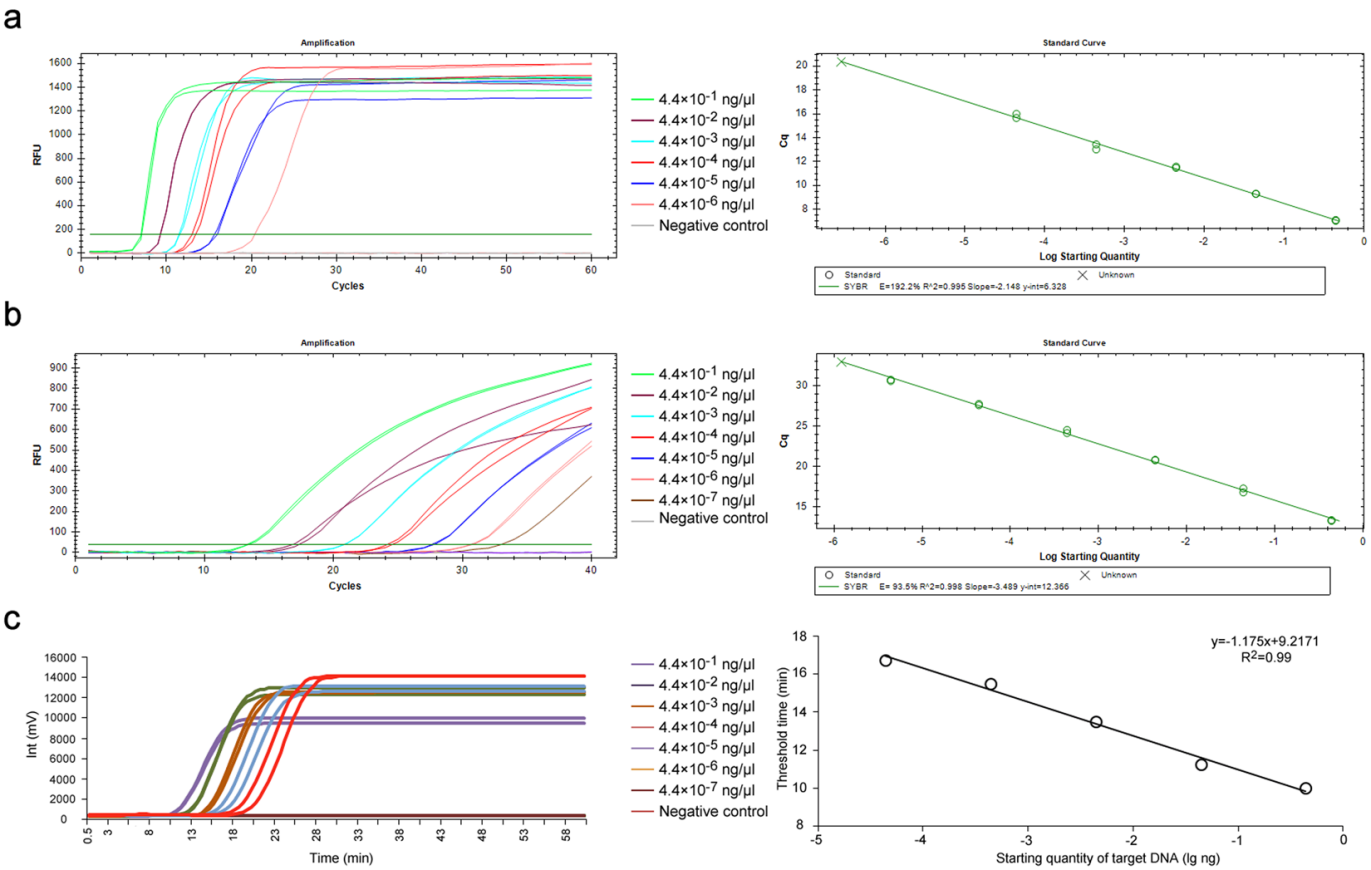
Sensitivity comparison of *Ustilago maydis* RealAmp and real-time PCR using the SG200 strain plasmid DNA as template. (**a**) Sensitivity of the RealAmp assay conducted on the Bio-Rad CFX96 Real-Time PCR system. The RealAmp fluorescence (RFU) *vs*. Cq [threshold time (min)] amplification curve graph and standard curve in which threshold time was plotted against the logarithmic amount of initial template DNA were plotted automatically by the CFX96 Real-Time PCR system. (**b**) Sensitivity test of real-time PCR for detecting *U*. *maydis*. The real-time fluorescence units were plotted against the initial concentration of plasmid DNA ranging from 4.4 × 10^−1^ ng/μl to 4.4 × 10^−7^ ng/μl by the CFX96 Real-Time PCR system. The standard curve was generated using known Log_10_-transformed concentrations of a 10-fold serial dilution of the pEasy-Pep plasmid DNA and the corresponding threshold cycle (Ct) values. (**c**) RealAmp sensitivity test using ZYD-S1 instrument. The fluorescence *vs*. time amplification curves of RealAmp were plotted automatically by the ZYD-S1 Tube Scanner. The standard curve was calculated by ZYD-S1 in which threshold time (*Tt*) was plotted against the logarithmic amount of initial template plasmid DNA.

For the sensitivity tests, 10-fold serial dilutions of the pEasy-Pep plasmid DNA mixed with either DNA isolated from soil or maize genomic DNA were used as a reference. The preliminary experiment showed that amplification of LAMP products with exponential growth occurred within the range from 0.44 ng/μl to 4.4 × 10^−5^ ng/μl of plasmid DNA (Supplementary Fig. S4). To further determine the detection limit of *U*. *maydis* using RealAmp, an experiment was conducted using serial dilutions of the pEasy-Pep plasmid ranging from 0.44 ng/μl to 4.4 × 10^−8^ ng/μl. Amplification results indicated that the RealAmp assay could detect concentrations as low as approximately 44 fg/μl of plasmid DNA when mixed with total DNA extracted from soil or maize plants (Fig. 5a), which is in agreement with the preliminary experiment. Consequently, a standard curve between the Cq-value and the logarithm concentrations of template plasmid DNA was constructed (Fig. 5a).

To compare the sensitivity between the RealAmp assay and conventional real-time PCR, serially diluted template DNA samples were used to conduct a real-time PCR experiment. This experiment detected amplification of PCR products and, in light of the regression analysis, plotting Cq against initial template concentrations showed that the resulting standard curve was linear over a concentration range of at least six orders of magnitude, from 10^−3^ to 10^−8^. Thus, the detection limit of the real-time PCR was approximately 4.4 fg/μl plasmid DNA (Fig. 5b). The sensitivity of the RealAmp and real-time PCR assays for detecting *U*. *maydis* were further verified by a detection limit experiment, in which reactions using 10^−7^/10^−8^ and 10^−8^/10^−9^ concentrations of the original plasmid template were repeated 30 times and 20 times, respectively (Supplementary Fig. S5).

The sensitivity of RealAmp was also tested using the ZYD-S1^TM^ detection system in this study. Curves representing the standard fluorescence amplification of LAMP products with exponential growth were obtained when employing pEasy-Pep template DNA ranging from 440 ng/μl to 44 fg/μl (Fig. 5c). These results indicated that the detection limit of the *U*. *maydis* RealAmp system is approximately equal to that of LAMP using the BioRad Real-Time system, thereby confirming that amplification was reliable and the RealAmp assay employing the ZYD-S1^TM^ instrument could be used for pathogen DNA quantification through traditional standard curves.

### RealAmp detection of artificially inoculated samples

**Figure 6:**
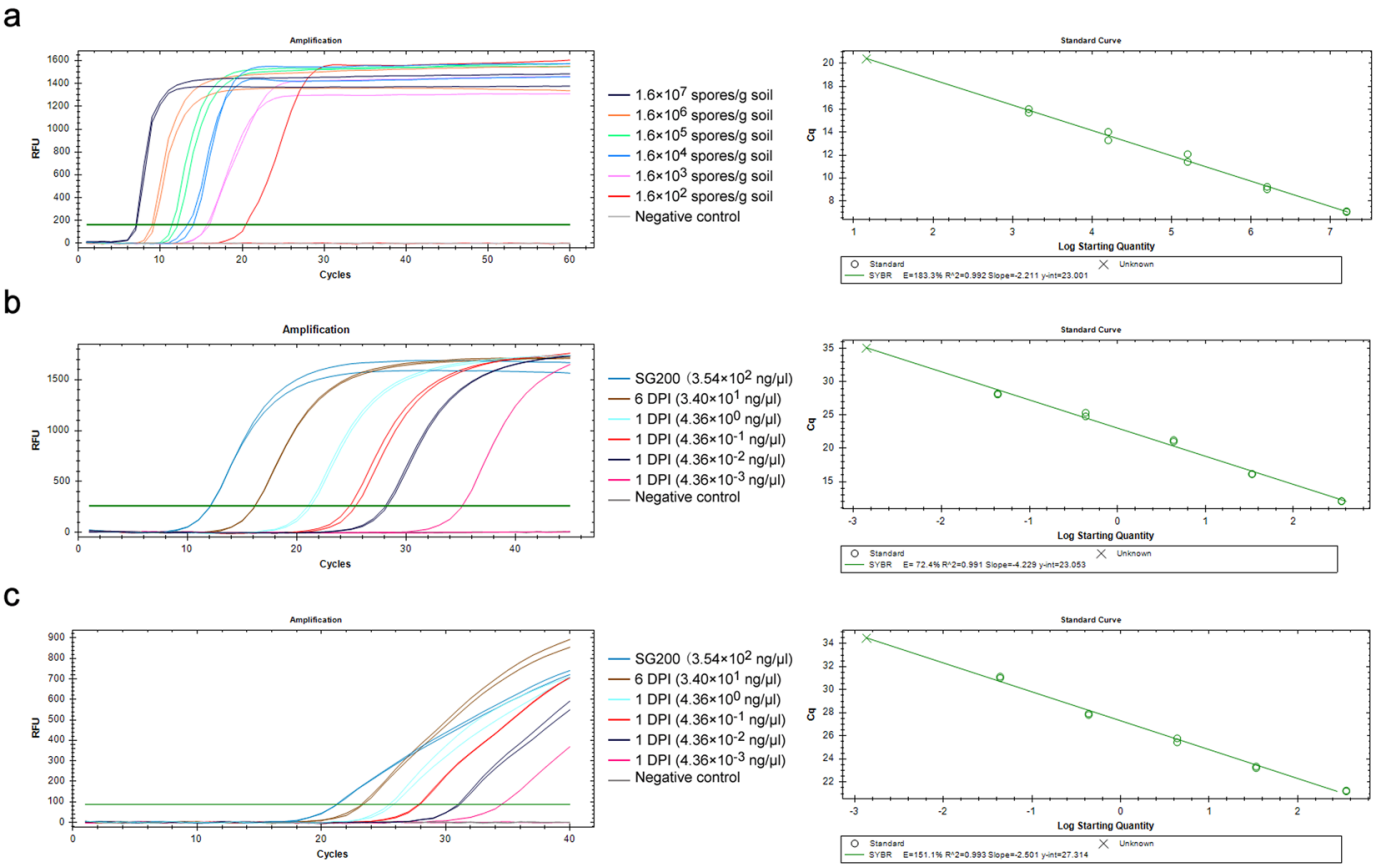
Quantitative detection of artificially inoculated soil and maize plant samples using RealAmp and real-time PCR. (**a**) Detection of *Ustilago maydis* from serial 10-fold dilutions ranging from 1.6 × 10^7^ to 1.6 × 10^2^ spores per gram of artificially infested soil using RealAmp. The quantitative detection of *U*. *maydis* in maize plants challenged with sporidia of *U*. *maydis* strain SG200 or compatible mating strains at 1 DPI (day post inoculation) and 6 DPI using RealAmp (**b**) and real-time PCR (**c**). Serially diluted template DNA (ranging from 10^0^ to 10^−3^) of 1 DPI were used to test the sensitivity of RealAmp in detecting *U*. *maydis* at the early stage of infection in the artificially inoculated maize plants. Approximately 354 ng/μl genomic DNA of *U*. *maydis* strain SG200 was employed as a quantitative reference.

Artificially infected soil and maize plant samples were prepared, and then DNA was extracted as described in the methods section. No *U. maydis* DNA was detected either in the uninoculated control soil samples with the RealAmp assay or with real-time PCR. The RealAmp assay could sensitively detect *U. maydis* contamination as low as 1.6 × 10^3^ teliospores/g of soil in artificially infested soil samples (Fig. 6a).

For the artificially inoculated maize samples, genomic DNA was extracted from maize plants that were injected with 10^8^ sporidia of *U*. *maydis* strain SG200 (or compatible mating strains) at 1 day and 6 days post inoculation (DPI), before the appearance of tumors. Approximately 354 ng/μl of SG200 genomic DNA was used as a quantitative reference. The data obtained indicated that the RealAmp can detect concentrations as low as 4.36 × 10^−2^ ng/μl *U*. *maydis* DNA at 1 DPI, and 34 ng/μl *U*. *maydis* was detected at 6 DPI by both RealAmp (Fig. 6b) and real-time PCR (Fig. 6c), respectively. The successful sensitivity test of the RealAmp assay in artificially inoculated samples demonstrated that it might be applicable for the detection of *U*. *maydis* in field samples either prior to or following infection.

### RealAmp detection in field samples

The feasibility of RealAmp for detecting artificially infested samples led us to attempt to quantitatively assay *U*. *maydis* in field conditions using this method.

**Figure 7:**
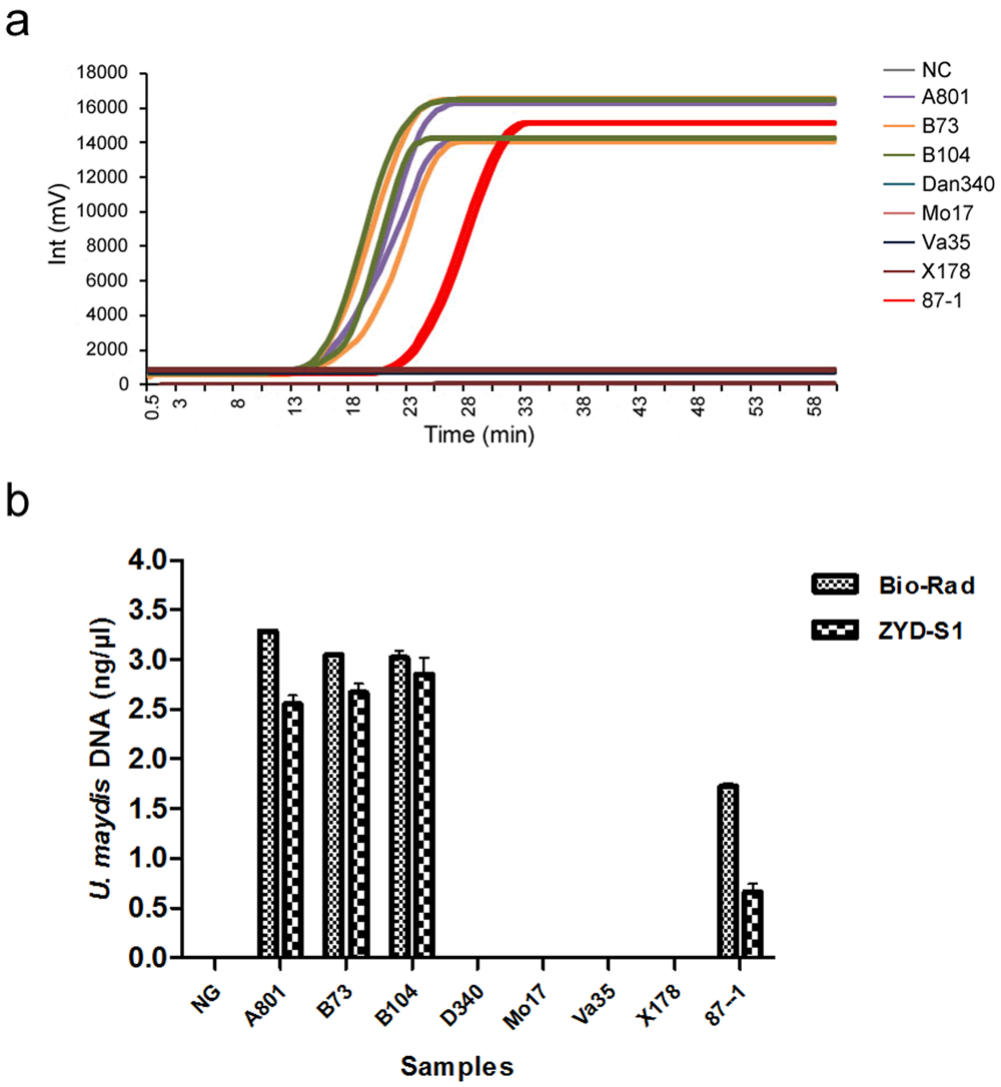
Detection of *Ustilago maydis* in plants from various maize inbred lines using RealAmp for disease tolerance testing. (**a**) The amplification curve graph reporting the fluorescence in mV on the *y*-axis over time in min on the *x*-axis was plotted by the ZYD-S1 tube scanner. (**b**) Comparison of the RealAmp quantitative results for samples from different maize inbred lines between using the Bio-Rad CFX96 Real-time PCR system and the ZYD-S1 instrument for fluorescence detection. Data are presented as the mean ( ± SD) of three replicates. The SPSS (version 20, SPSS Inc., Chicago, IL, USA) independent *t*-test software package was employed to compare the RealAmp quantitative results of different samples between using the Bio-Rad and ZYD-S1 instruments.

Initially, samples were collected from eight separate maize inbred lines at the big trumpet and tasseling stages, prior the appearance of tumor. These individuals were planted in a field infected with common smut for purposes of disease tolerance testing. The data from this experiment indicated that *U*. *maydis* could be detected in samples isolated from maize inbred lines A801, B73, B104 and 87-1, while the other four lines were indicated to be negative for the pathogen (Fig. 7). In addition, 150 soil and 72 maize plants samples were tested for the presence of *U. maydis* by RealAmp using the ZYD-S1 system. Results from the RealAmp assay showed that 190 of these samples tested positive, whereas 32 samples tested negative (Supplementary Table S2).

## Discussion

Common smut of corn caused by *U*. *maydis* is considered to be a troublesome disease throughout most of the regions where maize is produced. Sensitive and effective detection of *U*. *maydis* prior to or at an early stage of pathogen infection is indispensable for disease management and resistance breeding practices. As a diagnostic tool, microscopy has been used to analyze the infection events of *U*. *maydis* in maize plants^28,29^, However, this technique suffers from low sensitivity and is technically challenging when asymptomatic plants or individuals at an early stage of infection are analyzed. PCR-based assays provided a sensitive and reliable tool for the detection of *U*. *maydis* as previously described^6,7^, but this method can be time-consuming and is not convenient for use in field conditions.

In this work, we described the development of a rapid, sensitive, and quantitative RealAmp method for *U. maydis* detection using species-specific primers. To screen for optimal LAMP primers, three *U*. *maydis* effector genes (*Pep1*, *Pit2* and *See1*) were employed as targets for primer design. These virulence genes are involved in different events that occur during the *U*. *maydis* infection cycle^16–20^. Thus, the potential for successful infection of *U*. *maydis* on maize plants could be substantiated as the occurrence of the targeted gene was detected. Among the 12 sets of candidate LAMP primers, Pep-2, which presented the highest amplification efficiency and stability out of these sets, was selected for further development of our method (Fig. 1, Supplementary Fig. S1).

The Pep1 protein from *U*. *maydis* shows sequence identity ranging from 45.76% to 62.36% with its orthologs in six other smut fungi^30^. In order to enhance the specificity of the LAMP reaction, the variable region of this gene was targeted when designing candidate primers. In our study, the six LAMP primers of the Pep-2 set recognized the different variable regions of *UmPep1* (Supplementary Fig. S2), which helps to sufficiently distinguish the sequence in *U. maydis* from those of other maize pathogens such as *S. reilianum*, the cause of symptoms of head smut in maize plants (Fig. 4). Previous research indicated that there exist two clades of *U*. *maydis* based on the sequence of *Pep1* gene, most of the *U*. *maydis* belong to clade A, the *Pep1* sequences of different strains or isolates in the same clade is conserved^31^. The coding sequence of *Pep1* from clade B is identical to that of clade A except a 15 bp insertion at the position of nt 432 (Supplementary Fig. S7). The LAMP primer set Pep-2 used in this study were designed based on the *Pep1* coding sequence from clade A (XM_011389599), the primer B1c of Pep-2 that targeting nt 422 to 439 of XM_011389599 was designed to separate the strains of clade A from the strains of clade B (Supplementary Figs S7 and S8). To detect the strains from clade B, seven sets of LAMP primers (mPep-1 to mPep-7) were also designed (Supplementary Table S1), in which primer set mPep-2 was thought to be capable of amplifying *Pep1* segment specifically (Supplementary Figs S8 and S9). In a recent study, the *Pep1* gene was also selected as an amplification target for detection of *S*. *scitamineum* using LAMP in sugarcane^13^. *S*. *scitamineum Pep1* shares 63.7% and 73.33% nucleotide sequence identity with its orthologs in *U*. *maydis* and *S*. *reilianum*, respectively. Varying, non-conserved regions of the sequence were chosen for primer design in both studies; we selected more variable sequences in order to avoid the conserved central region^13^, (Supplementary Fig. S2). The use of loop primers further enhanced the specificity of the LAMP reaction as they targeted two separate variable regions of the target sequences (Supplementary Fig. S2). From what has been discussed above, we may speculate that the Pep-2 primer set is species-specific to *U. maydis* and to the exclusion of *S*. *scitamineum* and other smut pathogen as the specificity of the RealAmp primers was experimentally tested using several other pathogens.

Different parameters of the LAMP protocol could be adjusted in order to optimize the reaction. The ratio between inner and outer primers and the quantity of *Bst* DNA polymerase used in reaction are two critical variables for optimization^21^. In this work, we showed that as the concentration of *Bst* DNA polymerase was increased from 4 U to 8 U, the amplification improved dramatically, however, the tendency of amplification efficiency improvement from 6 U to 8 U is far less than that of 4 U to 6 U (Figs 2 and 3). 8 U of *Bst* DNA polymerase produced the best LAMP results under the optimal ratio of inner to outer primers and MgSO_4_ concentration (Figs 2 and 3). We speculated that the improvement of efficiency reach a plateau when the *Bst* enzyme is set at 8 U. This is consistent with previous reports^13,32^. The initial study evaluating LAMP primer ratios demonstrated that the concentration of the outer primer should be 1/4-1/10 that of the inner primer^21,32^. In our study, an optimal ratio (8:1) of the FIP/BIP to F3/B3 primers successfully avoided false positive results and low-efficiency amplification (Fig. 2). Mg^2+^ ions are also known to affect both DNA polymerase activity and PCR specificity^33^. An extremely high concentration may lead to false positives because of nonspecific amplification. At least 4 mM Mg^2+^ is needed for a noticeable reaction to occur and 6 to 8 mM results in an optimal LAMP reaction^32^. The optimal concentration of Mg^2+^ that has been reported recently for several LAMP methods is 8 mM^26,27,34–36^, which is higher than that for conventional PCR. This is because SYBR Green I was used in these reaction system, as previous reports have demonstrated that Mg^2+^ concentration should be at least 0.5–3 mM higher when used for florescent PCR than when used for conventional PCR according to the SYBR Green I product manual^34^. In the orthogonal experiment outlined in this report, combinations 1–4 (which employed 5 mM Mg^2+^) showed low efficiency of amplification even when the *Bst* DNA polymerase and primers ratio were under optimized conditions (Fig. 3). As the concentration of MgSO_4_ in the buffer for *Bst* DNA polymerase was increased from 5 mM to 8 mM, the amplification efficiency also increased, reaching its highest level at 8 mM (Fig. 2). These patterns were also observed in previously described studies^32,37^. However, this differs from the recently reported LAMP method employing SYBR Green I for detecting the sugarcane smut pathogen *S*. *scitamineum*, in which 6.00 mM was claimed to be the optimal concentration of Mg^2+^ ions^13^. The difference might be caused by the differing amounts of dNTPs as well as the templates and the primers used in the reactions^32,37^. Furthermore, we cannot rule out the possibility that the use of loop primers influences the Mg^2+^ optimization. In most cases, the optimization of LAMP is accomplished using single variable experiments, by which different individual parameters of the reaction are altered to screen for approximately optimal concentration ranges of a single reaction component while all other variables are held constant. Nevertheless, it is arduous to obtain the optimal experimental results due to the inobservance of potential interactions between these factors^13^. To overcome this problem, single factor experiments were carried out in combination with an orthogonal experimental design, which allowed the effects of interactions between the different reaction parameters to be determined so that their optimization with respect to the LAMP assay for detection of *U. maydis* could be established^13^. In this study, the orthogonal arrangement was designed based upon the results of single factor experiments, and the latter were further authenticated by the orthogonal experiment (Table 1, Figs 2 and 3). Thus an optimized LAMP system that was suitable for the detecting of *U*. *maydis* was successfully developed by adopting a single factor experiment followed by enhanced evaluation through the orthogonal experiment schedule.

The proposed RealAmp assay could detect as low as 44 fg/μl *U. maydis* plasmid DNA among whole DNA extracted from soil (Fig. 5, Supplementary Fig. S5), which was almost 200 times higher than the previously described conventional PCR-based method^6^. The real-time PCR method that was evaluated showed 10 times more sensitivity than the RealAmp assay when tested with *U. maydis* genomic DNA (Fig. 5, Supplementary Fig. S5). However, when using the plasmid DNA mixed with maize genomic DNA the RealAmp assay had approximately the same detection limit as real-time PCR (Supplementary Fig. S4). The lower detection sensitivity of RealAmp utilizing a plasmid template that is contained in a mixed DNA isolated form soil sample might be caused by potential inhibitory substances in the soil that constrain the reaction efficiency. In some circumstances, infection by *U*. *maydis* does not lead to the development of any external or characteristic symptoms of common smut. Thus determination of the RealAmp detection limit using artificially infested samples, including the teliospore-infested soil and maize plants inoculated with sporidia should assist in future studies on plant-pathogen interactions, smut disease control, and resistance breeding. The detection limit of soil samples artificially infested with teliospores was shown to be 1.6 × 10^3^ spores/g of soil (Fig. 6), which is equal to 8.0 × 10^−4^ ng/μl pEasy-Pep plasmid DNA according to the quantity of DNA extracted from the soil sample. This is 20 times lower than that calculated when using plasmid DNA diluted with DNA isolated from soil. This might be attributable to low yield encountered during preparation of teliospore nucleic acids from artificially inoculated soil owing to the thick wall of teliospores being somewhat resistant to lysis, and to co-purification inhibitors present in the sample. For the artificially inoculated maize plants samples, the RealAmp assay can detect as low as 4.36 × 10^−2^ ng/μl *U*. *maydis* genomic DNA at the earliest stage of infection prior to the appearance of symptoms. The sensitivity assessment using artificially infested samples suggested that RealAmp could be feasible for quantifying presence *U. maydis* in field samples. In this work, we were intrigued by the failure of RealAmp to amplify high-concentration template samples. In other words, no amplification was observed in reaction system using undiluted plasmid (440 ng/μl) in the preliminary experiment (Supplementary Fig. S4). We speculate that this is can be explained by the inability of the primers to anneal to their target sequences in the presence of large concentrations of template.

The portable fluorescent reader ZYD-S1^TM^, equipped with a battery pack, was used for detecting the presence of a plant pathogen for the first time in this study. This instrument has twice the tube holding capacity and twice the efficiency of detecting fluorescent signals than a similar tube scanner that has been previously described^26^. By means of the mobile DNA workstation, the ZYD-S1^TM^ provides a major advancement towards “electricity-free” LAMP technology, from template preparation to amplification and product quantitative detection, for evaluating field samples (Fig. 7, Supplementary Table S1). The RealAmp assay we developed here could become the foundation for integrated disease management practices that could guide maize planting to avoid further dissemination of *U*. *maydis*. It also might be a useful tool for research on plant-pathogen interactions and disease resistance breeding.

## Methods

### Pathogens and plant materials preparation

*U*. *maydis* haploid isolates A1~A3 (clade A) and D1~D3 (clade B) were generously donated by Dr. Qingchang Meng at Jiangsu Academy of Agricultural Sciences. The solopathogenic strain SG200 was kindly provided by Professor Doehlemann at the Max Planck Institute for Terrestrial Microbiology, Marburg, Germany. *U*. *maydis* isolates and strains used in this study were maintained on potato dextrose agar (PDA) medium.

The artificial inoculation of *U*. *maydis* on maize plants was performed as previously described^38^ using compatible mating strains or SG200. The infected maize samples were harvested prior to tumor formation for further DNA preparation.

### Soil samples preparation

Teliospores were prepared as previously described^39^. The harvested teliospores were adjusted to the desired concentration for soil inoculation (10^9^ spores/ml) by counting in a hemocytometer. For the artificially infected soil samples, 1 ml titers of the *U*. *maydis* teliospore suspension (~1.6 × 10^8^ spores) were inoculated onto 10 g of the twice-autoclaved soil substrate in 15 ml falcon tubes. The fungi were cultured at 25 °C for 10 d. Samples were then air-dried at ambient temperature (~3 d) and subsequently ground in liquid nitrogen to produce a fine powder, which was stored at −80 °C prior to DNA extraction.

### DNA extraction

The haploid strains of *U*. *maydis* and *S*. *reiliana* were grown in YEPS_L_ medium [containing 0.4% (w/v) yeast extracts (BD, Franklin Lakes, NJ, USA), 0.4% (w/v) peptone (BD) 2% (w/v) sucrose (Fishier Sci., Pittsburgh, PA, USA)] at 28 °C with shaking at 200 rpm to an OD_600_ of 0.8. Cultures were centrifuged at 900 x g for 5 min to pellet cells. Cells were then harvested for DNA extraction using the Mag-Bind^®^ universal 96 Kit (Omega Bio-Tek, Norcross, GA, USA) according to the manufacturer’s instructions, or using a modified version of the CTAB method as described by Xu *et al*.^6^.

Mycelial DNA of fungal species other than *U*. *maydis* or *S*. *reiliana* was extracted with the E.Z.N.A.^®^ SP Fungal DNA Kit (Omega Bio-Tek) following the manufacturer’s manual. The genomic DNA of maize plants was extracted with the E.Z.N.A.^®^ SP Plant DNA Midi Kit (Omega Bio-Tek). Genomic DNA was extracted from the soil samples (0.5 g, with approximately 10^8^ teliospores) as previously described^40,41^.

The soil and plants samples that isolated in field were treated and then subjected to DNA preparation in the mobile DH-WSW^TM^ mini DNA workstation (Double Helix Tech. Inc.).

### Screening of candidate LAMP primers

To screen for the optimal target gene for the *U*. *maydis* LAMP assay, three fungal effector genes [*UmPep1* of clade A (XM_011389599)*, UmPep1* of clade B (HQ002865), *UmPit2* (XM_752429) and *UmSee1* (XM_011390277)] of *U*. *maydis* were employed as targets for LAMP primer design in this study.

For each gene, six LAMP primers [external primers F3 and B3; forward and backward internal primers (FIP and BIP, respectively); and loop primers, LoopF and LoopB] were designed according to the gene’s cDNA sequence using the PrimerExplorer V4 software (http://primerexplorer.jp/e/; Eiken Chemical Co., Ltd., Tokyo, Japan). Four to seven sets of LAMP primers were designed for each of the three genes being evaluated. The sequences of these primers are listed in the Supplementary Table S1.

All primers were synthesized by Sango BioTech (Shanghai, China) at HPLC purification grade. The specificity of each primer was checked using the basic local alignment search tool (BLAST) against human DNA and other fungal sequences in the non-redundant GenBank database.

### Optimization of real-time LAMP reaction system

Real-time LAMP was conducted using methods described previously for screening *U*. *maydis* LAMP primers^26,27^. In order to optimize the LAMP reaction, a single factor experiment was conducted using three variables and four levels. For a fixed outer primer concentration of 0.2 μM, the concentration ratio of two primer pairs (outer primer F3/B3 and inner primer FIB/BIP) was adjusted to 1:2, 1:4, 1:6 and 1:8. The quantity of *Bst* DNA polymerase in the reaction mixture was set at 2.0 U, 4.0 U, 6.0 U and 8.0 U. Mg^2+^ concentrations were set to 5.0 mM, 6.0 mM, 7.0 mM and 8.0 mM, respectively (Fig. 2). All tests were repeated three times. In total, 16 combinations were studied sequentially in the LAMP optimization experiment employing L_16_ (4^5^) orthogonal experimental design to avoid potential interference caused by interactions between different factors of the LAMP reaction^42^, (Table 1). Each combination was repeated twice.

The RealAmp reaction was carried out on the CFX96^TM^ real-time PCR detection system (Bio-Rad, Hercules, CA, USA). The following reaction was repeated for 60 cycles: 63 °C for 30 s (stage 1), 63 °C for 15 s (stage 2), and 63 °C for 45 s (stage 3), followed by a melt curve (95 °C for 15 s, 65 °C to 95 °C increment 0.5 °C for 0.5 s). This reaction was coupled with detection of fluorescence signals after each cycle, which was analyzed by Bio-Rad CFX Manager 3.1. For all three reaction monitoring systems, a result was considered positive when an “S” amplification curve was obtained, while a linear or slightly oblique amplification curve indicated a negative result.

Alternatively, the RealAmp assay was performed at 63 °C for 60 min with pathogen detection instrument ZYD-S1^TM^ (Double Helix Tech. Inc.), which is a small, easy-to-use fluorescence measurement system, that has heating blocks capable of holding sixteen tubes, adjustable temperature settings, and spectral devices to detect amplified products using a fluorescent dye. The tube scanner was set to collect fluorescence signals at 30 s intervals. The threshold validation test was used to verify that the signal had sufficiently increased to be considered indicative of a positive result. During real-time amplification, the fluorescence data was obtained on the 6-carboxyfluorescein channel (excitation at 470 nm and detection at 520 nm), and a fluorescence unit threshold value was used. The threshold time (*Tt*) was calculated as the time after initiation of the reaction when the fluorescence signal reached the threshold value. The threshold value was the sum of the mean fluorescence signal during the initial 5 min and 10 times the standard deviation values during the same time-frame. In the resulting plot, the y-axis denoted the fluorescence units in mV. The x-axis shows the time in min.

### LAMP specificity

To confirm the specificity of the LAMP assay to *U. maydis*, DNA samples from other fungi including *Sporisorium reiliana, Curvularia lunata, Bipolaris maydis, Fusarium graminearum, F. moniliforme, Pythium inflatum* and *Colletotrichum truncatum* were employed in the assays. Additionally, the pathogenic bacteria *Salmonella enterica* and *Staphylococcus aureus* were used in the LAMP reactions as positive and negative controls, respectively.

### LAMP sensitivity

To determine the sensitivity of the *U*. *maydis* LAMP assay, a DNA fragment (190 bp) containing the LAMP target region of the gene of the *U*. *maydis* genome was amplified by conventional PCR with the primers UmPepF (5′-TCGTGTACCAATGCCAAAG-3′) and UmPepR (5′-TACCGATTCCTCCTAGCAG-3′). The obtained PCR product was purified and cloned into the pGEM^®^ -T Easy vector (Promega) according to the manufacturer’s instructions. The resulting recombinant plasmid, which was confirmed by sequencing (Supplementary Fig S6) and was designated as pEasy-Pep, was adjusted to a concentration of 440 ng/μl and diluted with a 10-fold serial dilution (1 × 10° to 1 × 10^7^ copies) before mixing with extracted DNA from soil or maize plant genomic samples. This recombinant plasmid was used as a reference to assess the detection limit of the LAMP assay.

### Real-time fluorescence PCR

The real-time PCR assay was performed thrice for each sample using the SYBR^®^ Premix ExTaq^TM^ kit (TaKaRa). Each PCR amplification procedure was performed with the CFX96^TM^ system (Bio-Rad) at a total volume of 25 μl, following the manufacturer’s instructions. The *U*. *maydis* Pep1-specific primer set of Pep-qF (5′-ACAATTCGTACACACTGCCG-3′) and Pep-qR (5′-CCCTTCTTCTCCTGGTCGTT-3′) was used, which was designed with the Primer3 web version 4.0 (http://primer3.ut.ee/)^43^. The thermal cycling conditions were set up as previously described^27^.

### Feasibility test of RealAmp

To evaluate the feasibility of the RealAmp method for the diagnosis of samples collected in the field, a total of 230 soil or maize plant samples were collected from established maize fields or fields that had never been planted with maize previously. The presence of *U*. *maydis* was detected with RealAmp using the ZY1-S1^TM^ pathogen detection instrument.

The quantification of *U. maydis* DNA in field samples operating on ZYD-S1^TM^ was analyzed statistically by calculating the corresponding threshold time value corrected with the standard curve formula that was determined in the RealAmp detection sensitivity experiment of artificially inoculated samples outlined above. To verify the RealAmp results of filed samples, the LAMP products were amplified using primers pair F2/B2 or mF2-2/mB2-2 and then were subjected to sequencing, in addition, the sporidia isolated from the above mentioned samples were propagated and which DNA templates were amplified using previously described primers Rok317/Rok318^31^ for sequencing validation.

## Electronic supplementary material

Supplementary Figures

Supplementary Table S1

Supplementary Table S2

## References

[1] Zhang CM, Liu YJ, Shi J, Zhang LZ (2005). Inoculation technique for identification of corn resistance to common smut. Maize Sci..

[2] Banuett F, Herskowitz I (1994). Morphological transitions in the life cycle of *Ustilago maydis* and their genetic control by the *a* and *b* loci. Exp. Mycol..

[3] Pataky, J. K. & Snetselaar, K.M. Common smut of corn. The Plant Heal. *Instructor*, 10.1094/PHI-I-2006-0927-01 (2006).

[4] Banuett F (1995). Genetics of *Ustilago maydis*, a fungal pathogen that induces tumors in maize. Annu. Rev. Genet..

[5] Brefort T (2009). *Ustilago maydis* as a pathogen. Annu. Rev. Phtyopathol..

[6] Xu ML, Melchinger AE, Lübberstedt T (1999). Species-specific detection of the maize pathogens *Sporisorium reiliana* and *Ustilago maydis* by dot blot hybridization and PCR-based assays. Plant Dis..

[7] Martínez-Espinoza AD, León-Ramírez CG (2003). Nisha Singh & José Ruiz-Herrera, J. Use of PCR to detect infection of differentially susceptible maize cultivars using *Ustilago maydis* strains of variable virulence. Int. Biol..

[8] Albert HH, Schenck S (1996). PCR amplification from a homolog of the bE mating-type gene as a sensitive assay for the presence of *Ustilago scitaminea* DNA. Plant Dis..

[9] Shen WK (2012). Development of a sensitive nested-polymerase chain reaction (PCR) assay for the detection of *Ustilago scitaminea*. Afr. J. Biotechnol..

[10] Willits DA, Sherwood JE (1999). Polymerase chain reaction detection of *Ustilago hordei* in leaves of susceptible and resistant barley varieties. Phytopathology.

[11] Su YC (2013). A Taqman real-time PCR assay for detection and quantification of *Sporisorium scitamineum* in sugarcane. The Scientific World J.

[12] Shen W, Xu G, Sun L, Zhang L, Jiang Z (2016). Development of a loop-mediated isothermal amplification assay for rapid and sensitive detection of *Sporisorium scitamineum* in sugarcane. Ann. Appl. Biol..

[13] Su Y (2016). Development and application of a rapid and visual loop-mediated isothermal amplification for the detection of *Sporisorium scitamineum* in sugarcane. Sci. Rep..

[14] Djamei A, Kahmann R (2012). *Ustilago maydis*: dissecting the molecular interface between pathogen and plant. PLoS Pathog..

[15] Ökmen B, Doehlemann G (2014). Inside plant: biotrophic strategies to modulate host immunity and metabolism. Curr. Opin. Plant Biol..

[16] Redkara A (2015). A secreted effector protein of *Ustilago maydis* guides maize leaf cells to form tumors. Plant Cell.

[17] Doehlemann G (2009). Pep1, a secreted effector protein of Ustilago maydis, is required for successful invasion of plant cells. PLoS Pathog..

[18] Hemetsberger C, Herrberger C, Zechmann B, Hillmer M, Doehlemann G (2012). The *Ustilago maydis* effector Pep1 suppresses plant immunity by inhibition of host peroxidase activity. PLoS Pathog..

[19] Doehlemann G, Reissmann S, Assmann D, Fleckenstein M, Kahmann R (2011). Two linked genes encoding a secreted effector and a membrane protein are essential for *Ustilago maydis* induced tumour formation. Mol. Microbiol..

[20] Mueller AN, Ziemann S, Treitschke S, Aßmann D, Doehlemann G (2013). Compatibility in the Ustilago maydis maize interaction requires inhibition of host cysteine proteases by the fungal effector Pit2. PLoS Pathog..

[21] Notomi T (2000). Loop-mediated isothermal amplification of DNA. Nucleic Acids Res..

[22] Nagamine K, Watanabe K, Ohtsuka K, Hase T, Notomi T (2001). Loop-mediated isothermal amplification reaction using a nondenatured template. Clin. Chem..

[23] Nagamine K, Hase T, Notomi T (2002). Accelerated reaction by loop-mediated isothermal amplification using loop primers. Mol. Cell. Probe..

[24] Lucchi NW (2010). Real-time fluorescence loop mediated isothermal amplification for the diagnosis of malaria. PLoS One.

[25] Njiru ZK, Yeboah-Manu D, Stinear TP, Fyfed JA (2012). Rapid and sensitive detection of *Mycobacterium ulcerans* by use of a loop-mediated isothermal amplification test. J. Clin. Microbiol..

[26] Zhang X (2014). Development of a real-time fluorescence loop-mediated isothermal amplification assay for rapid and quantitative detection of *Fusarium oxysporum* f. sp. *cubense* Tropical Race 4 in soil. PLoS One.

[27] Cao Y (2016). Rapid and quantitative detection of *Pythium inflatum* by real-time fluorescence loop-mediated isothermal amplification assay. Eur. J. Plant Pathol..

[28] Banuett F, Herskowitz I (1996). Discrete developmental stages during teliospore formation in the corn smut fungus. Ustilago maydis. Development.

[29] Callow JA, Ling IT (1973). Histology of neoplasms and chlorotic lesions in the maize seedlings following the infection of sporidia of *Ustilago maydis* (DC) Corda. Physiol. Plant Path..

[30] Hemetsberger C (2015). The fungal core effector Pep1 is conserved across smuts of dicots and monocots. New Phytol..

[31] Kellner, R., Hanschke, C. & Begerow D. Patterns of variation at Ustilago maydis virulence clusters 2A and 19A largely reflect the demographic history of its populations. *PLoS One* **9**, e988837; 10.1371/journal.pone.0098837 (2014). 10.1371/journal.pone.0098837 PMC4041787 24887029

[32] Nie X (2005). Reverse transcription loop-mediated isothermal amplification of DNA for detection of *Potato virus Y*. Plant Dis..

[33] Saiki RK (1988). Primer directed enzymatic amplification of DNA with a thermostable DNA polymerase. Science.

[34] Cao L, Cheng R, Yao L, Yuan S, Yao X (2014). Establishment and application of a loop-mediated isothermal amplification method for simple, specific, sensitive and rapid detection of *Toxoplasma gondii*. J. Vet. Med. Sci..

[35] Ma B, Dai M, Fang J, Wu Y, Zhang M (2016). Visual loop-mediated isothermal amplification (LAMP) method for identification bovine and ovine gene in animal foodstuff. Am. J. Food Technol..

[36] En FX, Wei X, Jian L, Qin C (2008). Loop-mediated isothermal amplification establishment for detection of pseudorabies virus. J. Virol. Methods.

[37] Tomita N, Mori Y, Kanda H, Notomi T (2008). Loop-mediated isothermal amplification (LAMP) of gene sequences and simple visual detection of products. Nat. Protoc..

[38] Redkar, A. & Doehlemann, G. *Ustilago maydis* virulence assays in maize. *Bio-protoc*. **6**, e1760; 10.21769/BioProtoc.1760 (2016).

[39] Sacadura NT, Saville BJ (2003). Gene expression and EST analyses of *Ustilago maydis* germinating teliospores. Fungal Genet. Biol..

[40] Volossiouk T, Robb EJ, Nazar RN (1995). Direct DNA extraction for PCR-mediated assays of soil organisms. Appl. Environ. Microbiol..

[41] Wang PH, Chang CW (2003). Detection of the low-germination-rate resting oospores of *Pythium miriotylum* from soil by PCR. Lett. Appl. Microbiol..

[42] Ming, D. X. Field test and statistical analysis (ed. Ming, D. X.) 294 (Science press, Beijing, 2005).

[43] Untergasser A (2012). Primer3 - new capabilities and interfaces. Nucleic Acids Res..

