# Supplementary material for: Development of a real-time fluorescence loop-mediated isothermal amplification assay for rapid and quantitative detection of Ustilago maydis
Source: Sci Rep. 2017 Oct 17;7:13394. doi: 10.1038/s41598-017-13881-4 (PMC5645423; doi:10.1038/s41598-017-13881-4)
Supplement: Supplementary file 1 — Supplementary Figures [file 41598_2017_13881_MOESM1_ESM.pdf]

TITLE:

Development of a real-time fluorescence loop-mediated isothermal amplification assay for rapid and quantitative detection of *Ustilago maydis*

AUTHORS:

Yanyong Cao[1, +], Lifeng Wang[1, +], Liping Duan[2], Jingjing Li[1], Juan Ma[1], Shuna Xie[3], Lei Shi[2] & Huiyong Li[1]

AFFILIATION:

[1] Institute of Cereal Crops, Henan Academy of Agricultural Sciences, Zhengzhou, 450002, China

[2] Institute of Food Safety and Nutrition, Jinan University, Guangzhou, 510632, China

[3] Institute of Plant Protection, Henan Academy of Agricultural Sciences, Zhengzhou, 450002, China

Correspondence and requests for materials should be addressed to Y.C. (email: yanyongcao@126.com) or H.L. (email: lihuiyong1977@126.com)
+These authors contribute equally to this work.

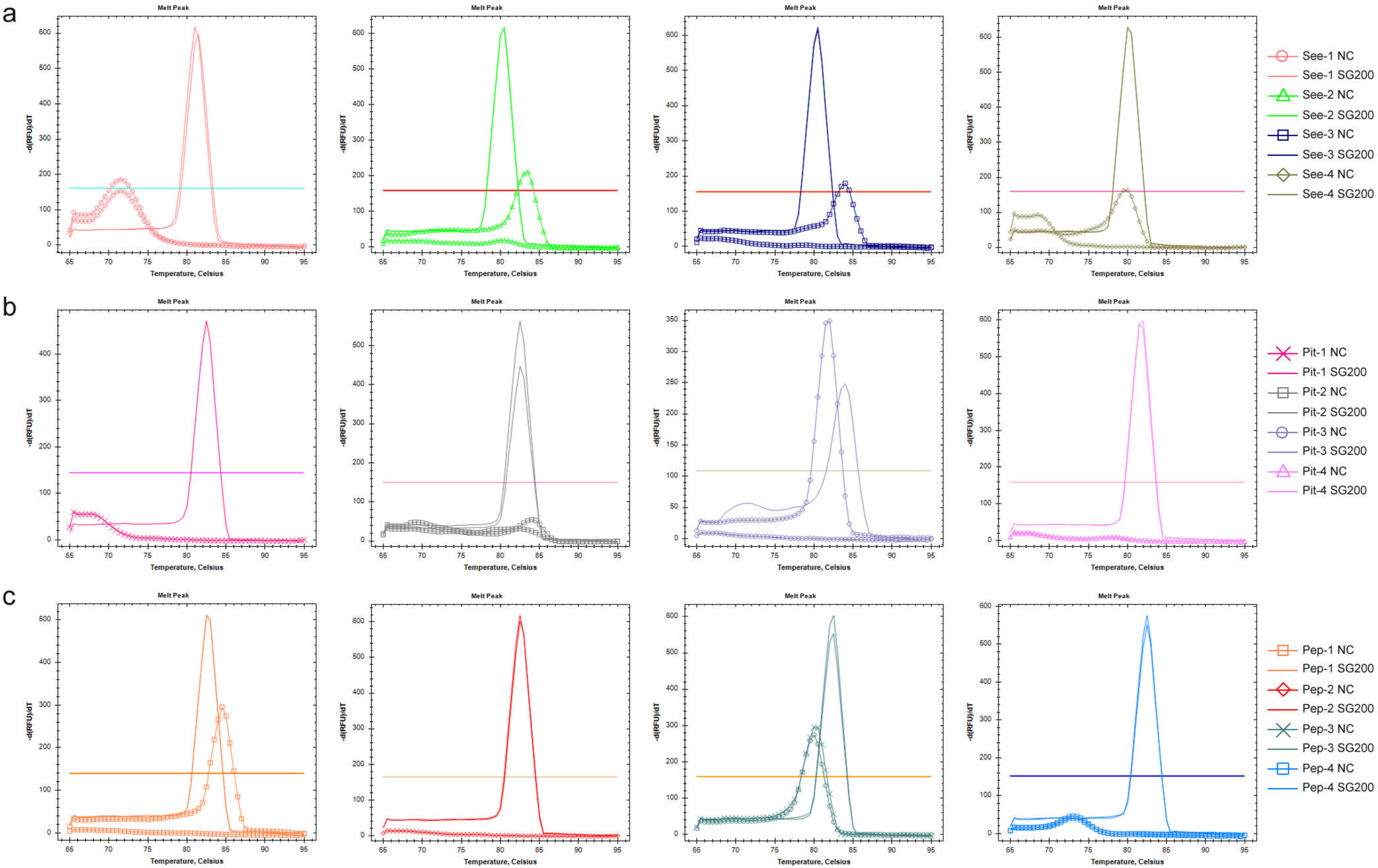

Supplementary Figure S1. Melt peaks of primer screening for *Ustilago maydis* LAMP reaction. 1–4 indicates one of the four sets of primers each designed for *See1*, *Pit2* and *Pep1*, respectively. NC indicates the negative control reactions.

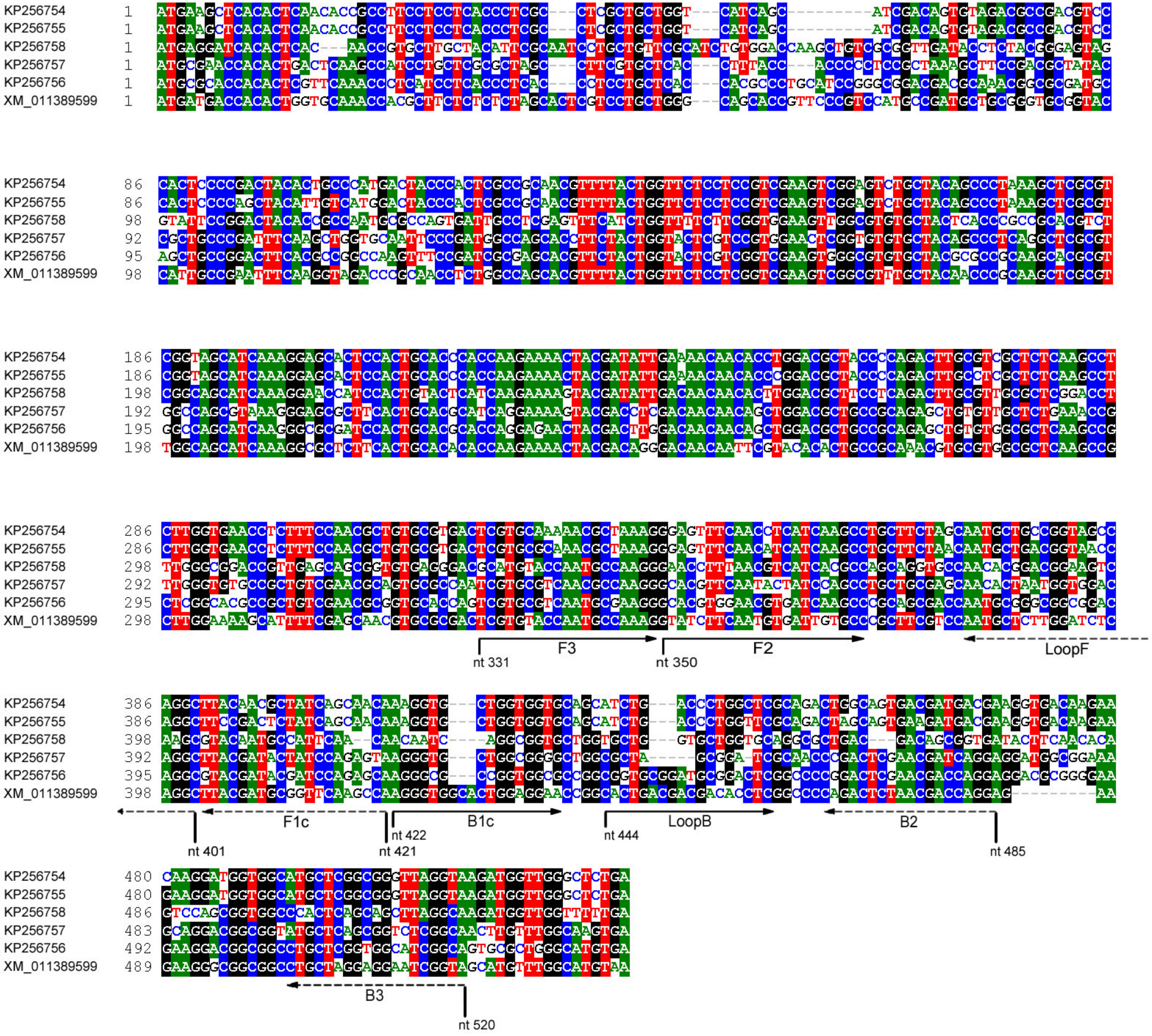

Supplementary Figure S2. The alignment of different *Pep1* orthologues and the specific sequences of *Ustilago maydis Pep1* LAMP primer set Pep- 2. The alignment was performed using BioEdit (v7.2.5, http://www.mbio.ncsu.edu/bioedit/bioedit.html). Sequences of *Pep1* genes analyzed in the alignment were obtained from GenBank. Accessions were as follows: *U. avenae Pep1*, KP256754; *U. nuda Pep1*, KP256755; *Sporisorium reilianum Pep1*, KP256756; *S. scitamineum Pep1*, KP256757; *Melanopsichium pennsylvanicum Pep1*, KP256758 and *U. maydis Pep1*, XM_011389599. The respective biding sites of Pep-2 primers are indicated by arrows.

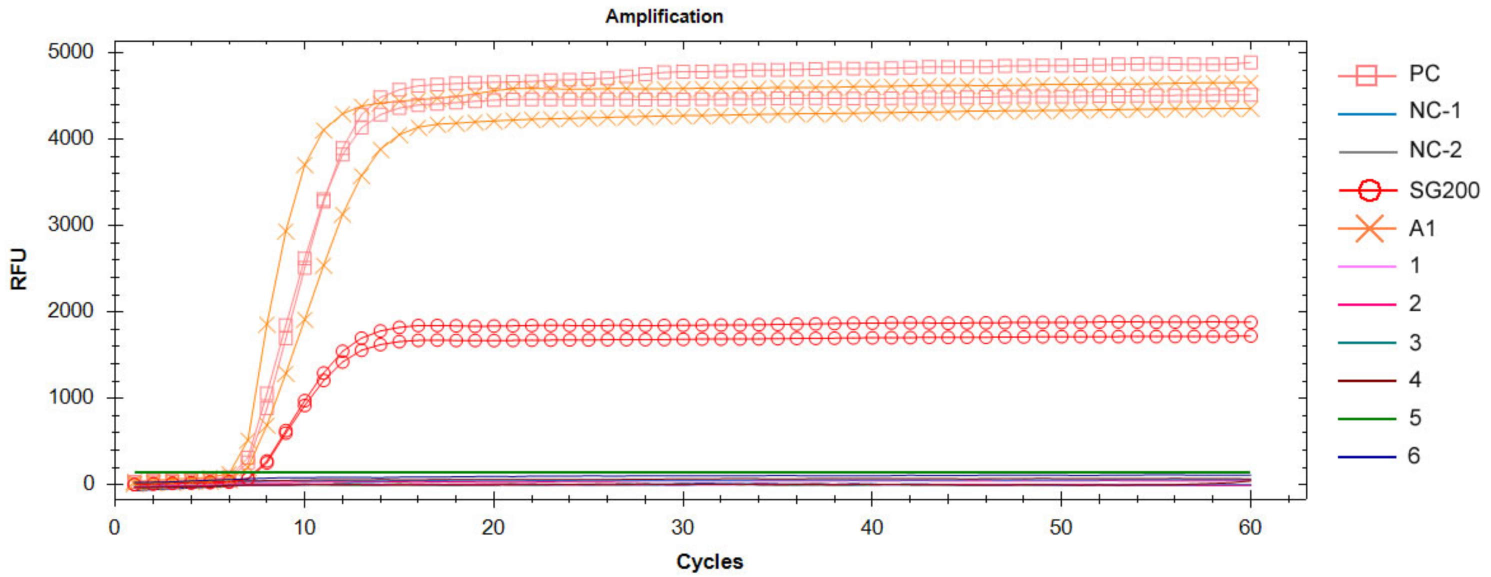

Supplementary Figure S3. Specificity test of RealAmp assay for the detection of *Ustilago maydis* using diluted templates. The RealAmp fluorescence (RFU) vs. Cq [threshold time (min)] amplification curve graph was plotted automatically by the Bio-Rad CFX96 Real-Time PCR system. The samples used are indicated as follows: PC, positive control (*Staphylococcus aureus*); NC-1, negative control 1 (*Salmonella choleraesusi*); NC-2, negative control 2 (*Salmonella bongori*); SG200, *U. maydis* strain SG-200; A1, *U. maydis* strain A1; 1 (*Curvularia lunata*), 2 (*Bipolaris maydis*), 3 (*Colletotrichum truncatum*), 4 (*Fusarium moniliforme*), 5 (*F. graminearum*), 6 (*Pythium inflatum*).

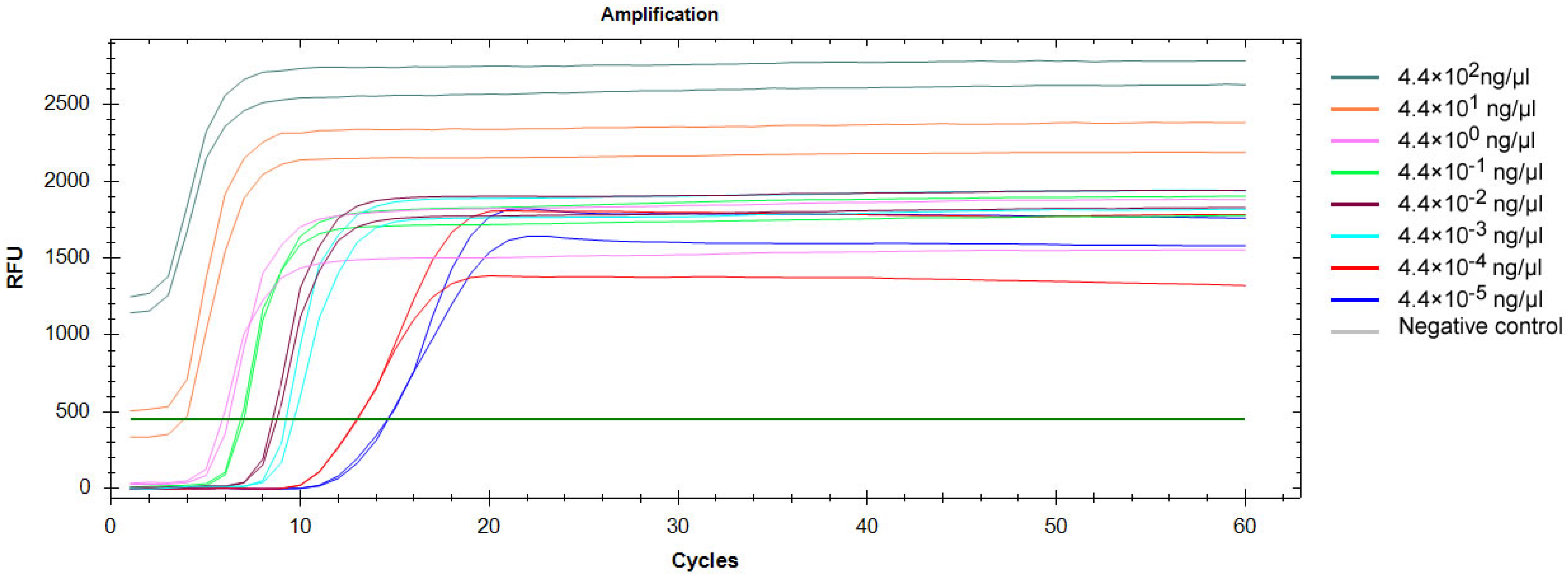

Supplementary Figure S4. Sensitivity test of *Ustilago maydis* RealAmp using extensive range of plasmid DNA. The Real-Time fluorescence units were plotted against the Log10-transformed initial concentration of plasmid DNA ranging from 4.4×10^{2} ng/µl to 4.4×10^{-5} ng/µl by the CFX96 Real-Time PCR system.

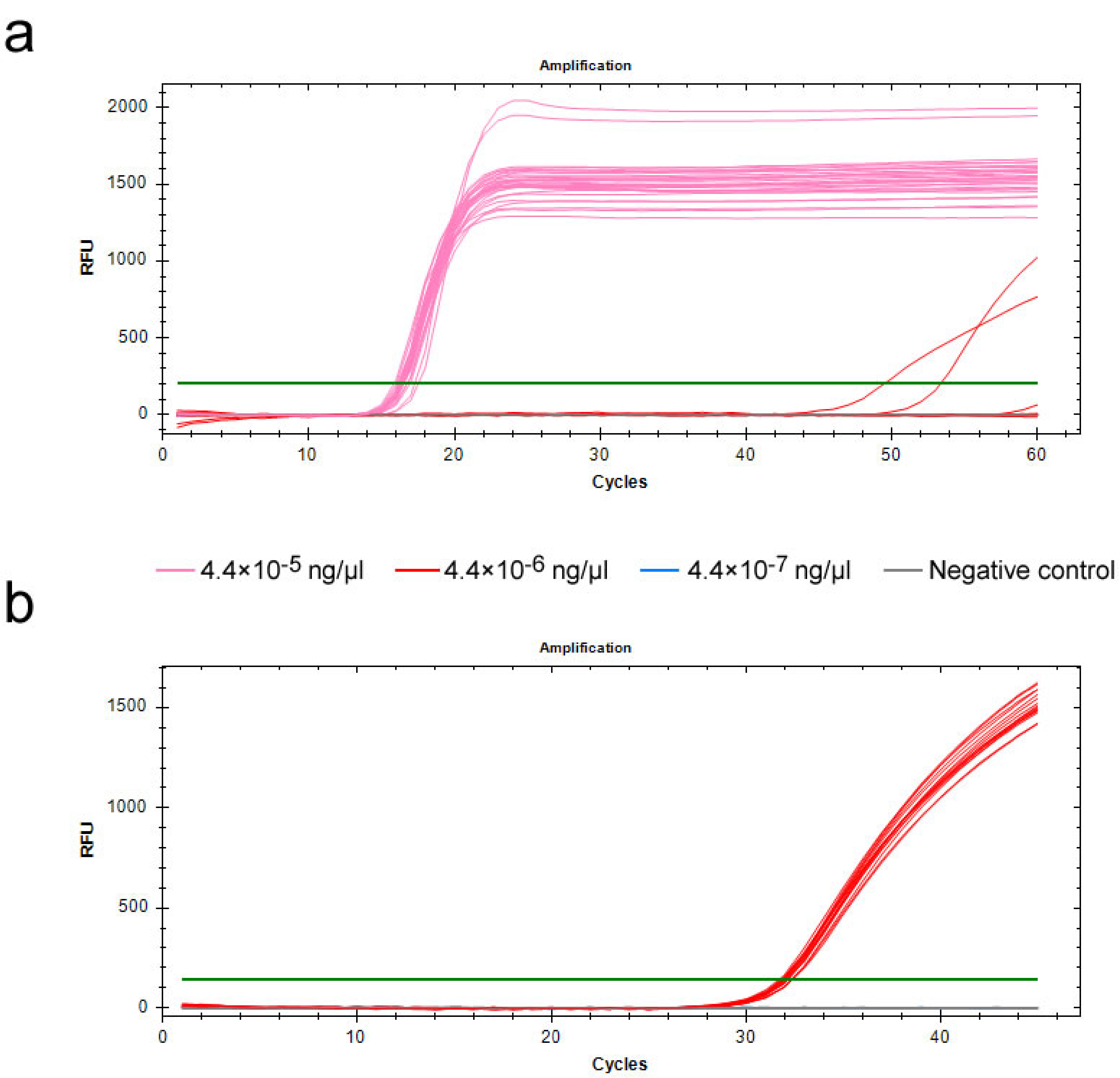

Supplementary Figure S5. Determination of the detection limit of RealAmp. The reactions were conducted by RealAmp (a) and real-time PCR (b) using $10^{-7}/10^{-8}$ and $10^{-8}/10^{-9}$ concentrations of the original plasmid template (4.4×10^{2} ng/μl). These reactions were repeated 30 times and 20 times, respectively.

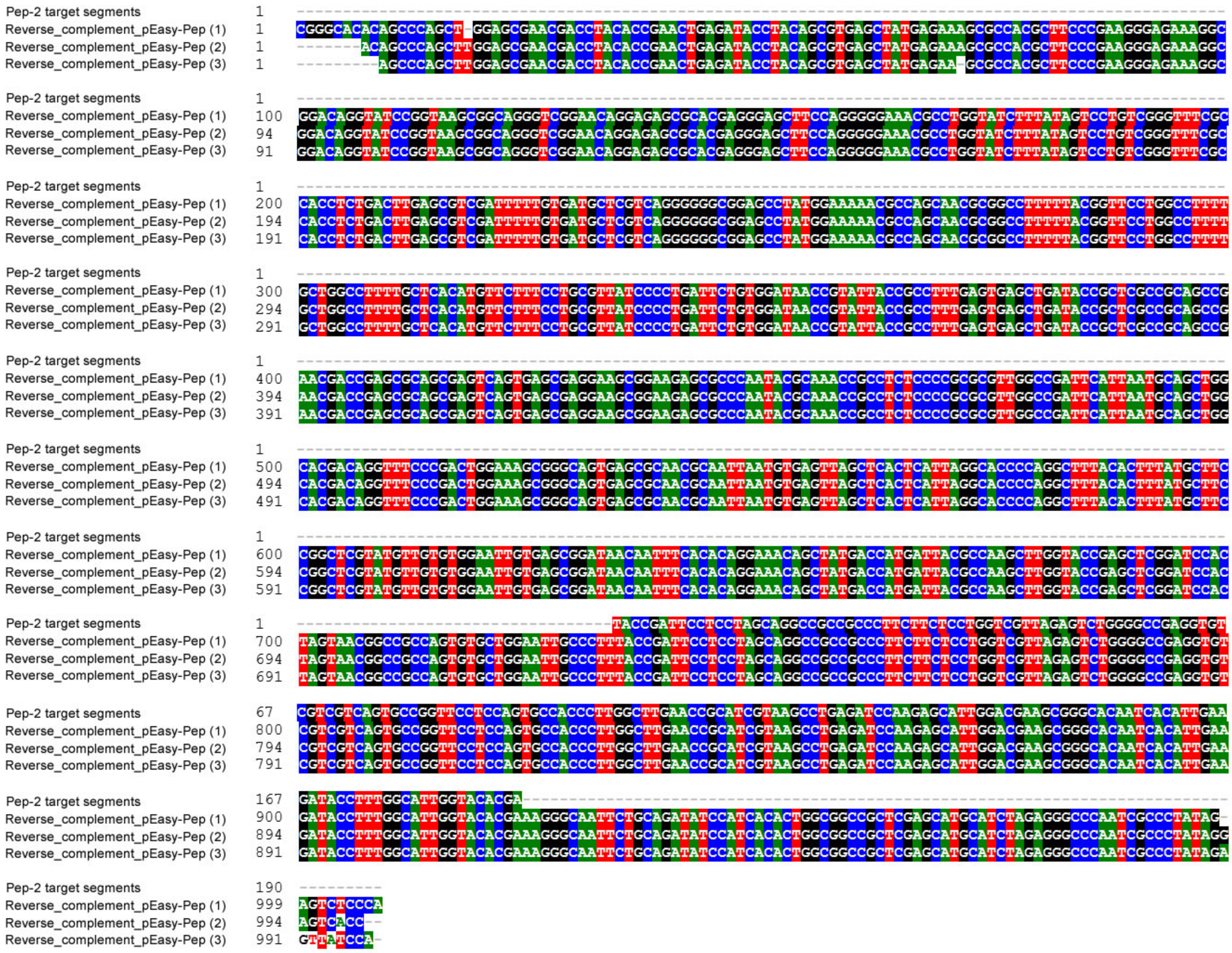

Supplementary Figure S6. The alignment of different pEasy-Pep plasmid sequence and the *Ustilago maydis* specific segment amplified from primer pair UmPepF /UmPepR. The alignment was performed using BioEdit (v7.2.5, http://www.mbio.ncsu.edu/bioedit/bioedit.html).

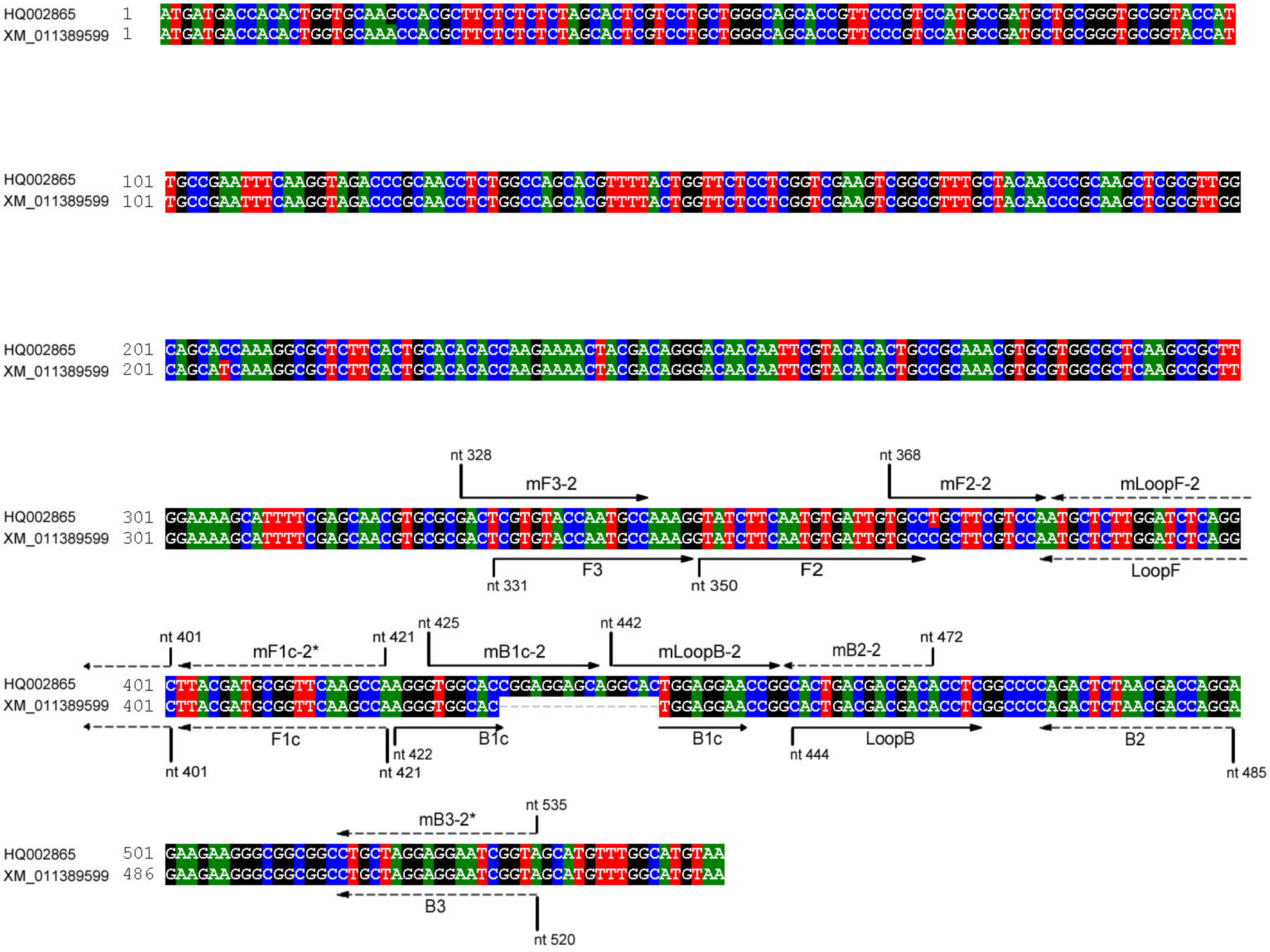

Supplementary Figure S7. The alignment of two clades of *Ustilago maydis Pep1* and the specific sequences of *Pep*1 RealAmp primer sets Pep-2 and mPep-2. Sequences of *Pep1* genes obtained from Clade A (XM_011389599) and Clade B (HQ002865) were analyzed in the alignment using BioEdit software (v7.2.5, http://www.mbio.ncsu.edu/bioedit/bioedit.html). The sequences and respective biding sites of Pep-2 and mPep-2 primers that targeting *Pep1* gene from clade A and clade B were indicated by arrows, respectively.

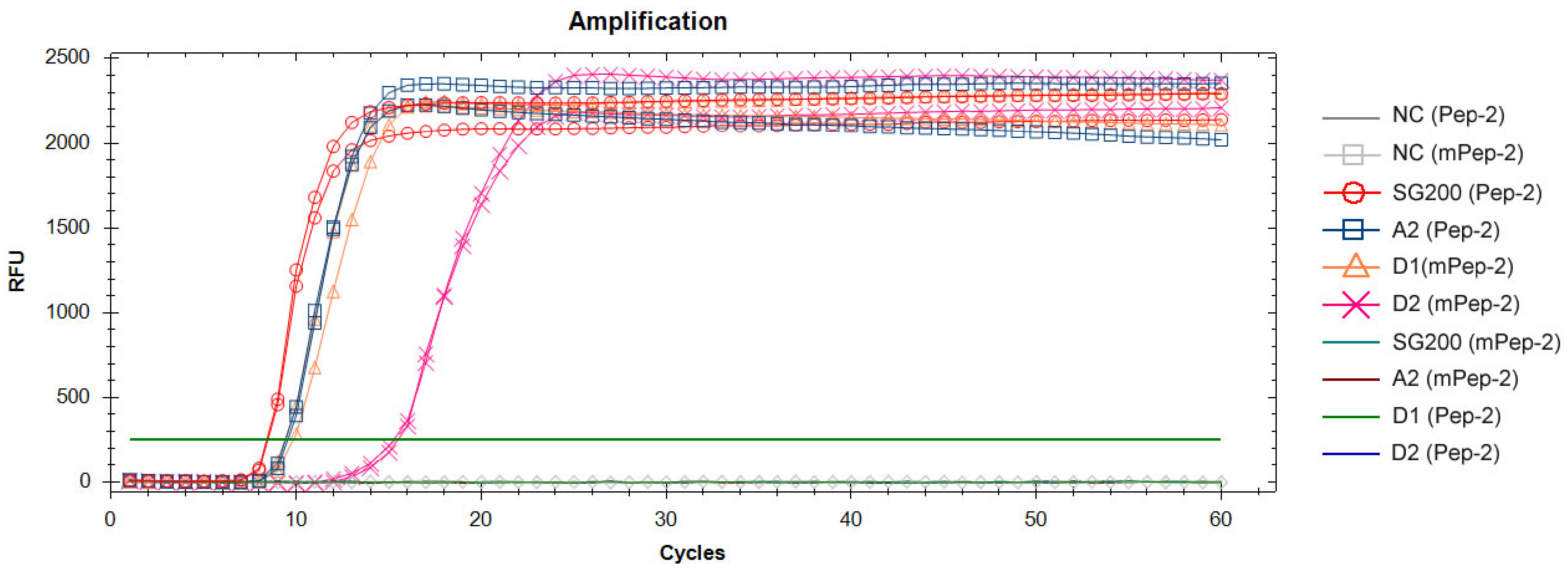

Supplementary Figure S8. Specificity test of RealAmp assay for the detection of two clades of *Ustilago maydis* using the LAMP primer sets Pep-2 and mPep-2. The RealAmp fluorescence (RFU) vs. Cq [threshold time (min)] amplification curve graph was plotted automatically by the Bio-Rad CFX96 Real-Time PCR system. The samples used are indicated as follows: NC, negative control (*Salmonella choleraesusi*), *U. maydis* clade A strains SG200 and A2, *U. maydis* clade B strains D1 and D2. For each sample, two sets of LAMP primer Pep-2 and mPep-2 that designed for the *Pep1* genes of clade A and clade B *U. maydis* were employed for the amplification, respectively.

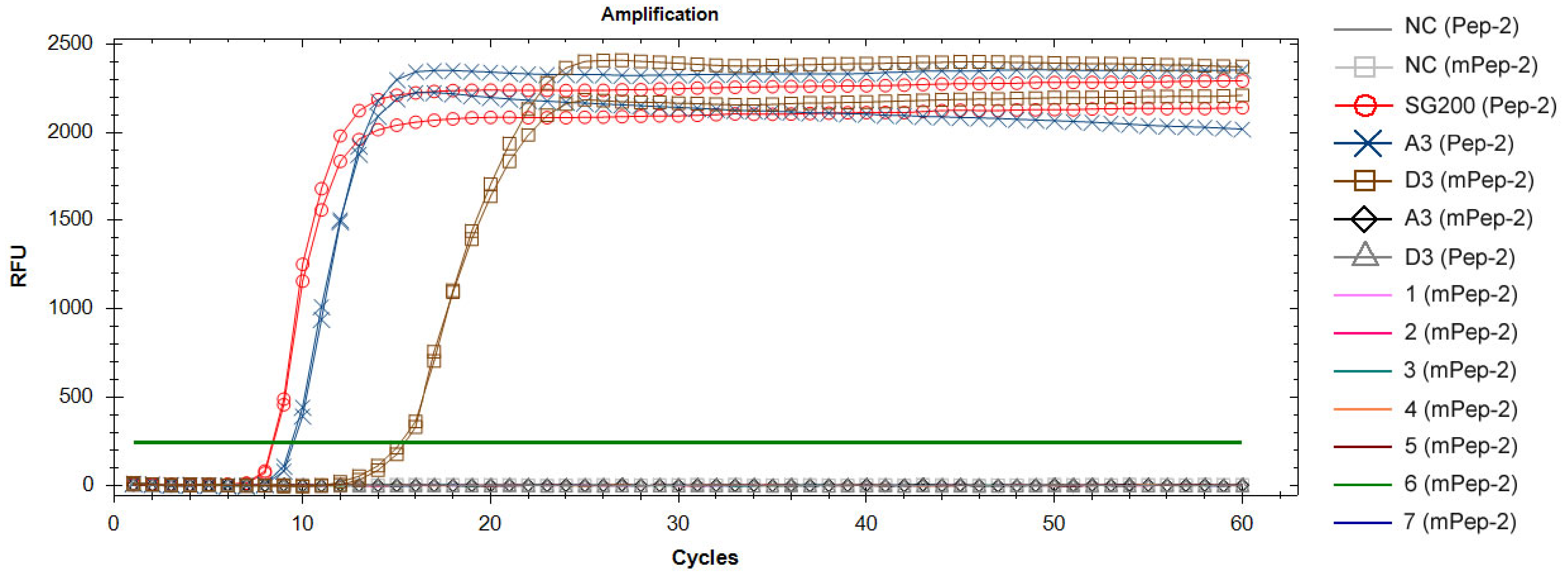

Supplementary Figure S9. Specificity test of RealAmp assay for the detection of *Ustilago maydis* using primer set mPep-2. The RealAmp fluorescence (RFU) vs. Cq [threshold time (min)] amplification curve graph was plotted automatically by the Bio-Rad CFX96 Real-Time PCR system. The templates consisted of genomic DNA isolated from the following control, two clades of *U. maydis* strains and other pathogens: NC, negative control (*Salmonella choleraesusi*); *U. maydis* clade A strains SG200 and A3, *U. maydis* clade B strain D3. 1, *Curvularia lunata*; 2, *Bipolaris maydis*; 3, *Colletotrichum truncatum*; 4, *Sporisorium reiliana*; 5, *Fusarium moniliforme*; 6, *F. graminearum*; 7, *Pythium inflatum*. For the NC, A3 and D3 templates, two sets of LAMP primer Pep-2 and mPep-2 that designed for the *Pep1* genes of clade A and clade B *U. maydis* were employed for the amplification, whereas for the other templates, the mPep-2 was solely used for the reaction.

Supplementary Table S1. The screening of different sets of primers used for *Ustilago maydis* real-time fluorescence loop-mediated isothermal amplification

Name*	Primer sequences (5' to 3')	Position	Length
See-F3-1	GCCATCAACAAGAATTCGC	265	19
See-B3-1	TCGGCCCAAATTTATACTCTC	466	21
See-FIP(F1c+F2)-1	CTGCCTGTCGTACGTGAACTCCACGGAGGACTACATAGACT		41
See-BIP(B1c+B2)-1	CTCGTACAGGTATGGCGATAGCCAGAAGCAGACTGGTGATC		41
See-LoopF-1	CCTTCTCCTTGCACCTGAG	332	19
See-LoopB-1	AAGCGGAGTATTCTGTTGCT	407	20
See-F2-1	CACGGAGGACTACATAGACT	289	20
See-F1c-1	CTGCCTGTCGTACGTGAACTC	354	21
See-B2-1	CAGAAGCAGACTGGTGATC	445	19
See-B1c-1	CTCGTACAGGTATGGCGATAGC	369	22
Product	CACGGAGGACTACATAGACTTCCGGCTCAGGTGCAAGGAGAAGGCGAGTTCACGTACGACAGGCAGAGAA ATGCAGTCGGCTCGTACAGGTATGGCGATAGCCATGGTAACAGTAGGGAAGCGGAGTATTCTGTTGCTGATC ACCAGTCTGCTTCTG		157
See-F3-2	CTTCACCACCTTCGTTTCA	6	19
See-B3-2	CGCTCGTGAATCTGTTCA	224	18
See-FIP(F1c+F2)-2	CAATGGCAGAGGACGAACGACTCCTCGTCATACTCTGTCT		40
See-BIP(B1c+B2)-2	GCAAGCAGAAGCACAAGATCAAGCTCGATGCTGTCTTCAG		40
See-LoopF-2	GTAGAGGATGAGCAGACACG	73	20
See-LoopB-2	GCAATTTGAAGAGGAGATCGC	129	21
See-F2-2	CTCCTCGTCATACTCTGTCT	28	20
See-F1c-2	CAATGGCAGAGGACGAACGA	100	20
See-B2-2	GCTCGATGCTGTCTTCAG	175	18

See-B1c-2	GCAAGCAGAAGCACAAGATCAA	101	22
Product	CTCCTCGTCATACTCTGTCTTGTCCACGTGTCTGCTCATCCTCTACAATCGTTTCGTTCGTCCTCTGCCATTG GCAAGCAGAAGCACAAGATCAAGTCTAGGCAATTTGAAGAGGAGATCGCGCAAGGTGCTGAAGACAGCATC GAGC		148
See-F3-3	CTCCTCGTCATACTCTGTCT	28	20
See-B3-3	GTTTTGTTGCTCTGTGCG	240	18
See-FIP(F1c+F2)-3	GATCTTGTGCTTCTGCTTGCCGTCTGCTCATCCTCTACAATC		42
See-BIP(B1c+B2)-3	ATCGCGCAAGGTGCTGAATTCAGACGAATCGTGAACC		37
See-LoopF-3	AATGGCAGAGGACGAACG	99	18
See-LoopB-3	CGAGCTGTTCGAGTTTCCT	171	19
See-F2-3	GTCTGCTCATCCTCTACAATC	57	21
See-F1c-3	GATCTTGTGCTTCTGCTTGCC	120	21
See-B2-3	TTCAGACGAATCGTGAACC	210	19
See-B1c-3	ATCGCGCAAGGTGCTGAA	145	18
Product	GTCTGCTCATCCTCTACAATCGTTTCGTTCGTCCTCTGCCATTGGCAAGCAGAAGCACAAGATCAAGTCTAGG CAATTTGAAGAGGAGATCGCGCAAGGTGCTGAAGACAGCATCGAGCTGTTCGAGTTTCCTCGGGTTCACGA TTCGTCTGAA		154
See-F3-4	CACCTTCGTTTCACTGCT	12	18
See-B3-4	GTTTTGTTGCTCTGTGCG	240	18
See-FIP(F1c+F2)-4	CAATGGCAGAGGACGAACGACATACTCTGTCTTGTCCACG		40
See-BIP(B1c+B2)-4	ATCGCGCAAGGTGCTGAATTCAGACGAATCGTGAACC		37
See-LoopF-4	AACGATTGTAGAGGATGAGCAG	80	22
See-LoopB-4	CGAGCTGTTCGAGTTTCCT	171	19
See-F2-4	CATACTCTGTCTTGTCCACG	36	20
See-F1c-4	CAATGGCAGAGGACGAACGA	100	20

See-B2-4	TTCAGACGAATCGTGAACC	210	19
See-B1c-4	ATCGCGCAAGGTGCTGAA	145	18
Product	CATACTCTGTCTTGTCCACGTGTCTGCTCATCCTCTACAATCGTTTCGTTCGTCCTCTGCCATTGGCAAGCAG AAGCACAAGATCAAGTCTAGGCAATTTGAAGAGGAGATCGCGCAAGGTGCTGAAGACAGCATCGAGCTGTT CGAGTTTCCTCGGGTTCACGATTCGTCTGAA		175
Pit-F3-1	TTGTTCTGCTCATCGTGG	20	18
Pit-B3-1	CATGACCACCGTCTTGAAT	318	19
Pit-FIP(F1c+F2)-1	GCGAACCTGTGAAGCCGAATCTCTACCGATGCCTCAAT		38
Pit-BIP(B1c+B2)-1	TCGGCAAGGAACCTGACAACGGCGGATTCTTGATGATGA		39
Pit-LoopF-1	CCACCATCTCCGGTTGAG	150	18
Pit-LoopB-1	GGCCAAGTACAGATCAAGATCA	190	22
Pit-F2-1	TCTCTACCGATGCCTCAAT	95	19
Pit-F1c-1	GCGAACCTGTGAAGCCGAA	169	19
Pit-B2-1	GGCGGATTCTTGATGATGA	242	19
Pit-B1c-1	TCGGCAAGGAACCTGACAAC	170	20
Product	TCTCTACCGATGCCTCAATGAGCTCGGCTGCTGGCAAGCTCAACCGGAGATGGTGGTTCGGCTTCACAGGT TCGCTCGGCAAGGAACCTGACAACGGCCAAGTACAGATCAAGATCATCCCAGACGCGCTCATCATCAAGAAT CCGCC		148
Pit-F3-2	TTGTTCTGCTCATCGTGG	20	18
Pit-B3-2	ACCACCGTCTTGAATCTTG	314	19
Pit-FIP(F1c+F2)-2	GCGAACCTGTGAAGCCGAATCTCTACCGATGCCTCAAT		38
Pit-BIP(B1c+B2)-2	TCGGCAAGGAACCTGACAACGGCGGATTCTTGATGATGA		39
Pit-LoopF-2	CCACCATCTCCGGTTGAG	150	18
Pit-LoopB-2	GGCCAAGTACAGATCAAGATCA	190	22

Pit-F2-2	TCTCTACCGATGCCTCAAT	95	19
Pit-F1c-2	GCGAACCTGTGAAGCCGAA	169	19
Pit-B2-2	GGCGGATTCTTGATGATGA	242	19
Pit-B1c-2	TCGGCAAGGAACCTGACAAC	170	20
Product	TCTCTACCGATGCCTCAATGAGCTCGGCTGCTGGCAAGCTCAACCGGAGATGGTGGTTCGGCTTCACAGGTTCGCTCGGCAAGGAACCTGACAACGGCCAAGTACAGATCAAGATCATCCCAGACGCGCTCATCATCAAGAATCCGCC		148
Pit-F3-3	TTTCGCTCAGCCTTTGTT	7	18
Pit-B3-3	GGCGGATTCTTGATGATGA	242	19
Pit-FIP(F1c+F2)-3	GTTGAGCTTGCCAGCAGCCAACATGTTCAAGCTATTCCG		39
Pit-BIP(B1c+B2)-3	GTGGTTCGGCTTCACAGGTTGATCTTGATCTGTACTTGGC		40
Pit-LoopF-3	GCTCATTGAGGCATCGGTA	117	19
Pit-LoopB-3	CGGCAAGGAACCTGACAA	171	18
Pit-F2-3	CAACATGTTCAAGCTATTCCG	61	21
Pit-F1c-3	GTTGAGCTTGCCAGCAGC	138	18
Pit-B2-3	TGATCTTGATCTGTACTTGGC	211	21
Pit-B1c-3	GTGGTTCGGCTTCACAGGT	147	19
Product	CAACATGTTCAAGCTATTCCGGTGCGTCGATCGCTCTCTACCGATGCCTCAATGAGCTCGGCTGCTGGCAAGCTCAACCGGAGATGGTGGTTCGGCTTCACAGGTTCGCTCGGCAAGGAACCTGACAACGGCCAAGTACAGATCAAGATCA		151
Pit-F3-4	TCTCTACCGATGCCTCAA	95	18
Pit-B3-4	TTAGGATCTGTCGGCATGA	332	19
Pit-FIP(F1c+F2)-4	TTGTCAGGTTCCTTGCCGAGCAAGCTCAACCGGAGATG		38
Pit-BIP(B1c+B2)-4	CATCCCAGACGCGCTCATGATTAGCTTGTTCAGATCGTCT		40
Pit-LoopF-4	AACCTGTGAAGCCGAACC	166	18

Pit-LoopB-4	ATCAAGAATCCGCCTGCC	229	18
Pit-F2-4	CAAGCTCAACCGGAGATG	129	18
Pit-F1c-4	TTGTCAGGTTCCTTGCCGAG	188	20
Pit-B2-4	GATTAGCTTGTTCAGATCGTCT	273	22
Pit-B1c-4	CATCCCAGACGCGCTCAT	210	18
Product	CAAGCTCAACCGGAGATGGTGGTTCGGCTTCACAGGTTCGCTCGGCAAGGAACCTGACAACGGCCAAGTACAGATCAAGATCATCCCAGACGCGCTCATCATCAAGAATCCGCCTGCCAACAAAGACGATCTGAACAAGCTAATC		145
Pep-F3-1	GACTCGTGTACCAATGCC	328	18
Pep-B3-1	TACCGATTCCTCCTAGCAG	520	19
Pep-FIP(F1c+F2)-1	TGGCTTGAACCGCATCGTAAGGTATCTTCAATGTGATTGTGC		42
Pep-BIP(B1c+B2)-1	AGGGTGGCACTGGAGGAATCCTGGTCGTTAGAGTCTG		37
Pep-LoopF-1	GCCTGAGATCCAAGAGCATT	401	20
Pep-LoopB-1	CACTGACGACGACACCTC	444	18
Pep-F2-1	GGTATCTTCAATGTGATTGTGC	349	22
Pep-F1c-1	TGGCTTGAACCGCATCGTAA	421	20
Pep-B2-1	TCCTGGTCGTTAGAGTCTG	485	19
Pep-B1c-1	AGGGTGGCACTGGAGGAA	422	18
Product	GGTATCTTCAATGTGATTGTGCCCGCTTCGTCCAATGCTCTTGGATCTCAGGCTTACGATGCGGTTCAAGCCAAGGGTGGCACTGGAGGAACCGGCACTGACGACGACACCTCGGCCCCAGACTCTAACGACCAGGA		137
**Pep-F3-2**	TCGTGTACCAATGCCAAAG	331	19
**Pep-B3-2**	TACCGATTCCTCCTAGCAG	520	19
**Pep-FIP(F1c+F2)-2**	TGGCTTGAACCGCATCGTAAGTATCTTCAATGTGATTGTGCC		42
**Pep-BIP(B1c+B2)-2**	AGGGTGGCACTGGAGGAATCCTGGTCGTTAGAGTCTG		37

**Pep-LoopF-2**	GCCTGAGATCCAAGAGCATT	401	20
**Pep-LoopB-2**	CACTGACGACGACACCTC	444	18
**Pep-F2-2**	GTATCTTCAATGTGATTGTGCC	350	22
**Pep-F1c-2**	TGGCTTGAACCGCATCGTAA	421	20
**Pep-B2-2**	TCCTGGTCGTTAGAGTCTG	485	19
**Pep-B1c-2**	AGGGTGGCACTGGAGGAA	422	18
**Product**	GTATCTTCAATGTGATTGTGCCCGCTTCGTCCAATGCTCTTGGATCTCAGGCTTACGATGCGGTTCAAGCCAA GGGTGGCACTGGAGGAACCGGCACTGACGACGACACCTCGGCCCCAGACTCTAACGACCAGGA		136
Pep-F3-3	CAACAATTCGTACACACTGC	252	20
Pep-B3-3	TACCGATTCCTCCTAGCAG	520	19
Pep-FIP(F1c+F2)-3	CGTAAGCCTGAGATCCAAGAGCGACTCGTGTACCAATGCC		40
Pep-BIP(B1c+B2)-3	AAGGGTGGCACTGGAGGATCCTGGTCGTTAGAGTCTG		37
Pep-LoopF-3	CGGGCACAATCACATTGAAG	373	20
Pep-LoopB-3	CACTGACGACGACACCTC	444	18
Pep-F2-3	GACTCGTGTACCAATGCC	328	18
Pep-F1c-3	CGTAAGCCTGAGATCCAAGAGC	406	22
Pep-B2-3	TCCTGGTCGTTAGAGTCTG	485	19
Pep-B1c-3	AAGGGTGGCACTGGAGGA	421	18
Product	GACTCGTGTACCAATGCCAAAGGTATCTTCAATGTGATTGTGCCCGCTTCGTCCAATGCTCTTGGATCTCAGG CTTACGATGCGGTTCAAGCCAAGGGTGGCACTGGAGGAACCGGCACTGACGACGACACCTCGGCCCCAGA CTCTAACGACCAGGA		158
Pep-F3-4	AGGGACAACAATTCGTACAC	247	20
Pep-B3-4	TACCGATTCCTCCTAGCAG	520	19
Pep-FIP(F1c+F2)-4	CGTAAGCCTGAGATCCAAGAGCGCGACTCGTGTACCAATG		40
Pep-BIP(B1c+B2)-4	AAGGGTGGCACTGGAGGATCCTGGTCGTTAGAGTCTG		37

Pep-LoopF-4	CGGGCACAATCACATTGAAG	373	20
Pep-LoopB-4	CACTGACGACGACACCTC	444	18
Pep-F2-4	GCGACTCGTGTACCAATG	326	18
Pep-F1c-4	CGTAAGCCTGAGATCCAAGAGC	406	22
Pep-B2-4	TCCTGGTCGTTAGAGTCTG	485	19
Pep-B1c-4	AAGGGTGGCACTGGAGGA	421	18
Product	GCGACTCGTGTACCAATGCCAAAGGTATCTTCAATGTGATTGTGCCCGCTTCGTCCAATGCTCTTGGATCTCA GGCTTACGATGCGGTTCAAGCCAAGGGTGGCACTGGAGGAACCGGCACTGACGACGACACCTCGGCCCCA GACTCTAACGACCAGGA		160
mPep-F3-1	ACTGGTTCTCCTCGGTC	146	17
mPep-B3-1	TACCGATTCCTCCTAGCAG	535	19
mPep-FIP(F1c+F2)-1	AGGCACAATCACATTGAAGATACCTGGACAACAATTCGTACACA		44
mPep-BIP(B1c+B2)-1	CTCAGGCTTACGATGCGGTTTGTCGTCGTCAGTGC		35
mPep-LoopF-1	TGGCATTGGTACACGAGTC	346	19
mPep-LoopB-1	AGGAGCAGGCACTGGA	435	16
mPep-F2-1	GGACAACAATTCGTACACA	249	19
mPep-F1c-1	AGGCACAATCACATTGAAGATACCT	372	25
mPep-B2-1	TGTCGTCGTCAGTGC	472	15
mPep-B1c-1	CTCAGGCTTACGATGCGGTT	395	20
Product	CGCTCAAGCCGCTTGGAAAAGCATTTTCGAGCAACGTGCGCGACTCGTGTACCAATGCCAAAGGTATCTTC AATGTGATTGTGCCTGCTTCGTCCAATGCTCTTGGATCTCAGGCTTACGATGCGGTTCAAGCCAAGGGTGG CACCGGAGGAGCAGGCACTGGAGGAACCGGCACTGACGACGACA		224
**mPep-F3-2**	GACTCGTGTACCAATGCC	328	18
**mPep-B3-2**	TACCGATTCCTCCTAGCAG	535	19

**mPep-FIP(F1c+F2)-2**	TGGCTTGAACCGCATCGTAATGCCTGCTTCGTCCA		35
**mPep-BIP(B1c+B2)-2**	GTGGCACCGGAGGAGCTGTCGTCGTCAGTGC		31
**mPep-LoopF-2**	GCCTGAGATCCAAGAGCAT	401	19
**mPep-LoopB-2**	GGCACTGGAGGAACCG	442	16
**mPep-F2-2**	TGCCTGCTTCGTCCA	368	15
**mPep-F1c-2**	TGGCTTGAACCGCATCGTAA	421	20
**mPep-B2-2**	TGTCGTCGTCAGTGC	472	15
**mPep-B1c-2**	GTGGCACCGGAGGAGC	425	16
Product	TGCCTGCTTCGTCCAATGCTCTTGGATCTCAGGCTTACGATGCGGTTCAAGCCAAGGGTGGCACCGGAGGA GCAGGCACTGGAGGAACCGGCACTGACGACGACA		105
mPep-F3-3	GCGACTCGTGTACCAATG	326	18
mPep-B3-3	TACCGATTCCTCCTAGCAG	535	19
mPep-FIP(F1c+F2)-3	TGGCTTGAACCGCATCGTAAATTGTGCCTGCTTCGTC		37
mPep-BIP(B1c+B2)-3	GTGGCACCGGAGGAGCTGTCGTCGTCAGTGC		31
mPep-LoopF-3	GCCTGAGATCCAAGAGCATT	401	20
mPep-LoopB-3	GGCACTGGAGGAACCG	442	16
mPep-F2-3	ATTGTGCCTGCTTCGTC	364	17
mPep-F1c-3	TGGCTTGAACCGCATCGTAA	421	20
mPep-B2-3	TGTCGTCGTCAGTGC	472	15
mPep-B1c-3	GTGGCACCGGAGGAGC	425	16
Product	ATTGTGCCTGCTTCGTCCAATGCTCTTGGATCTCAGGCTTACGATGCGGTTCAAGCCAAGGGTGGCACCGG AGGAGCAGGCACTGGAGGAACCGGCACTGACGACGACA		109
mPep-F3-4	GCGACTCGTGTACCAAT	326	17
mPep-B3-4	TACCGATTCCTCCTAGCAG	535	19
mPep-FIP(F1c+F2)-4	TGGCTTGAACCGCATCGTAAGATTGTGCCTGCTTCGT		37

mPep-BIP(B1c+B2)-4	GTGGCACCGGAGGAGCTGTCGTCGTCAGTGC		31
mPep-LoopF-4	GCCTGAGATCCAAGAGCATT	401	20
mPep-LoopB-4	GGCACTGGAGGAACCG	442	16
mPep-F2-4	GATTGTGCCTGCTTCGT	363	17
mPep-F1c-4	TGGCTTGAACCGCATCGTAA	421	20
mPep-B2-4	TGTCGTCGTCAGTGC	472	15
mPep-B1c-4	GTGGCACCGGAGGAGC	425	16
Product	GATTGTGCCTGCTTCGTCCAATGCTCTTGGATCTCAGGCTTACGATGCGGTTCAAGCCAAGGGTGGCACCG GAGGAGCAGGCACTGGAGGAACCGGCACTGACGACGACA		110
mPep-F3-5	GCGACTCGTGTACCAA	326	16
mPep-B3-5	TACCGATTCCTCCTAGCAG	535	19
mPep-FIP(F1c+F2)-5	TGGCTTGAACCGCATCGTAATGATTGTGCCTGCTTCG		37
mPep-BIP(B1c+B2)-5	GTGGCACCGGAGGAGCTGTCGTCGTCAGTGC		31
mPep-LoopF-5	GCCTGAGATCCAAGAGCATT	401	20
mPep-LoopB-5	GGCACTGGAGGAACCG	442	16
mPep-F2-5	TGATTGTGCCTGCTTCG	362	17
mPep-F1c-5	TGGCTTGAACCGCATCGTAA	421	20
mPep-B2-5	TGTCGTCGTCAGTGC	472	15
mPep-B1c-5	GTGGCACCGGAGGAGC	425	16
Product	TGATTGTGCCTGCTTCGTCCAATGCTCTTGGATCTCAGGCTTACGATGCGGTTCAAGCCAAGGGTGGCACC GGAGGAGCAGGCACTGGAGGAACCGGCACTGACGACGACA		111
mPep-F3-6	GGACAACAATTCGTACACA	249	19
mPep-B3-6	TACCGATTCCTCCTAGCAG	535	19
mPep-FIP(F1c+F2)-6	AACCGCATCGTAAGCCTGAGGGTATCTTCAATGTGATTGTGC		42
mPep-BIP(B1c+B2)-6	GTGGCACCGGAGGAGCTGTCGTCGTCAGTGC		31

mPep-LoopF-6	AAGAGCATTGGACGAAGCA	390	19
mPep-LoopB-6	GGCACTGGAGGAACCG	442	16
mPep-F2-6	GGTATCTTCAATGTGATTGTGC	349	22
mPep-F1c-6	AACCGCATCGTAAGCCTGAG	414	20
mPep-B2-6	TGTCGTCGTCAGTGC	472	15
mPep-B1c-6	GTGGCACCGGAGGAGC	425	16
Product	GGTATCTTCAATGTGATTGTGCCTGCTTCGTCCAATGCTCTTGGATCTCAGGCTTACGATGCGGTTCAAGCC AAGGGTGGCACCGGAGGAGCAGGCACTGGAGGAACCGGCACTGACGACGACA		124
mPep-F3-7	CAAGCCACGCTTCTCT	19	16
mPep-B3-7	TACCGATTCCTCCTAGCAG	535	19
mPep-FIP(F1c+F2)-7	CGCCGACTTCGACCGAGCGTCCATGCCGATGC		32
mPep-BIP(B1c+B2)-7	CTCAGGCTTACGATGCGGTTTGTCGTCGTCAGTGC		35
mPep-LoopF-7	GGCAATGGTACCGCACC	104	17
mPep-LoopB-7	AGGAGCAGGCACTGGA	435	16
mPep-F2-7	CGTCCATGCCGATGC	69	15
mPep-F1c-7	CGCCGACTTCGACCGAG	172	17
mPep-B2-7	TGTCGTCGTCAGTGC	472	15
mPep-B1c-7	CTCAGGCTTACGATGCGGTT	395	20
Product	CGTCCATGCCGATGCTGCGGGTGCGGTACCATTGCCGAATTTCAAGGTAGACCCGCAACCTCTGGCCAGC ACGTTTTACTGGTTCTCCTCGGTCGAAGTCGGCGTTTGCTACAACCCGCAAGCTCGCGTTGGCAGCACCAA AGGCGCTCTTCACTGCACACACCAAGAAAACTACGACAGGGACAACAATTCGTACACACTGCCGCAAACGT GCGTGGCGCTCAAGCCGCTTGGAAAAGCATTTTCGAGCAACGTGCGCGACTCGTGTACCAATGCCAAAGGT ATCTTCAATGTGATTGTGCCTGCTTCGTCCAATGCTCTTGGATCTCAGGCTTACGATGCGGTTCAAGCCAAG GGTGGCACCGGAGGAGCAGGCACTGGAGGAACCGGCACTGACGACGACA		404

* Primers name in bold type indicates the optimum RealAmp primer sets Pep-2 and mPep-2 that designed for *U. maydis* of Clade A and Clade B, respectively.

Supplementary Table S2. Quantification of soil and maize plants samples by RealAmp assay using ZYD-S1 Soil Samples

Soil samples

Samples NO.	Field[a]	Tt	spores g-1soil			LAMP products amplification	Sequencing analysis[b]	Growth on media[c]	PCR with Pok317/Pok318	Sequencing analysis[d]
			Test 1	Test 2	Average					
1	A1	12.25±0.25	5.62E+04	9.45E+04	7.53E+04	+	Clade A	+	+	Clade A
2	A1	15±0	4.16E+03	4.16E+03	4.16E+03	+	Clade A	+	+	Clade A
3	A1	10.75±0.25	4.51E+05	2.68E+05	3.59E+05	+	Clade A	+	+	Clade A
4	A1	11.75±0.25	9.45E+04	1.59E+05	1.27E+05	+	Clade A	+	+	Clade A
5	A1	13±0	3.34E+04	3.34E+04	3.34E+04	+	Clade A	+	+	Clade A
6	A2	12.75±0.25	5.62E+04	3.34E+04	4.48E+04	+	Clade A	+	+	Clade A
7	A2	10.5±0	4.51E+05	4.51E+05	4.51E+05	+	Clade A	+	+	Clade A
8	A2	14.5±0	7.00E+03	7.00E+03	7.00E+03	+	Clade A	+	+	Clade A
9	A2	11.5±0.5	2.68E+05	9.45E+04	1.81E+05	+	Clade A	+	+	Clade A
10	A2	11.75±0.25	1.59E+05	9.45E+04	1.27E+05	+	Clade A	+	+	Clade A
11	A3	15.5±0.5	4.16E+03	1.47E+03	2.81E+03	+	Clade A	+	+	Clade A
12	A3	15.25±0.25	4.16E+03	2.47E+03	3.31E+03	+	Clade A	+	+	Clade A
13	A3	15±0	4.16E+03	4.16E+03	4.16E+03	+	Clade A	+	+	Clade A
14	A3	15.25±0.25	2.47E+03	4.16E+03	3.31E+03	+	Clade A	+	+	Clade A
15	A3	14.25±0.25	7.00E+03	1.18E+04	9.39E+03	+	Clade A	+	+	Clade A
16	A4	11.25±0.25	1.59E+05	2.68E+05	2.13E+05	+	Clade A	+	+	Clade A
17	A4	12.25±0.25	5.62E+04	9.45E+04	7.53E+04	+	Clade A	+	+	Clade A
18	A4	10.5±0	4.51E+05	4.51E+05	4.51E+05	+	Clade A	+	+	Clade A
19	A4	12.75±0.25	3.34E+04	5.62E+04	4.48E+04	+	Clade A	+	+	Clade A
20	A4	NA	-	-	-	-	N/A	-	-	N/A

21	A5	12.75±0.25	3.34E+04	5.62E+04	4.48E+04	+	Clade A	+	+	Clade A
22	A5	10.25±0.25	4.51E+05	7.59E+05	6.05E+05	+	Clade A	+	+	Clade A
23	A5	9.25±0.25	1.28E+06	2.15E+06	1.71E+06	+	Clade A	+	+	Clade A
24	A5	12.25±0.25	5.62E+04	9.45E+04	7.53E+04	+	Clade A	+	+	Clade A
25	A5	N/A	-	-	-	-	N/A	-	-	N/A
26	B1	N/A	-	-	-	-	N/A	-	-	N/A
27	B1	N/A	-	-	-	-	N/A	-	-	N/A
28	B1	N/A	-	-	-	-	N/A	-	-	N/A
29	B1	N/A	-	-	-	-	N/A	-	-	N/A
30	B1	N/A	-	-	-	-	N/A	-	-	N/A
31	B2	13.25±0.25	1.98E+04	3.34E+04	2.66E+04	+	Clade A	+	+	Clade A
32	B2	13.25±0.25	1.98E+04	3.34E+04	2.66E+04	+	Clade A	+	+	Clade A
33	B2	7.5±0	1.03E+07	1.03E+07	1.03E+07	+	Clade A	+	+	Clade A
34	B2	8.25±0.25	3.62E+06	6.09E+06	4.86E+06	+	Clade A	+	+	Clade A
35	B2	14.5±0	7.00E+03	7.00E+03	7.00E+03	+	Clade A	+	+	Clade A
36	B3	13.35±0.15	1.98E+04	2.71E+04	2.35E+04	+	Clade A	+	+	Clade A
37	B3	10.5±0	4.51E+05	4.51E+05	4.51E+05	+	Clade A	+	+	Clade A
38	B3	N/A	-	-	-	-	N/A	-	-	N/A
39	B3	11.5±0	1.59E+05	1.59E+05	1.59E+05	+	Clade A	+	+	Clade A
40	B3	12.5±0.5	9.45E+04	3.34E+04	6.39E+04	+	Clade A	+	+	Clade A
41	B4	10.75±0.25	4.51E+05	2.68E+05	3.59E+05	+	Clade A	+	+	Clade A
42	B4	10.75±0.25	2.68E+05	4.51E+05	3.59E+05	+	Clade A	+	+	Clade A
43	B4	14.75±0.25	7.00E+03	4.16E+03	5.58E+03	+	Clade A	+	+	Clade A
44	B4	14.75±0.25	7.00E+03	4.16E+03	5.58E+03	+	Clade A	+	+	Clade A
45	B4	13.75±0.25	1.98E+04	1.18E+04	1.58E+04	+	Clade A	+	+	Clade A

46	B5	8.75±0.25	2.15E+06	3.62E+06	2.88E+06	+	Clade A	+	+	Clade A
47	B5	12.25±0.25	9.45E+04	5.62E+04	7.53E+04	+	Clade A	+	+	Clade A
48	B5	8.75±0.25	3.62E+06	2.15E+06	2.88E+06	+	Clade A	+	+	Clade A
49	B5	10.5±0	4.51E+05	4.51E+05	4.51E+05	+	Clade A	+	+	Clade A
50	B5	N/A	-	-	-	-	N/A	-	-	N/A
51	C1	13.75±0.25	1.98E+04	1.18E+04	1.58E+04	+	Clade A	+	+	Clade A
52	C1	13.5±0	1.98E+04	1.98E+04	1.98E+04	+	Clade A	+	+	Clade A
53	C1	12.25±0.25	5.62E+04	9.45E+04	7.53E+04	+	Clade A	+	+	Clade A
54	C1	N/A	-	-	-	-	N/A	-	-	N/A
55	C1	14.75±0.25	7.00E+03	4.16E+03	5.58E+03	+	Clade A	+	+	Clade A
56	C2	10.75±0.25	4.51E+05	2.68E+05	3.59E+05	+	Clade A	+	+	Clade A
57	C2	10±0	7.59E+05	7.59E+05	7.59E+05	+	Clade A	+	+	Clade A
58	C2	13.25±0.25	1.98E+04	3.34E+04	2.66E+04	+	Clade A	+	+	Clade A
59	C2	10±0.5	1.28E+06	4.51E+05	8.64E+05	+	Clade A	+	+	Clade A
60	C2	13.25±0.25	1.98E+04	3.34E+04	2.66E+04	+	Clade A	+	+	Clade A
61	C3	N/A	-	-	-	-	N/A	-	-	N/A
62	C3	12.25±0.25	5.62E+04	9.45E+04	7.53E+04	+	Clade A	+	+	Clade A
63	C3	15.25±0.25	4.16E+03	2.47E+03	3.31E+03	+	Clade A	+	+	Clade A
64	C3	13.25±0.25	1.98E+04	3.34E+04	2.66E+04	+	Clade A	+	+	Clade A
65	C3	15.75±0.25	2.47E+03	1.47E+03	1.97E+03	+	Clade A	+	+	Clade A
66	C4	N/A	-	-	-	-	N/A	-	-	N/A
67	C4	N/A	-	-	-	-	N/A	-	-	N/A
68	C4	15±0.5	7.00E+03	2.47E+03	4.73E+03	+	Clade A	+	+	Clade A
69	C4	16±0.5	2.47E+03	8.72E+02	1.67E+03	+	Clade A	+	+	Clade A
70	C4	11±0.5	4.51E+05	1.59E+05	3.05E+05	+	Clade A	+	+	Clade A

71	C5	N/A	-	-	-	-	N/A	-	-	N/A
72	C5	11.75±0.25	1.59E+05	9.45E+04	1.27E+05	+	Clade A	+	+	Clade A
73	C5	9±0.5	3.62E+06	1.28E+06	2.45E+06	+	Clade A	+	+	Clade A
74	C5	11.75±0.25	1.59E+05	9.45E+04	1.27E+05	+	Clade A	+	+	Clade A
75	C5	12.5±0.5	9.45E+04	3.34E+04	6.39E+04	+	Clade A	+	+	Clade A
76	D1	N/A	-	-	-	-	N/A	-	-	N/A
77	D1	N/A	-	-	-	-	N/A	-	-	N/A
78	D1	N/A	-	-	-	-	N/A	-	-	N/A
79	D1	N/A	-	-	-	-	N/A	-	-	N/A
80	D1	N/A	-	-	-	-	N/A	-	-	N/A
81	D2	13.75±0.25	1.98E+04	1.18E+04	1.58E+04	+	Clade A	+	+	Clade A
82	D2	12.75±0.25	5.62E+04	3.34E+04	4.48E+04	+	Clade A	+	+	Clade A
83	D2	14.5±0.5	1.18E+04	4.16E+03	7.97E+03	+	Clade A	+	+	Clade A
84	D2	13.75±0.25	1.18E+04	1.98E+04	1.58E+04	+	Clade A	+	+	Clade A
85	D2	13.5±0.5	3.34E+04	1.18E+04	2.26E+04	+	Clade A	+	+	Clade A
86	D3	N/A	-	-	-	-	N/A	-	-	N/A
87	D3	N/A	-	-	-	-	N/A	-	-	N/A
88	D3	12.25±0.25	5.62E+04	9.45E+04	7.53E+04	+	Clade A	+	+	Clade A
89	D3	14±0.5	7.00E+03	1.98E+04	1.34E+04	+	Clade A	+	+	Clade A
90	D3	12.25±0.25	5.62E+04	9.45E+04	7.53E+04	+	Clade A	+	+	Clade A
91	D4	11.25±0.25	1.59E+05	2.68E+05	2.13E+05	+	Clade A	+	+	Clade A
92	D4	13±0.5	5.62E+04	1.98E+04	3.80E+04	+	Clade A	+	+	Clade A
93	D4	10.5±0.5	7.59E+05	2.68E+05	5.13E+05	+	Clade A	+	+	Clade A
94	D4	10±0.5	1.28E+06	4.51E+05	8.64E+05	+	Clade A	+	+	Clade A
95	D4	12±0.5	1.59E+05	5.62E+04	1.08E+05	+	Clade A	+	+	Clade A

96	D5	11.5±0.5	2.68E+05	9.45E+04	1.81E+05	+	Clade A	+	+	Clade A
97	D5	12.5±0.5	9.45E+04	3.34E+04	6.39E+04	+	Clade A	+	+	Clade A
98	D5	14.5±0.5	1.18E+04	4.16E+03	7.97E+03	+	Clade A	+	+	Clade A
99	D5	10.75±0.25	2.68E+05	4.51E+05	3.59E+05	+	Clade A	+	+	Clade A
100	D5	12.5±0.5	9.45E+04	3.34E+04	6.39E+04	+	Clade A	+	+	Clade A
101	E1	11.75±0.25	1.59E+05	9.45E+04	1.27E+05	+	Clade B	+	+	Clade B
102	E1	10.5±0	4.51E+05	4.51E+05	4.51E+05	+	Clade B	+	+	Clade B
103	E1	12.75±0.25	5.62E+04	3.34E+04	4.48E+04	+	Clade B	+	+	Clade B
104	E1	10.35±0.35	7.59E+05	3.66E+05	5.62E+05	+	Clade B	+	+	Clade B
105	E1	9.75±0.25	1.28E+06	7.59E+05	1.02E+06	+	Clade B	+	+	Clade B
106	E2	14.75±0.25	7.00E+03	4.16E+03	5.58E+03	+	Clade B	+	+	Clade B
107	E2	84.25±69.75	7.00E+03	5.64E-60	3.50E+03	+	Clade B	+	+	Clade B
108	E2	12.25±0.25	5.62E+04	9.45E+04	7.53E+04	+	Clade B	+	+	Clade B
109	E2	15.75±0.25	2.47E+03	1.47E+03	1.97E+03	+	Clade B	+	+	Clade B
110	E2	14.25±0.25	7.00E+03	1.18E+04	9.39E+03	+	Clade B	+	+	Clade B
111	E3	13.75±0.25	1.98E+04	1.18E+04	1.58E+04	+	Clade B	+	+	Clade B
112	E3	10.25±0.25	4.51E+05	7.59E+05	6.05E+05	+	Clade B	+	+	Clade B
113	E3	10±0	7.59E+05	7.59E+05	7.59E+05	+	Clade B	+	+	Clade B
114	E3	9.25±0.25	2.15E+06	1.28E+06	1.71E+06	+	Clade B	+	+	Clade B
115	E3	11±0.5	1.59E+05	4.51E+05	3.05E+05	+	Clade B	+	+	Clade B
116	E4	12±0.5	1.59E+05	5.62E+04	1.08E+05	+	Clade B	+	+	Clade B
117	E4	8.5±0	3.62E+06	3.62E+06	3.62E+06	+	Clade B	+	+	Clade B
118	E4	7.75±0.25	1.03E+07	6.09E+06	8.17E+06	+	Clade B	+	+	Clade B
119	E4	12.25±0.25	5.62E+04	9.45E+04	7.53E+04	+	Clade B	+	+	Clade B
120	E4	10.25±0.25	4.51E+05	7.59E+05	6.05E+05	+	Clade B	+	+	Clade B

121	E5	12±0	9.45E+04	9.45E+04	9.45E+04	+	Clade B	+	+	Clade B
122	E5	11±0	2.68E+05	2.68E+05	2.68E+05	+	Clade B	+	+	Clade B
123	E5	10.5±0	4.51E+05	4.51E+05	4.51E+05	+	Clade B	+	+	Clade B
124	E5	12.25±0.25	5.62E+04	9.45E+04	7.53E+04	+	Clade B	+	+	Clade B
125	E5	12±0	9.45E+04	9.45E+04	9.45E+04	+	Clade B	+	+	Clade B
126	F1	14.25±0.25	7.00E+03	1.18E+04	9.39E+03	+	Clade A	+	+	Clade A
127	F1	8.75±0.25	2.15E+06	3.62E+06	2.88E+06	+	Clade A	+	+	Clade A
128	F1	11.5±0	1.59E+05	1.59E+05	1.59E+05	+	Clade A	+	+	Clade A
129	F1	12±0	9.45E+04	9.45E+04	9.45E+04	+	Clade A	+	+	Clade A
130	F1	14.75±0.25	7.00E+03	4.16E+03	5.58E+03	+	Clade A	+	+	Clade A
131	F2	9.5±0.5	2.15E+06	7.59E+05	1.45E+06	+	Clade A	+	+	Clade A
132	F2	10.75±0.25	4.51E+05	2.68E+05	3.59E+05	+	Clade A	+	+	Clade A
133	F2	9.25±0.25	1.28E+06	2.15E+06	1.71E+06	+	Clade A	+	+	Clade A
134	F2	11.75±0.25	1.59E+05	9.45E+04	1.27E+05	+	Clade A	+	+	Clade A
135	F2	12.25±0.25	5.62E+04	9.45E+04	7.53E+04	+	Clade A	+	+	Clade A
136	F3	11±0	2.68E+05	2.68E+05	2.68E+05	+	Clade A	+	+	Clade A
137	F3	12±0	9.45E+04	9.45E+04	9.45E+04	+	Clade A	+	+	Clade A
138	F3	11.75±0.25	1.59E+05	9.45E+04	1.27E+05	+	Clade A	+	+	Clade A
139	F3	9.5±0	1.28E+06	1.28E+06	1.28E+06	+	Clade A	+	+	Clade A
140	F3	17.5±0	3.08E+02	3.08E+02	3.08E+02	+	Clade A	+	+	Clade A
141	F4	13.75±0.25	1.18E+04	1.98E+04	1.58E+04	+	Clade A	+	+	Clade A
142	F4	12.75±0.25	5.62E+04	3.34E+04	4.48E+04	+	Clade A	+	+	Clade A
143	F4	11.25±0.25	1.59E+05	2.68E+05	2.13E+05	+	Clade A	+	+	Clade A
144	F4	13±0.5	5.62E+04	1.98E+04	3.80E+04	+	Clade A	+	+	Clade A
145	F4	10.5±0.5	7.59E+05	2.68E+05	5.13E+05	+	Clade A	+	+	Clade A

146	F5	10.5±0	4.51E+05	4.51E+05	4.51E+05	+	Clade A	+	+	Clade A
147	F5	11.25±0.25	1.59E+05	2.68E+05	2.13E+05	+	Clade A	+	+	Clade A
148	F5	11.75±0.25	1.59E+05	9.45E+04	1.27E+05	+	Clade A	+	+	Clade A
149	F5	13±0	3.34E+04	3.34E+04	3.34E+04	+	Clade A	+	+	Clade A
150	F5	14.25±0.25	1.18E+04	7.00E+03	9.39E+03	+	Clade A	+	+	Clade A

N/A, not determined.

+, positive result.

-, negative result.

a Filed location.

b Analysis of the sequencing of the LAMP products that amplified with primer sets F2-2/B2-2 or mF2-2/mB2-2.

c Growth on the PDA and $YEPS_L$.

d Analysis of the sequencing of the PCR products that amplified with primers Pok317/Pok318[31].

A, Qiliying town, Xinxiang County, Henan Province

B, Duliang town, Kaifeng City, Henan Province

C, Pohu town, Xuchang City, Henan Province

D, Wucheng town, Wuyang County, Henan Province

E, Baima town, Nanjing, Jiangsu Province

F, Zhengfu town, Fuyang City, Anhui Province

Maize plants samples

Samples NO.	Sample name[a]	Tt	U. maydis (ng/μL)			LAMP products amplification	Sequencing analysis[b]	Growth on media[c]	PCR with Pok317/Pok318	sequencing analysis[d]
			Test 1	Test 2	Average					
1	A-1	22±0	0.248995	0.248995	0.248995	+	Clade A	+	+	Clade A
2	A-2	18±0.5	1.3130764	1.0766139	1.1948451	+	Clade A	+	+	Clade A
3	A-3	18.5±0	1.0766139	1.0766139	1.0766139	+	Clade A	+	+	Clade A
4	A-4	22.5±0.5	0.248995	0.0125325	0.1307638	+	Clade A	+	+	Clade A
5	A-5	20±0.5	0.8401513	0.6036888	0.7219201	+	Clade A	+	+	Clade A
6	A-6	22.75±0.25	0.1307638	0.0125325	0.0716481	+	Clade A	+	+	Clade A
7	A-7	22.5±0.5	0.0125325	0.248995	0.1307638	+	Clade A	+	+	Clade A
8	A-8	17.25±0.25	1.3130764	1.4313076	1.372192	+	Clade A	+	+	Clade A
9	A-9	22.5±0.5	0.0125325	0.248995	0.1307638	+	Clade A	+	+	Clade A
10	B1-1	22±0.5	0.1307638	0.3672263	0.248995	+	Clade A	+	+	Clade A
11	B1-2	21±0.5	0.6036888	0.3672263	0.4854576	+	Clade A	+	+	Clade A
12	B1-3	22.75±0.25	0.0125325	0.1307638	0.0716481	+	Clade A	+	+	Clade A
13	B1-4	17±0	1.4313076	1.4313076	1.4313076	+	Clade A	+	+	Clade A
14	B1-5	15.5±0.5	1.9042327	1.6677702	1.7860014	+	Clade A	+	+	Clade A
15	B1-6	11.75±0.25	2.7318515	2.6136202	2.6727359	+	Clade A	+	+	Clade A
16	B1-7	13.25±0.25	2.3771577	2.2589265	2.3180421	+	Clade A	+	+	Clade A
17	B1-8	19±0	0.9583826	0.9583826	0.9583826	+	Clade A	+	+	Clade A
18	B1-9	18.25±0.25	1.1948451	1.0766139	1.1357295	+	Clade A	+	+	Clade A
19	B2-1	18.75±0.25	0.9583826	1.0766139	1.0174982	+	Clade A	+	+	Clade A
20	B2-2	16.75±0.25	1.5495389	1.4313076	1.4904233	+	Clade A	+	+	Clade A

21	B2-3	17±0.5	1.3130764	1.5495389	1.4313076	+	Clade A	+	+	Clade A
22	B2-4	13.75±0.25	2.2589265	2.1406952	2.1998108	+	Clade A	+	+	Clade A
23	B2-5	18±0.5	1.0766139	1.3130764	1.1948451	+	Clade A	+	+	Clade A
24	B2-6	15.25±0.25	1.7860014	1.9042327	1.845117	+	Clade A	+	+	Clade A
25	B2-7	18.5±0	1.0766139	1.0766139	1.0766139	+	Clade A	+	+	Clade A
26	B2-8	13.25±0.25	2.2589265	2.3771577	2.3180421	+	Clade A	+	+	Clade A
27	B2-9	20.25±0.25	0.6036888	0.7219201	0.6628044	+	Clade A	+	+	Clade A
28	C-1	22.25±0.25	0.248995	0.1307638	0.1898794	+	Clade A	+	+	Clade A
29	C-2	21.5±0.5	0.248995	0.4854576	0.3672263	+	Clade A	+	+	Clade A
30	C-3	22.75±0.25	0.0125325	0.1307638	0.0716481	+	Clade A	+	+	Clade A
31	C-4	23±0	0.0125325	0.0125325	0.0125325	+	Clade A	+	+	Clade A
32	C-5	22.5±0.5	0.248995	0.0125325	0.1307638	+	Clade A	+	+	Clade A
33	C-6	16.25±0.25	1.6677702	1.5495389	1.6086545	+	Clade A	+	+	Clade A
34	C-7	18.5±0.5	1.1948451	0.9583826	1.0766139	+	Clade A	+	+	Clade A
35	C-8	20.25±0.25	0.7219201	0.6036888	0.6628044	+	Clade A	+	+	Clade A
36	C-9	16.25±0.25	1.5495389	1.6677702	1.6086545	+	Clade A	+	+	Clade A
37	D-1	20.5±0	0.6036888	0.6036888	0.6036888	+	Clade A	+	+	Clade A
38	D-2	22±0.5	0.1307638	0.3672263	0.248995	+	Clade A	+	+	Clade A
39	D-3	22.75±0.25	0.1307638	0.0125325	0.0716481	+	Clade A	+	+	Clade A
40	D-4	20.5±0.5	0.7219201	0.4854576	0.6036888	+	Clade A	+	+	Clade A
41	D-5	21±0.5	0.3672263	0.6036888	0.4854576	+	Clade A	+	+	Clade A
42	D-6	14.5±0.5	2.1406952	1.9042327	2.0224639	+	Clade A	+	+	Clade A
43	D-7	14.25±0.25	2.1406952	2.0224639	2.0815796	+	Clade A	+	+	Clade A
44	D-8	14±0.5	2.2589265	2.0224639	2.1406952	+	Clade A	+	+	Clade A
45	D-9	19.5±0	0.8401513	0.8401513	0.8401513	+	Clade A	+	+	Clade A

46	M-1	N/A	-	-	-	-	N/A	-	-	N/A
47	M-2	N/A	-	-	-	-	N/A	-	-	N/A
48	M-3	20.25±0.25	0.7219201	0.6036888	0.6628044	+	Clade A	+	+	Clade A
49	M-4	18.5±0	1.0766139	1.0766139	1.0766139	+	Clade A	+	+	Clade A
50	M-5	22.5±0.5	0.0125325	0.248995	0.1307638	+	Clade A	+	+	Clade A
51	M-6	20.75±0.25	0.6036888	0.4854576	0.5445732	+	Clade A	+	+	Clade A
52	M-7	16.25±0.25	1.5495389	1.6677702	1.6086545	+	Clade A	+	+	Clade A
53	M-8	15.5±0	1.7860014	1.7860014	1.7860014	+	Clade A	+	+	Clade A
54	M-9	15±0.5	2.0224639	1.7860014	1.9042327	+	Clade A	+	+	Clade A
55	S-1	N/A	-	-	-	-	N/A	-	-	N/A
56	S-2	N/A	-	-	-	-	N/A	-	-	N/A
57	S-3	N/A	-	-	-	-	N/A	-	-	N/A
58	S-4	N/A	-	-	-	-	N/A	-	-	N/A
59	S-5	N/A	-	-	-	-	N/A	-	-	N/A
60	S-6	N/A	-	-	-	-	N/A	-	-	N/A
61	S-7	N/A	-	-	-	-	N/A	-	-	N/A
62	S-8	N/A	-	-	-	-	N/A	-	-	N/A
63	S-9	N/A	-	-	-	-	N/A	-	-	N/A
64	Z-1	22.25±0.25	0.1307638	0.248995	0.1898794	+	Clade A	+	+	Clade A
65	Z-2	17.75±0.25	1.3130764	1.1948451	1.2539607	+	Clade A	+	+	Clade A
66	Z-3	19.25±0.25	0.8401513	0.9583826	0.899267	+	Clade A	+	+	Clade A
67	Z-4	18.75±0.25	1.0766139	0.9583826	1.0174982	+	Clade A	+	+	Clade A
68	Z-5	20.75±0.25	0.6036888	0.4854576	0.5445732	+	Clade A	+	+	Clade A
69	Z-6	18±0	1.1948451	1.1948451	1.1948451	+	Clade A	+	+	Clade A
70	Z-7	16.5±0.5	1.4313076	1.6677702	1.5495389	+	Clade A	+	+	Clade A

71	Z-8	15±0.5	2.0224639	1.7860014	1.9042327	+	Clade A	+	+	Clade A
72	Z-9	21.25±0.25	0.3672263	0.4854576	0.4263419	+	Clade A	+	+	Clade A

N/A, not determined.

+, positive result.

-, negative result.

a Genotypes of maize plants samples.

b Analysis of the sequencing of the LAMP products that amplified with primer sets F2-2/B2-2 or mF2-2/mB2-2.

c Growth on the PDA and $YEPS_L$.

d Analysis of the sequencing of the PCR products that amplified with primers Pok317/Pok318[31].

A, A801.

B1, B73.

B2, B104.

C, Chang7-2.

D, Dan340.

M, Mo17.

S, Shen137.

Z, Zheng58.
