# Supplementary material for: Development of a real-time fluorescence loop-mediated isothermal amplification assay for rapid and quantitative detection of Ustilago maydis
Source: Sci Rep. 2017 Oct 17;7:13394. doi: 10.1038/s41598-017-13881-4 (PMC5645423; doi:10.1038/s41598-017-13881-4)
Supplement: Supplementary file 2 — Supplementary Table S1 [file 41598_2017_13881_MOESM2_ESM.pdf]

TITLE:

Development of a real-time fluorescence loop-mediated isothermal amplification assay for rapid and quantitative detection of *Ustilago maydis*

AUTHORS:

Yanyong Cao[1, +], Lifeng Wang[1, +], Liping Duan[2], Jingjing Li[1], Juan Ma[1], Shuna Xie[3], Lei Shi[2] & Huiyong Li[1]

AFFILIATION:

[1] Institute of Cereal Crops, Henan Academy of Agricultural Sciences, Zhengzhou, 450002, China

[2] Institute of Food Safety and Nutrition, Jinan University, Guangzhou, 510632, China

[3] Institute of Plant Protection, Henan Academy of Agricultural Sciences, Zhengzhou, 450002, China

Correspondence and requests for materials should be addressed to Y.C. (email: yanyongcao@126.com) or H. L. (email: lihuiyong1977@126.com)

+These authors contribute equally to this work.

Supplementary Table S1. The screening of different sets of primers used for *Ustilago maydis* real-time fluorescence loop-mediated isothermal amplification

Name*	Primer sequences (5' to 3')	Position	Length
See-F3-1	GCCATCAACAAGAATTCGC	265	19
See-B3-1	TCGGCCCAAATTTATACTCTC	466	21
See-FIP(F1c+F2)-1	CTGCCTGTCGTACGTGAACTCCACGGAGGACTACATAGACT		41
See-BIP(B1c+B2)-1	CTCGTACAGGTATGGCGATAGCCAGAAGCAGACTGGTGATC		41
See-LoopF-1	CCTTCTCCTTGCACCTGAG	332	19
See-LoopB-1	AAGCGGAGTATTCTGTTGCT	407	20
See-F2-1	CACGGAGGACTACATAGACT	289	20
See-F1c-1	CTGCCTGTCGTACGTGAACTC	354	21
See-B2-1	CAGAAGCAGACTGGTGATC	445	19
See-B1c-1	CTCGTACAGGTATGGCGATAGC	369	22
Product	CACGGAGGACTACATAGACTTCCGGCTCAGGTGCAAGGAGAAGGCGAGTTCACGTACGACAGGCAGAGAA ATGCAGTCGGCTCGTACAGGTATGGCGATAGCCATGGTAACAGTAGGGAAGCGGAGTATTCTGTTGCTGATC ACCAGTCTGCTTCTG		157
See-F3-2	CTTCACCACCTTCGTTTCA	6	19
See-B3-2	CGCTCGTGAATCTGTTCA	224	18
See-FIP(F1c+F2)-2	CAATGGCAGAGGACGAACGACTCCTCGTCATACTCTGTCT		40
See-BIP(B1c+B2)-2	GCAAGCAGAAGCACAAGATCAAGCTCGATGCTGTCTTCAG		40
See-LoopF-2	GTAGAGGATGAGCAGACACG	73	20
See-LoopB-2	GCAATTTGAAGAGGAGATCGC	129	21
See-F2-2	CTCCTCGTCATACTCTGTCT	28	20
See-F1c-2	CAATGGCAGAGGACGAACGA	100	20
See-B2-2	GCTCGATGCTGTCTTCAG	175	18

See-B1c-2	GCAAGCAGAAGCACAAGATCAA	101	22
Product	CTCCTCGTCATACTCTGTCTTGTCCACGTGTCTGCTCATCCTCTACAATCGTTTCGTTCGTCCTCTGCCATTG GCAAGCAGAAGCACAAGATCAAGTCTAGGCAATTTGAAGAGGAGATCGCGCAAGGTGCTGAAGACAGCATC GAGC		148
See-F3-3	CTCCTCGTCATACTCTGTCT	28	20
See-B3-3	GTTTTGTTGCTCTGTGCG	240	18
See-FIP(F1c+F2)-3	GATCTTGTGCTTCTGCTTGCCGTCTGCTCATCCTCTACAATC		42
See-BIP(B1c+B2)-3	ATCGCGCAAGGTGCTGAATTCAGACGAATCGTGAACC		37
See-LoopF-3	AATGGCAGAGGACGAACG	99	18
See-LoopB-3	CGAGCTGTTCGAGTTTCCT	171	19
See-F2-3	GTCTGCTCATCCTCTACAATC	57	21
See-F1c-3	GATCTTGTGCTTCTGCTTGCC	120	21
See-B2-3	TTCAGACGAATCGTGAACC	210	19
See-B1c-3	ATCGCGCAAGGTGCTGAA	145	18
Product	GTCTGCTCATCCTCTACAATCGTTTCGTTCGTCCTCTGCCATTGGCAAGCAGAAGCACAAGATCAAGTCTAGG CAATTTGAAGAGGAGATCGCGCAAGGTGCTGAAGACAGCATCGAGCTGTTCGAGTTTCCTCGGGTTCACGA TTCGTCTGAA		154
See-F3-4	CACCTTCGTTTCACTGCT	12	18
See-B3-4	GTTTTGTTGCTCTGTGCG	240	18
See-FIP(F1c+F2)-4	CAATGGCAGAGGACGAACGACATACTCTGTCTTGTCCACG		40
See-BIP(B1c+B2)-4	ATCGCGCAAGGTGCTGAATTCAGACGAATCGTGAACC		37
See-LoopF-4	AACGATTGTAGAGGATGAGCAG	80	22
See-LoopB-4	CGAGCTGTTCGAGTTTCCT	171	19
See-F2-4	CATACTCTGTCTTGTCCACG	36	20
See-F1c-4	CAATGGCAGAGGACGAACGA	100	20

See-B2-4	TTCAGACGAATCGTGAACC	210	19
See-B1c-4	ATCGCGCAAGGTGCTGAA	145	18
Product	CATACTCTGTCTTGTCCACGTGTCTGCTCATCCTCTACAATCGTTTCGTTCGTCCTCTGCCATTGGCAAGCAG AAGCACAAGATCAAGTCTAGGCAATTTGAAGAGGAGATCGCGCAAGGTGCTGAAGACAGCATCGAGCTGTT CGAGTTTCCTCGGGTTCACGATTCGTCTGAA		175
Pit-F3-1	TTGTTCTGCTCATCGTGG	20	18
Pit-B3-1	CATGACCACCGTCTTGAAT	318	19
Pit-FIP(F1c+F2)-1	GCGAACCTGTGAAGCCGAATCTCTACCGATGCCTCAAT		38
Pit-BIP(B1c+B2)-1	TCGGCAAGGAACCTGACAACGGCGGATTCTTGATGATGA		39
Pit-LoopF-1	CCACCATCTCCGGTTGAG	150	18
Pit-LoopB-1	GGCCAAGTACAGATCAAGATCA	190	22
Pit-F2-1	TCTCTACCGATGCCTCAAT	95	19
Pit-F1c-1	GCGAACCTGTGAAGCCGAA	169	19
Pit-B2-1	GGCGGATTCTTGATGATGA	242	19
Pit-B1c-1	TCGGCAAGGAACCTGACAAC	170	20
Product	TCTCTACCGATGCCTCAATGAGCTCGGCTGCTGGCAAGCTCAACCGGAGATGGTGGTTCGGCTTCACAGGT TCGCTCGGCAAGGAACCTGACAACGGCCAAGTACAGATCAAGATCATCCCAGACGCGCTCATCATCAAGAAT CCGCC		148
Pit-F3-2	TTGTTCTGCTCATCGTGG	20	18
Pit-B3-2	ACCACCGTCTTGAATCTTG	314	19
Pit-FIP(F1c+F2)-2	GCGAACCTGTGAAGCCGAATCTCTACCGATGCCTCAAT		38
Pit-BIP(B1c+B2)-2	TCGGCAAGGAACCTGACAACGGCGGATTCTTGATGATGA		39
Pit-LoopF-2	CCACCATCTCCGGTTGAG	150	18
Pit-LoopB-2	GGCCAAGTACAGATCAAGATCA	190	22

Pit-F2-2	TCTCTACCGATGCCTCAAT	95	19
Pit-F1c-2	GCGAACCTGTGAAGCCGAA	169	19
Pit-B2-2	GGCGGATTCTTGATGATGA	242	19
Pit-B1c-2	TCGGCAAGGAACCTGACAAC	170	20
Product	TCTCTACCGATGCCTCAATGAGCTCGGCTGCTGGCAAGCTCAACCGGAGATGGTGGTTCGGCTTCACAGGTTCGCTCGGCAAGGAACCTGACAACGGCCAAGTACAGATCAAGATCATCCCAGACGCGCTCATCATCAAGAATCCGCC		148
Pit-F3-3	TTTCGCTCAGCCTTTGTT	7	18
Pit-B3-3	GGCGGATTCTTGATGATGA	242	19
Pit-FIP(F1c+F2)-3	GTTGAGCTTGCCAGCAGCCAACATGTTCAAGCTATTCCG		39
Pit-BIP(B1c+B2)-3	GTGGTTCGGCTTCACAGGTTGATCTTGATCTGTACTTGGC		40
Pit-LoopF-3	GCTCATTGAGGCATCGGTA	117	19
Pit-LoopB-3	CGGCAAGGAACCTGACAA	171	18
Pit-F2-3	CAACATGTTCAAGCTATTCCG	61	21
Pit-F1c-3	GTTGAGCTTGCCAGCAGC	138	18
Pit-B2-3	TGATCTTGATCTGTACTTGGC	211	21
Pit-B1c-3	GTGGTTCGGCTTCACAGGT	147	19
Product	CAACATGTTCAAGCTATTCCGGTGCGTCGATCGCTCTCTACCGATGCCTCAATGAGCTCGGCTGCTGGCAAGCTCAACCGGAGATGGTGGTTCGGCTTCACAGGTTCGCTCGGCAAGGAACCTGACAACGGCCAAGTACAGATCAAGATCA		151
Pit-F3-4	TCTCTACCGATGCCTCAA	95	18
Pit-B3-4	TTAGGATCTGTCGGCATGA	332	19
Pit-FIP(F1c+F2)-4	TTGTCAGGTTCCTTGCCGAGCAAGCTCAACCGGAGATG		38
Pit-BIP(B1c+B2)-4	CATCCCAGACGCGCTCATGATTAGCTTGTTCAGATCGTCT		40
Pit-LoopF-4	AACCTGTGAAGCCGAACC	166	18

Pit-LoopB-4	ATCAAGAATCCGCCTGCC	229	18
Pit-F2-4	CAAGCTCAACCGGAGATG	129	18
Pit-F1c-4	TTGTCAGGTTCCTTGCCGAG	188	20
Pit-B2-4	GATTAGCTTGTTCAGATCGTCT	273	22
Pit-B1c-4	CATCCCAGACGCGCTCAT	210	18
Product	CAAGCTCAACCGGAGATGGTGGTTCGGCTTCACAGGTTCGCTCGGCAAGGAACCTGACAACGGCCAAGTAC AGATCAAGATCATCCCAGACGCGCTCATCATCAAGAATCCGCCTGCCAACAAAGACGATCTGAACAAGCTAAT C		145
Pep-F3-1	GACTCGTGTACCAATGCC	328	18
Pep-B3-1	TACCGATTCCTCCTAGCAG	520	19
Pep-FIP(F1c+F2)-1	TGGCTTGAACCGCATCGTAAGGTATCTTCAATGTGATTGTGC		42
Pep-BIP(B1c+B2)-1	AGGGTGGCACTGGAGGAATCCTGGTCGTTAGAGTCTG		37
Pep-LoopF-1	GCCTGAGATCCAAGAGCATT	401	20
Pep-LoopB-1	CACTGACGACGACACCTC	444	18
Pep-F2-1	GGTATCTTCAATGTGATTGTGC	349	22
Pep-F1c-1	TGGCTTGAACCGCATCGTAA	421	20
Pep-B2-1	TCCTGGTCGTTAGAGTCTG	485	19
Pep-B1c-1	AGGGTGGCACTGGAGGAA	422	18
Product	GGTATCTTCAATGTGATTGTGCCCGCTTCGTCCAATGCTCTTGGATCTCAGGCTTACGATGCGGTTCAAGCCA AGGGTGGCACTGGAGGAACCGGCACTGACGACGACACCTCGGCCCCAGACTCTAACGACCAGGA		137
**Pep-F3-2**	TCGTGTACCAATGCCAAAG	331	19
**Pep-B3-2**	TACCGATTCCTCCTAGCAG	520	19
**Pep-FIP(F1c+F2)-2**	TGGCTTGAACCGCATCGTAAGTATCTTCAATGTGATTGTGCC		42
**Pep-BIP(B1c+B2)-2**	AGGGTGGCACTGGAGGAATCCTGGTCGTTAGAGTCTG		37

**Pep-LoopF-2**	GCCTGAGATCCAAGAGCATT	401	20
**Pep-LoopB-2**	CACTGACGACGACACCTC	444	18
**Pep-F2-2**	GTATCTTCAATGTGATTGTGCC	350	22
**Pep-F1c-2**	TGGCTTGAACCGCATCGTAA	421	20
**Pep-B2-2**	TCCTGGTCGTTAGAGTCTG	485	19
**Pep-B1c-2**	AGGGTGGCACTGGAGGAA	422	18
**Product**	GTATCTTCAATGTGATTGTGCCCGCTTCGTCCAATGCTCTTGGATCTCAGGCTTACGATGCGGTTCAAGCCAA GGGTGGCACTGGAGGAACCGGCACTGACGACGACACCTCGGCCCCAGACTCTAACGACCAGGA		136
Pep-F3-3	CAACAATTCGTACACACTGC	252	20
Pep-B3-3	TACCGATTCCTCCTAGCAG	520	19
Pep-FIP(F1c+F2)-3	CGTAAGCCTGAGATCCAAGAGCGACTCGTGTACCAATGCC		40
Pep-BIP(B1c+B2)-3	AAGGGTGGCACTGGAGGATCCTGGTCGTTAGAGTCTG		37
Pep-LoopF-3	CGGGCACAATCACATTGAAG	373	20
Pep-LoopB-3	CACTGACGACGACACCTC	444	18
Pep-F2-3	GACTCGTGTACCAATGCC	328	18
Pep-F1c-3	CGTAAGCCTGAGATCCAAGAGC	406	22
Pep-B2-3	TCCTGGTCGTTAGAGTCTG	485	19
Pep-B1c-3	AAGGGTGGCACTGGAGGA	421	18
Product	GACTCGTGTACCAATGCCAAAGGTATCTTCAATGTGATTGTGCCCGCTTCGTCCAATGCTCTTGGATCTCAGG CTTACGATGCGGTTCAAGCCAAGGGTGGCACTGGAGGAACCGGCACTGACGACGACACCTCGGCCCCAGA CTCTAACGACCAGGA		158
Pep-F3-4	AGGGACAACAATTCGTACAC	247	20
Pep-B3-4	TACCGATTCCTCCTAGCAG	520	19
Pep-FIP(F1c+F2)-4	CGTAAGCCTGAGATCCAAGAGCGCGACTCGTGTACCAATG		40
Pep-BIP(B1c+B2)-4	AAGGGTGGCACTGGAGGATCCTGGTCGTTAGAGTCTG		37

Pep-LoopF-4	CGGGCACAATCACATTGAAG	373	20
Pep-LoopB-4	CACTGACGACGACACCTC	444	18
Pep-F2-4	GCGACTCGTGTACCAATG	326	18
Pep-F1c-4	CGTAAGCCTGAGATCCAAGAGC	406	22
Pep-B2-4	TCCTGGTCGTTAGAGTCTG	485	19
Pep-B1c-4	AAGGGTGGCACTGGAGGA	421	18
Product	GCGACTCGTGTACCAATGCCAAAGGTATCTTCAATGTGATTGTGCCCGCTTCGTCCAATGCTCTTGGATCTCA GGCTTACGATGCGGTTCAAGCCAAGGGTGGCACTGGAGGAACCGGCACTGACGACGACACCTCGGCCCCA GACTCTAACGACCAGGA		160
mPep-F3-1	ACTGGTTCTCCTCGGTC	146	17
mPep-B3-1	TACCGATTCCTCCTAGCAG	535	19
mPep-FIP(F1c+F2)-1	AGGCACAATCACATTGAAGATACCTGGACAACAATTCGTACACA		44
mPep-BIP(B1c+B2)-1	CTCAGGCTTACGATGCGGTTTGTCGTCGTCAGTGC		35
mPep-LoopF-1	TGGCATTGGTACACGAGTC	346	19
mPep-LoopB-1	AGGAGCAGGCACTGGA	435	16
mPep-F2-1	GGACAACAATTCGTACACA	249	19
mPep-F1c-1	AGGCACAATCACATTGAAGATACCT	372	25
mPep-B2-1	TGTCGTCGTCAGTGC	472	15
mPep-B1c-1	CTCAGGCTTACGATGCGGTT	395	20
Product	CGCTCAAGCCGCTTGGAAAAGCATTTTCGAGCAACGTGCGCGACTCGTGTACCAATGCCAAAGGTATCTTC AATGTGATTGTGCCTGCTTCGTCCAATGCTCTTGGATCTCAGGCTTACGATGCGGTTCAAGCCAAGGGTGG CACCGGAGGAGCAGGCACTGGAGGAACCGGCACTGACGACGACA		224
**mPep-F3-2**	GACTCGTGTACCAATGCC	328	18
**mPep-B3-2**	TACCGATTCCTCCTAGCAG	535	19

**mPep-FIP(F1c+F2)-2**	TGGCTTGAACCGCATCGTAATGCCTGCTTCGTCCA		35
**mPep-BIP(B1c+B2)-2**	GTGGCACCGGAGGAGCTGTCGTCGTCAGTGC		31
**mPep-LoopF-2**	GCCTGAGATCCAAGAGCAT	401	19
**mPep-LoopB-2**	GGCACTGGAGGAACCG	442	16
**mPep-F2-2**	TGCCTGCTTCGTCCA	368	15
**mPep-F1c-2**	TGGCTTGAACCGCATCGTAA	421	20
**mPep-B2-2**	TGTCGTCGTCAGTGC	472	15
**mPep-B1c-2**	GTGGCACCGGAGGAGC	425	16
Product	TGCCTGCTTCGTCCAATGCTCTTGGATCTCAGGCTTACGATGCGGTTCAAGCCAAGGGTGGCACCGGAGGA GCAGGCACTGGAGGAACCGGCACTGACGACGACA		105
mPep-F3-3	GCGACTCGTGTACCAATG	326	18
mPep-B3-3	TACCGATTCCTCCTAGCAG	535	19
mPep-FIP(F1c+F2)-3	TGGCTTGAACCGCATCGTAAATTGTGCCTGCTTCGTC		37
mPep-BIP(B1c+B2)-3	GTGGCACCGGAGGAGCTGTCGTCGTCAGTGC		31
mPep-LoopF-3	GCCTGAGATCCAAGAGCATT	401	20
mPep-LoopB-3	GGCACTGGAGGAACCG	442	16
mPep-F2-3	ATTGTGCCTGCTTCGTC	364	17
mPep-F1c-3	TGGCTTGAACCGCATCGTAA	421	20
mPep-B2-3	TGTCGTCGTCAGTGC	472	15
mPep-B1c-3	GTGGCACCGGAGGAGC	425	16
Product	ATTGTGCCTGCTTCGTCCAATGCTCTTGGATCTCAGGCTTACGATGCGGTTCAAGCCAAGGGTGGCACCGG AGGAGCAGGCACTGGAGGAACCGGCACTGACGACGACA		109
mPep-F3-4	GCGACTCGTGTACCAAT	326	17
mPep-B3-4	TACCGATTCCTCCTAGCAG	535	19
mPep-FIP(F1c+F2)-4	TGGCTTGAACCGCATCGTAAGATTGTGCCTGCTTCGT		37

mPep-BIP(B1c+B2)-4	GTGGCACCGGAGGAGCTGTCGTCGTCAGTGC		31
mPep-LoopF-4	GCCTGAGATCCAAGAGCATT	401	20
mPep-LoopB-4	GGCACTGGAGGAACCG	442	16
mPep-F2-4	GATTGTGCCTGCTTCGT	363	17
mPep-F1c-4	TGGCTTGAACCGCATCGTAA	421	20
mPep-B2-4	TGTCGTCGTCAGTGC	472	15
mPep-B1c-4	GTGGCACCGGAGGAGC	425	16
Product	GATTGTGCCTGCTTCGTCCAATGCTCTTGGATCTCAGGCTTACGATGCGGTTCAAGCCAAGGGTGGCACCG GAGGAGCAGGCACTGGAGGAACCGGCACTGACGACGACA		110
mPep-F3-5	GCGACTCGTGTACCAA	326	16
mPep-B3-5	TACCGATTCCTCCTAGCAG	535	19
mPep-FIP(F1c+F2)-5	TGGCTTGAACCGCATCGTAATGATTGTGCCTGCTTCG		37
mPep-BIP(B1c+B2)-5	GTGGCACCGGAGGAGCTGTCGTCGTCAGTGC		31
mPep-LoopF-5	GCCTGAGATCCAAGAGCATT	401	20
mPep-LoopB-5	GGCACTGGAGGAACCG	442	16
mPep-F2-5	TGATTGTGCCTGCTTCG	362	17
mPep-F1c-5	TGGCTTGAACCGCATCGTAA	421	20
mPep-B2-5	TGTCGTCGTCAGTGC	472	15
mPep-B1c-5	GTGGCACCGGAGGAGC	425	16
Product	TGATTGTGCCTGCTTCGTCCAATGCTCTTGGATCTCAGGCTTACGATGCGGTTCAAGCCAAGGGTGGCACC GGAGGAGCAGGCACTGGAGGAACCGGCACTGACGACGACA		111
mPep-F3-6	GGACAACAATTCGTACACA	249	19
mPep-B3-6	TACCGATTCCTCCTAGCAG	535	19
mPep-FIP(F1c+F2)-6	AACCGCATCGTAAGCCTGAGGGTATCTTCAATGTGATTGTGC		42
mPep-BIP(B1c+B2)-6	GTGGCACCGGAGGAGCTGTCGTCGTCAGTGC		31

mPep-LoopF-6	AAGAGCATTGGACGAAGCA	390	19
mPep-LoopB-6	GGCACTGGAGGAACCG	442	16
mPep-F2-6	GGTATCTTCAATGTGATTGTGC	349	22
mPep-F1c-6	AACCGCATCGTAAGCCTGAG	414	20
mPep-B2-6	TGTCGTCGTCAGTGC	472	15
mPep-B1c-6	GTGGCACCGGAGGAGC	425	16
Product	GGTATCTTCAATGTGATTGTGCCTGCTTCGTCCAATGCTCTTGGATCTCAGGCTTACGATGCGGTTCAAGCC AAGGGTGGCACCGGAGGAGCAGGCACTGGAGGAACCGGCACTGACGACGACA		124
mPep-F3-7	CAAGCCACGCTTCTCT	19	16
mPep-B3-7	TACCGATTCCTCCTAGCAG	535	19
mPep-FIP(F1c+F2)-7	CGCCGACTTCGACCGAGCGTCCATGCCGATGC		32
mPep-BIP(B1c+B2)-7	CTCAGGCTTACGATGCGGTTTGTCGTCGTCAGTGC		35
mPep-LoopF-7	GGCAATGGTACCGCACC	104	17
mPep-LoopB-7	AGGAGCAGGCACTGGA	435	16
mPep-F2-7	CGTCCATGCCGATGC	69	15
mPep-F1c-7	CGCCGACTTCGACCGAG	172	17
mPep-B2-7	TGTCGTCGTCAGTGC	472	15
mPep-B1c-7	CTCAGGCTTACGATGCGGTT	395	20
Product	CGTCCATGCCGATGCTGCGGGTGCGGTACCATTGCCGAATTTCAAGGTAGACCCGCAACCTCTGGCCAGC ACGTTTTACTGGTTCTCCTCGGTCGAAGTCGGCGTTTGCTACAACCCGCAAGCTCGCGTTGGCAGCACCAA AGGCGCTCTTCACTGCACACACCAAGAAAACTACGACAGGGACAACAATTCGTACACACTGCCGCAAACGT GCGTGGCGCTCAAGCCGCTTGGAAAAGCATTTTCGAGCAACGTGCGCGACTCGTGTACCAATGCCAAAGGT ATCTTCAATGTGATTGTGCCTGCTTCGTCCAATGCTCTTGGATCTCAGGCTTACGATGCGGTTCAAGCCAAG GGTGGCACCGGAGGAGCAGGCACTGGAGGAACCGGCACTGACGACGACA		404

* Primers name in bold type indicates the optimum RealAmp primer sets Pep-2 and mPep-2 that designed for *U*. *maydis* of Clade A and Clade B, respectively.
