# Supplementary material for: Development of a real-time fluorescence loop-mediated isothermal amplification assay for rapid and quantitative detection of Ustilago maydis
Source: Sci Rep. 2017 Oct 17;7:13394. doi: 10.1038/s41598-017-13881-4 (PMC5645423; doi:10.1038/s41598-017-13881-4)
Supplement: Supplementary file 3 — Supplementary Table S2 [file 41598_2017_13881_MOESM3_ESM.pdf]

TITLE:

Development of a real-time fluorescence loop-mediated isothermal amplification assay for rapid and quantitative detection of *Ustilago maydis*

AUTHORS:

Yanyong Cao[1, +], Lifeng Wang[1, +], Liping Duan[2], Jingjing Li[1], Juan Ma[1], Shuna Xie[3], Lei Shi[2] & Huiyong Li[1]

AFFILIATION:

[1] Institute of Cereal Crops, Henan Academy of Agricultural Sciences, Zhengzhou, 450002, China

[2] Institute of Food Safety and Nutrition, Jinan University, Guangzhou, 510632, China

[3] Institute of Plant Protection, Henan Academy of Agricultural Sciences, Zhengzhou, 450002, China

Correspondence and requests for materials should be addressed to Y.C. (email: yanyongcao@126.com) or H.L. (email: lihuiyong1977@126.com)
+These authors contribute equally to this work.

Supplementary Table S2. Quantification of soil and maize plants samples by RealAmp assay using ZYD-S1 Soil Samples

Soil samples

Samples NO.	Field[a]	Tt	spores g-1soil			LAMP products amplification	Sequencing analysis[b]	Growth on media[c]	PCR with Pok317/Pok318	Sequencing analysis[d]
			Test 1	Test 2	Average					
1	A1	12.25±0.25	5.62E+04	9.45E+04	7.53E+04	+	Clade A	+	+	Clade A
2	A1	15±0	4.16E+03	4.16E+03	4.16E+03	+	Clade A	+	+	Clade A
3	A1	10.75±0.25	4.51E+05	2.68E+05	3.59E+05	+	Clade A	+	+	Clade A
4	A1	11.75±0.25	9.45E+04	1.59E+05	1.27E+05	+	Clade A	+	+	Clade A
5	A1	13±0	3.34E+04	3.34E+04	3.34E+04	+	Clade A	+	+	Clade A
6	A2	12.75±0.25	5.62E+04	3.34E+04	4.48E+04	+	Clade A	+	+	Clade A
7	A2	10.5±0	4.51E+05	4.51E+05	4.51E+05	+	Clade A	+	+	Clade A
8	A2	14.5±0	7.00E+03	7.00E+03	7.00E+03	+	Clade A	+	+	Clade A
9	A2	11.5±0.5	2.68E+05	9.45E+04	1.81E+05	+	Clade A	+	+	Clade A
10	A2	11.75±0.25	1.59E+05	9.45E+04	1.27E+05	+	Clade A	+	+	Clade A
11	A3	15.5±0.5	4.16E+03	1.47E+03	2.81E+03	+	Clade A	+	+	Clade A
12	A3	15.25±0.25	4.16E+03	2.47E+03	3.31E+03	+	Clade A	+	+	Clade A
13	A3	15±0	4.16E+03	4.16E+03	4.16E+03	+	Clade A	+	+	Clade A
14	A3	15.25±0.25	2.47E+03	4.16E+03	3.31E+03	+	Clade A	+	+	Clade A
15	A3	14.25±0.25	7.00E+03	1.18E+04	9.39E+03	+	Clade A	+	+	Clade A
16	A4	11.25±0.25	1.59E+05	2.68E+05	2.13E+05	+	Clade A	+	+	Clade A
17	A4	12.25±0.25	5.62E+04	9.45E+04	7.53E+04	+	Clade A	+	+	Clade A
18	A4	10.5±0	4.51E+05	4.51E+05	4.51E+05	+	Clade A	+	+	Clade A
19	A4	12.75±0.25	3.34E+04	5.62E+04	4.48E+04	+	Clade A	+	+	Clade A
20	A4	NA	-	-	-	-	N/A	-	-	N/A

21	A5	12.75±0.25	3.34E+04	5.62E+04	4.48E+04	+	Clade A	+	+	Clade A
22	A5	10.25±0.25	4.51E+05	7.59E+05	6.05E+05	+	Clade A	+	+	Clade A
23	A5	9.25±0.25	1.28E+06	2.15E+06	1.71E+06	+	Clade A	+	+	Clade A
24	A5	12.25±0.25	5.62E+04	9.45E+04	7.53E+04	+	Clade A	+	+	Clade A
25	A5	N/A	-	-	-	-	N/A	-	-	N/A
26	B1	N/A	-	-	-	-	N/A	-	-	N/A
27	B1	N/A	-	-	-	-	N/A	-	-	N/A
28	B1	N/A	-	-	-	-	N/A	-	-	N/A
29	B1	N/A	-	-	-	-	N/A	-	-	N/A
30	B1	N/A	-	-	-	-	N/A	-	-	N/A
31	B2	13.25±0.25	1.98E+04	3.34E+04	2.66E+04	+	Clade A	+	+	Clade A
32	B2	13.25±0.25	1.98E+04	3.34E+04	2.66E+04	+	Clade A	+	+	Clade A
33	B2	7.5±0	1.03E+07	1.03E+07	1.03E+07	+	Clade A	+	+	Clade A
34	B2	8.25±0.25	3.62E+06	6.09E+06	4.86E+06	+	Clade A	+	+	Clade A
35	B2	14.5±0	7.00E+03	7.00E+03	7.00E+03	+	Clade A	+	+	Clade A
36	B3	13.35±0.15	1.98E+04	2.71E+04	2.35E+04	+	Clade A	+	+	Clade A
37	B3	10.5±0	4.51E+05	4.51E+05	4.51E+05	+	Clade A	+	+	Clade A
38	B3	N/A	-	-	-	-	N/A	-	-	N/A
39	B3	11.5±0	1.59E+05	1.59E+05	1.59E+05	+	Clade A	+	+	Clade A
40	B3	12.5±0.5	9.45E+04	3.34E+04	6.39E+04	+	Clade A	+	+	Clade A
41	B4	10.75±0.25	4.51E+05	2.68E+05	3.59E+05	+	Clade A	+	+	Clade A
42	B4	10.75±0.25	2.68E+05	4.51E+05	3.59E+05	+	Clade A	+	+	Clade A
43	B4	14.75±0.25	7.00E+03	4.16E+03	5.58E+03	+	Clade A	+	+	Clade A
44	B4	14.75±0.25	7.00E+03	4.16E+03	5.58E+03	+	Clade A	+	+	Clade A
45	B4	13.75±0.25	1.98E+04	1.18E+04	1.58E+04	+	Clade A	+	+	Clade A

46	B5	8.75±0.25	2.15E+06	3.62E+06	2.88E+06	+	Clade A	+	+	Clade A
47	B5	12.25±0.25	9.45E+04	5.62E+04	7.53E+04	+	Clade A	+	+	Clade A
48	B5	8.75±0.25	3.62E+06	2.15E+06	2.88E+06	+	Clade A	+	+	Clade A
49	B5	10.5±0	4.51E+05	4.51E+05	4.51E+05	+	Clade A	+	+	Clade A
50	B5	N/A	-	-	-	-	N/A	-	-	N/A
51	C1	13.75±0.25	1.98E+04	1.18E+04	1.58E+04	+	Clade A	+	+	Clade A
52	C1	13.5±0	1.98E+04	1.98E+04	1.98E+04	+	Clade A	+	+	Clade A
53	C1	12.25±0.25	5.62E+04	9.45E+04	7.53E+04	+	Clade A	+	+	Clade A
54	C1	N/A	-	-	-	-	N/A	-	-	N/A
55	C1	14.75±0.25	7.00E+03	4.16E+03	5.58E+03	+	Clade A	+	+	Clade A
56	C2	10.75±0.25	4.51E+05	2.68E+05	3.59E+05	+	Clade A	+	+	Clade A
57	C2	10±0	7.59E+05	7.59E+05	7.59E+05	+	Clade A	+	+	Clade A
58	C2	13.25±0.25	1.98E+04	3.34E+04	2.66E+04	+	Clade A	+	+	Clade A
59	C2	10±0.5	1.28E+06	4.51E+05	8.64E+05	+	Clade A	+	+	Clade A
60	C2	13.25±0.25	1.98E+04	3.34E+04	2.66E+04	+	Clade A	+	+	Clade A
61	C3	N/A	-	-	-	-	N/A	-	-	N/A
62	C3	12.25±0.25	5.62E+04	9.45E+04	7.53E+04	+	Clade A	+	+	Clade A
63	C3	15.25±0.25	4.16E+03	2.47E+03	3.31E+03	+	Clade A	+	+	Clade A
64	C3	13.25±0.25	1.98E+04	3.34E+04	2.66E+04	+	Clade A	+	+	Clade A
65	C3	15.75±0.25	2.47E+03	1.47E+03	1.97E+03	+	Clade A	+	+	Clade A
66	C4	N/A	-	-	-	-	N/A	-	-	N/A
67	C4	N/A	-	-	-	-	N/A	-	-	N/A
68	C4	15±0.5	7.00E+03	2.47E+03	4.73E+03	+	Clade A	+	+	Clade A
69	C4	16±0.5	2.47E+03	8.72E+02	1.67E+03	+	Clade A	+	+	Clade A
70	C4	11±0.5	4.51E+05	1.59E+05	3.05E+05	+	Clade A	+	+	Clade A

71	C5	N/A	-	-	-	-	N/A	-	-	N/A
72	C5	11.75±0.25	1.59E+05	9.45E+04	1.27E+05	+	Clade A	+	+	Clade A
73	C5	9±0.5	3.62E+06	1.28E+06	2.45E+06	+	Clade A	+	+	Clade A
74	C5	11.75±0.25	1.59E+05	9.45E+04	1.27E+05	+	Clade A	+	+	Clade A
75	C5	12.5±0.5	9.45E+04	3.34E+04	6.39E+04	+	Clade A	+	+	Clade A
76	D1	N/A	-	-	-	-	N/A	-	-	N/A
77	D1	N/A	-	-	-	-	N/A	-	-	N/A
78	D1	N/A	-	-	-	-	N/A	-	-	N/A
79	D1	N/A	-	-	-	-	N/A	-	-	N/A
80	D1	N/A	-	-	-	-	N/A	-	-	N/A
81	D2	13.75±0.25	1.98E+04	1.18E+04	1.58E+04	+	Clade A	+	+	Clade A
82	D2	12.75±0.25	5.62E+04	3.34E+04	4.48E+04	+	Clade A	+	+	Clade A
83	D2	14.5±0.5	1.18E+04	4.16E+03	7.97E+03	+	Clade A	+	+	Clade A
84	D2	13.75±0.25	1.18E+04	1.98E+04	1.58E+04	+	Clade A	+	+	Clade A
85	D2	13.5±0.5	3.34E+04	1.18E+04	2.26E+04	+	Clade A	+	+	Clade A
86	D3	N/A	-	-	-	-	N/A	-	-	N/A
87	D3	N/A	-	-	-	-	N/A	-	-	N/A
88	D3	12.25±0.25	5.62E+04	9.45E+04	7.53E+04	+	Clade A	+	+	Clade A
89	D3	14±0.5	7.00E+03	1.98E+04	1.34E+04	+	Clade A	+	+	Clade A
90	D3	12.25±0.25	5.62E+04	9.45E+04	7.53E+04	+	Clade A	+	+	Clade A
91	D4	11.25±0.25	1.59E+05	2.68E+05	2.13E+05	+	Clade A	+	+	Clade A
92	D4	13±0.5	5.62E+04	1.98E+04	3.80E+04	+	Clade A	+	+	Clade A
93	D4	10.5±0.5	7.59E+05	2.68E+05	5.13E+05	+	Clade A	+	+	Clade A
94	D4	10±0.5	1.28E+06	4.51E+05	8.64E+05	+	Clade A	+	+	Clade A
95	D4	12±0.5	1.59E+05	5.62E+04	1.08E+05	+	Clade A	+	+	Clade A

96	D5	11.5±0.5	2.68E+05	9.45E+04	1.81E+05	+	Clade A	+	+	Clade A
97	D5	12.5±0.5	9.45E+04	3.34E+04	6.39E+04	+	Clade A	+	+	Clade A
98	D5	14.5±0.5	1.18E+04	4.16E+03	7.97E+03	+	Clade A	+	+	Clade A
99	D5	10.75±0.25	2.68E+05	4.51E+05	3.59E+05	+	Clade A	+	+	Clade A
100	D5	12.5±0.5	9.45E+04	3.34E+04	6.39E+04	+	Clade A	+	+	Clade A
101	E1	11.75±0.25	1.59E+05	9.45E+04	1.27E+05	+	Clade B	+	+	Clade B
102	E1	10.5±0	4.51E+05	4.51E+05	4.51E+05	+	Clade B	+	+	Clade B
103	E1	12.75±0.25	5.62E+04	3.34E+04	4.48E+04	+	Clade B	+	+	Clade B
104	E1	10.35±0.35	7.59E+05	3.66E+05	5.62E+05	+	Clade B	+	+	Clade B
105	E1	9.75±0.25	1.28E+06	7.59E+05	1.02E+06	+	Clade B	+	+	Clade B
106	E2	14.75±0.25	7.00E+03	4.16E+03	5.58E+03	+	Clade B	+	+	Clade B
107	E2	84.25±69.75	7.00E+03	5.64E-60	3.50E+03	+	Clade B	+	+	Clade B
108	E2	12.25±0.25	5.62E+04	9.45E+04	7.53E+04	+	Clade B	+	+	Clade B
109	E2	15.75±0.25	2.47E+03	1.47E+03	1.97E+03	+	Clade B	+	+	Clade B
110	E2	14.25±0.25	7.00E+03	1.18E+04	9.39E+03	+	Clade B	+	+	Clade B
111	E3	13.75±0.25	1.98E+04	1.18E+04	1.58E+04	+	Clade B	+	+	Clade B
112	E3	10.25±0.25	4.51E+05	7.59E+05	6.05E+05	+	Clade B	+	+	Clade B
113	E3	10±0	7.59E+05	7.59E+05	7.59E+05	+	Clade B	+	+	Clade B
114	E3	9.25±0.25	2.15E+06	1.28E+06	1.71E+06	+	Clade B	+	+	Clade B
115	E3	11±0.5	1.59E+05	4.51E+05	3.05E+05	+	Clade B	+	+	Clade B
116	E4	12±0.5	1.59E+05	5.62E+04	1.08E+05	+	Clade B	+	+	Clade B
117	E4	8.5±0	3.62E+06	3.62E+06	3.62E+06	+	Clade B	+	+	Clade B
118	E4	7.75±0.25	1.03E+07	6.09E+06	8.17E+06	+	Clade B	+	+	Clade B
119	E4	12.25±0.25	5.62E+04	9.45E+04	7.53E+04	+	Clade B	+	+	Clade B
120	E4	10.25±0.25	4.51E+05	7.59E+05	6.05E+05	+	Clade B	+	+	Clade B

121	E5	12±0	9.45E+04	9.45E+04	9.45E+04	+	Clade B	+	+	Clade B
122	E5	11±0	2.68E+05	2.68E+05	2.68E+05	+	Clade B	+	+	Clade B
123	E5	10.5±0	4.51E+05	4.51E+05	4.51E+05	+	Clade B	+	+	Clade B
124	E5	12.25±0.25	5.62E+04	9.45E+04	7.53E+04	+	Clade B	+	+	Clade B
125	E5	12±0	9.45E+04	9.45E+04	9.45E+04	+	Clade B	+	+	Clade B
126	F1	14.25±0.25	7.00E+03	1.18E+04	9.39E+03	+	Clade A	+	+	Clade A
127	F1	8.75±0.25	2.15E+06	3.62E+06	2.88E+06	+	Clade A	+	+	Clade A
128	F1	11.5±0	1.59E+05	1.59E+05	1.59E+05	+	Clade A	+	+	Clade A
129	F1	12±0	9.45E+04	9.45E+04	9.45E+04	+	Clade A	+	+	Clade A
130	F1	14.75±0.25	7.00E+03	4.16E+03	5.58E+03	+	Clade A	+	+	Clade A
131	F2	9.5±0.5	2.15E+06	7.59E+05	1.45E+06	+	Clade A	+	+	Clade A
132	F2	10.75±0.25	4.51E+05	2.68E+05	3.59E+05	+	Clade A	+	+	Clade A
133	F2	9.25±0.25	1.28E+06	2.15E+06	1.71E+06	+	Clade A	+	+	Clade A
134	F2	11.75±0.25	1.59E+05	9.45E+04	1.27E+05	+	Clade A	+	+	Clade A
135	F2	12.25±0.25	5.62E+04	9.45E+04	7.53E+04	+	Clade A	+	+	Clade A
136	F3	11±0	2.68E+05	2.68E+05	2.68E+05	+	Clade A	+	+	Clade A
137	F3	12±0	9.45E+04	9.45E+04	9.45E+04	+	Clade A	+	+	Clade A
138	F3	11.75±0.25	1.59E+05	9.45E+04	1.27E+05	+	Clade A	+	+	Clade A
139	F3	9.5±0	1.28E+06	1.28E+06	1.28E+06	+	Clade A	+	+	Clade A
140	F3	17.5±0	3.08E+02	3.08E+02	3.08E+02	+	Clade A	+	+	Clade A
141	F4	13.75±0.25	1.18E+04	1.98E+04	1.58E+04	+	Clade A	+	+	Clade A
142	F4	12.75±0.25	5.62E+04	3.34E+04	4.48E+04	+	Clade A	+	+	Clade A
143	F4	11.25±0.25	1.59E+05	2.68E+05	2.13E+05	+	Clade A	+	+	Clade A
144	F4	13±0.5	5.62E+04	1.98E+04	3.80E+04	+	Clade A	+	+	Clade A
145	F4	10.5±0.5	7.59E+05	2.68E+05	5.13E+05	+	Clade A	+	+	Clade A

146	F5	10.5±0	4.51E+05	4.51E+05	4.51E+05	+	Clade A	+	+	Clade A
147	F5	11.25±0.25	1.59E+05	2.68E+05	2.13E+05	+	Clade A	+	+	Clade A
148	F5	11.75±0.25	1.59E+05	9.45E+04	1.27E+05	+	Clade A	+	+	Clade A
149	F5	13±0	3.34E+04	3.34E+04	3.34E+04	+	Clade A	+	+	Clade A
150	F5	14.25±0.25	1.18E+04	7.00E+03	9.39E+03	+	Clade A	+	+	Clade A

N/A, not determined.

+, positive result.

-, negative result.

a Filed location.

b Analysis of the sequencing of the LAMP products that amplified with primer sets F2-2/B2-2 or mF2-2/mB2-2.

c Growth on the PDA and $YEPS_L$.

d Analysis of the sequencing of the PCR products that amplified with primers Pok317/Pok318[31].

A, Qiliying town, Xinxiang County, Henan Province

B, Duliang town, Kaifeng City, Henan Province

C, Pohu town, Xuchang City, Henan Province

D, Wucheng town, Wuyang County, Henan Province

E, Baima town, Nanjing, Jiangsu Province

F, Zhengfu town, Fuyang City, Anhui Province

Maize plants samples

Samples NO.	Sample name[a]	Tt	U. maydis (ng/μL)			LAMP products amplification	Sequencing analysis[b]	Growth on media[c]	PCR with Pok317/Pok318	sequencing analysis[d]
			Test 1	Test 2	Average					
1	A-1	22±0	0.248995	0.248995	0.248995	+	Clade A	+	+	Clade A
2	A-2	18±0.5	1.3130764	1.0766139	1.1948451	+	Clade A	+	+	Clade A
3	A-3	18.5±0	1.0766139	1.0766139	1.0766139	+	Clade A	+	+	Clade A
4	A-4	22.5±0.5	0.248995	0.0125325	0.1307638	+	Clade A	+	+	Clade A
5	A-5	20±0.5	0.8401513	0.6036888	0.7219201	+	Clade A	+	+	Clade A
6	A-6	22.75±0.25	0.1307638	0.0125325	0.0716481	+	Clade A	+	+	Clade A
7	A-7	22.5±0.5	0.0125325	0.248995	0.1307638	+	Clade A	+	+	Clade A
8	A-8	17.25±0.25	1.3130764	1.4313076	1.372192	+	Clade A	+	+	Clade A
9	A-9	22.5±0.5	0.0125325	0.248995	0.1307638	+	Clade A	+	+	Clade A
10	B1-1	22±0.5	0.1307638	0.3672263	0.248995	+	Clade A	+	+	Clade A
11	B1-2	21±0.5	0.6036888	0.3672263	0.4854576	+	Clade A	+	+	Clade A
12	B1-3	22.75±0.25	0.0125325	0.1307638	0.0716481	+	Clade A	+	+	Clade A
13	B1-4	17±0	1.4313076	1.4313076	1.4313076	+	Clade A	+	+	Clade A
14	B1-5	15.5±0.5	1.9042327	1.6677702	1.7860014	+	Clade A	+	+	Clade A
15	B1-6	11.75±0.25	2.7318515	2.6136202	2.6727359	+	Clade A	+	+	Clade A
16	B1-7	13.25±0.25	2.3771577	2.2589265	2.3180421	+	Clade A	+	+	Clade A
17	B1-8	19±0	0.9583826	0.9583826	0.9583826	+	Clade A	+	+	Clade A
18	B1-9	18.25±0.25	1.1948451	1.0766139	1.1357295	+	Clade A	+	+	Clade A
19	B2-1	18.75±0.25	0.9583826	1.0766139	1.0174982	+	Clade A	+	+	Clade A
20	B2-2	16.75±0.25	1.5495389	1.4313076	1.4904233	+	Clade A	+	+	Clade A

21	B2-3	17±0.5	1.3130764	1.5495389	1.4313076	+	Clade A	+	+	Clade A
22	B2-4	13.75±0.25	2.2589265	2.1406952	2.1998108	+	Clade A	+	+	Clade A
23	B2-5	18±0.5	1.0766139	1.3130764	1.1948451	+	Clade A	+	+	Clade A
24	B2-6	15.25±0.25	1.7860014	1.9042327	1.845117	+	Clade A	+	+	Clade A
25	B2-7	18.5±0	1.0766139	1.0766139	1.0766139	+	Clade A	+	+	Clade A
26	B2-8	13.25±0.25	2.2589265	2.3771577	2.3180421	+	Clade A	+	+	Clade A
27	B2-9	20.25±0.25	0.6036888	0.7219201	0.6628044	+	Clade A	+	+	Clade A
28	C-1	22.25±0.25	0.248995	0.1307638	0.1898794	+	Clade A	+	+	Clade A
29	C-2	21.5±0.5	0.248995	0.4854576	0.3672263	+	Clade A	+	+	Clade A
30	C-3	22.75±0.25	0.0125325	0.1307638	0.0716481	+	Clade A	+	+	Clade A
31	C-4	23±0	0.0125325	0.0125325	0.0125325	+	Clade A	+	+	Clade A
32	C-5	22.5±0.5	0.248995	0.0125325	0.1307638	+	Clade A	+	+	Clade A
33	C-6	16.25±0.25	1.6677702	1.5495389	1.6086545	+	Clade A	+	+	Clade A
34	C-7	18.5±0.5	1.1948451	0.9583826	1.0766139	+	Clade A	+	+	Clade A
35	C-8	20.25±0.25	0.7219201	0.6036888	0.6628044	+	Clade A	+	+	Clade A
36	C-9	16.25±0.25	1.5495389	1.6677702	1.6086545	+	Clade A	+	+	Clade A
37	D-1	20.5±0	0.6036888	0.6036888	0.6036888	+	Clade A	+	+	Clade A
38	D-2	22±0.5	0.1307638	0.3672263	0.248995	+	Clade A	+	+	Clade A
39	D-3	22.75±0.25	0.1307638	0.0125325	0.0716481	+	Clade A	+	+	Clade A
40	D-4	20.5±0.5	0.7219201	0.4854576	0.6036888	+	Clade A	+	+	Clade A
41	D-5	21±0.5	0.3672263	0.6036888	0.4854576	+	Clade A	+	+	Clade A
42	D-6	14.5±0.5	2.1406952	1.9042327	2.0224639	+	Clade A	+	+	Clade A
43	D-7	14.25±0.25	2.1406952	2.0224639	2.0815796	+	Clade A	+	+	Clade A
44	D-8	14±0.5	2.2589265	2.0224639	2.1406952	+	Clade A	+	+	Clade A
45	D-9	19.5±0	0.8401513	0.8401513	0.8401513	+	Clade A	+	+	Clade A

46	M-1	N/A	-	-	-	-	N/A	-	-	N/A
47	M-2	N/A	-	-	-	-	N/A	-	-	N/A
48	M-3	20.25±0.25	0.7219201	0.6036888	0.6628044	+	Clade A	+	+	Clade A
49	M-4	18.5±0	1.0766139	1.0766139	1.0766139	+	Clade A	+	+	Clade A
50	M-5	22.5±0.5	0.0125325	0.248995	0.1307638	+	Clade A	+	+	Clade A
51	M-6	20.75±0.25	0.6036888	0.4854576	0.5445732	+	Clade A	+	+	Clade A
52	M-7	16.25±0.25	1.5495389	1.6677702	1.6086545	+	Clade A	+	+	Clade A
53	M-8	15.5±0	1.7860014	1.7860014	1.7860014	+	Clade A	+	+	Clade A
54	M-9	15±0.5	2.0224639	1.7860014	1.9042327	+	Clade A	+	+	Clade A
55	S-1	N/A	-	-	-	-	N/A	-	-	N/A
56	S-2	N/A	-	-	-	-	N/A	-	-	N/A
57	S-3	N/A	-	-	-	-	N/A	-	-	N/A
58	S-4	N/A	-	-	-	-	N/A	-	-	N/A
59	S-5	N/A	-	-	-	-	N/A	-	-	N/A
60	S-6	N/A	-	-	-	-	N/A	-	-	N/A
61	S-7	N/A	-	-	-	-	N/A	-	-	N/A
62	S-8	N/A	-	-	-	-	N/A	-	-	N/A
63	S-9	N/A	-	-	-	-	N/A	-	-	N/A
64	Z-1	22.25±0.25	0.1307638	0.248995	0.1898794	+	Clade A	+	+	Clade A
65	Z-2	17.75±0.25	1.3130764	1.1948451	1.2539607	+	Clade A	+	+	Clade A
66	Z-3	19.25±0.25	0.8401513	0.9583826	0.899267	+	Clade A	+	+	Clade A
67	Z-4	18.75±0.25	1.0766139	0.9583826	1.0174982	+	Clade A	+	+	Clade A
68	Z-5	20.75±0.25	0.6036888	0.4854576	0.5445732	+	Clade A	+	+	Clade A
69	Z-6	18±0	1.1948451	1.1948451	1.1948451	+	Clade A	+	+	Clade A
70	Z-7	16.5±0.5	1.4313076	1.6677702	1.5495389	+	Clade A	+	+	Clade A

71	Z-8	15±0.5	2.0224639	1.7860014	1.9042327	+	Clade A	+	+	Clade A
72	Z-9	21.25±0.25	0.3672263	0.4854576	0.4263419	+	Clade A	+	+	Clade A

N/A, not determined.

+, positive result.

-, negative result.

a Genotypes of maize plants samples.

b Analysis of the sequencing of the LAMP products that amplified with primer sets F2-2/B2-2 or mF2-2/mB2-2.

c Growth on the PDA and $YEPS_L$.

d Analysis of the sequencing of the PCR products that amplified with primers Pok317/Pok318[31].

A, A801.

B1, B73.

B2, B104.

C, Chang7-2.

D, Dan340.

M, Mo17.

S, Shen137.

Z, Zheng58.
